# A transcriptome-based protein network that identifies new therapeutic targets in colorectal cancer

**DOI:** 10.1186/s12864-017-4139-y

**Published:** 2017-09-30

**Authors:** Stéphanie Durand, Killian Trillet, Arnaud Uguen, Aude Saint-Pierre, Catherine Le Jossic-Corcos, Laurent Corcos

**Affiliations:** 1 INSERM 1078 Unit, “Cancérologie appliquée et épissage alternatif” team, Brest Institute of Health, Agronomy and Material (IBSAM), Faculty of medicine, University of Western Brittany (UBO), 22 avenue Camille Desmoulins, F-29200 Brest, France; 2 Department of Pathology, Brest University Hospital, F-29200 Brest, France; 3 INSERM 1078 Unit, “Epidemiology, genetic Epidemiology and population genetics” team, 46 rue Félix Le Dantec, F-29200 Brest, France; 4 INSERM 1078 Unit, “Cancérologie appliquée et épissage alternatif” laboratory, University of Western Brittany (UBO), Faculty of medicine, 22, rue Camille Desmoulins, 29200 Brest, France

**Keywords:** Colorectal cancer, Transcriptome, Protein network, Therapeutic target

## Abstract

**Background:**

Colon cancer occurrence is increasing worldwide, making it the third most frequent cancer. Although many therapeutic options are available and quite efficient at the early stages, survival is strongly decreased when the disease has spread to other organs. The identification of molecular markers of colon cancer is likely to help understanding its course and, eventually, to uncover novel genes to be targeted by drugs. In this study, we compared gene expression in a set of 95 human colon cancer samples to that in 19 normal colon mucosae, focusing on 401 genes from 5 selected pathways (Apoptosis, Cancer, Cholesterol metabolism and lipoprotein signaling, Drug metabolism, Wnt/beta-catenin). Deregulation of mRNA levels largely matched that of proteins, leading us to build in silico protein networks, starting from mRNA levels, to identify key proteins central to network activity.

**Results:**

Among the analyzed genes, 10.5% (42) had no reported link with colon cancer, including the *SFRP1*, *IGF1* and *ADH1B* (down), and *MYC* and *IL8* (up), whose encoded proteins were most interacting with other proteins from the same or even distinct networks. Analyzing all pathways globally led us to uncover novel functional links between a priori unrelated or rather remotely connected pathways, such as the Drug metabolism and the Cancer pathways or, even more strikingly, between the Cholesterol metabolism and lipoprotein signaling and the Cancer pathways. In addition, we analyzed the responsiveness of some of the deregulated genes essential to network activities, to chemotherapeutic agents used alone or in presence of Lovastatin, a lipid-lowering drug. Some of these treatments could oppose the deregulations occurring in cancer samples, including those of the *CHECK2, CYP51A1, HMGCS1, ITGA2*, *NME1* or *VEGFA* genes.

**Conclusions:**

Our network-based approach allowed discovering genes not previously known to play regulatory roles in colon cancer. Our results also showed that selected drug treatments might revert the cancer-specific deregulation of genes playing prominent roles within the networks operating to maintain colon homeostasis. Among those genes, some could constitute novel testable targets to eliminate colon cancer cells, either directly or, potentially, through the use of lipid-lowering drugs such as statins, in association with selected anticancer drugs.

**Electronic supplementary material:**

The online version of this article (10.1186/s12864-017-4139-y) contains supplementary material, which is available to authorized users.

## Background

Colorectal cancer (CRC) is the third most common cancer type, and affords several hundred thousand deaths each year [1]. The implementation of screening methods has contributed significantly to reduce mortality, thanks to the early detection of precursor lesions [2]. Many curative options are available, essentially at the early stages, but survival drops down to a few percent in case of metastases. The amount of knowledge that has accumulated for this cancer is rather large, yet the means to prevent its development are dramatically insufficient. The temporality of genetic and epigenetic changes that occur in the course of CRC onset have been largely documented, including data contributed recently by the TCGA consortium, and CRC-enriched signaling pathways have been identified [3]. Many gene expression studies have been performed with whole transcriptome coverage by DNA chips and, more recently, by RNA sequencing methods [4, 5]. A recent study analyzed a reported data set distinguishing colon cancer from healthy samples with the Gene Ontology (GO) and the Kyoto Encyclopedia of Genes and Genomes (KEGG), and built a protein-protein interaction network using the Cytoscape environment [6]. Such a network analysis allowed identifying central proteins, or hubs, belonging to over- or under-represented pathways. In silico protein network construction can indeed provide sensible information on homeostasis disruption linked to disease [7, 8]. Several parameters can be characterized, which help establishing hierarchies between proteins from a network or even belonging to a priori distantly related networks [9]. Functional interactions existing between the products of deregulated genes can add to the sole knowledge of fold-change deregulation, by highlighting relationships between genes and the presence of hub genes that concentrate information transmission. Such a strategy can bring to light potential novel targets towards which corrective approaches may be developed. In this study, we used a dedicated PCR approach to identify putative novel gene expression markers relevant for colon cancer. We selected specific PCR arrays from Qiagen™, representing 5 specific signaling pathways, to compare 95 individual CRC samples to a set of 19 normal mucosae. These assays analyzed the Apoptosis, Cancer, Cholesterol metabolism and lipoprotein signaling, Drug metabolism and Wnt/beta-catenin pathways. Such a PCR-based approach, although of relatively low throughput, has the advantage to allow investigating directly, in a robust and straightforward analysis, the expression of genes involved in a given functional pathway, in this case most linked to cancer. These data led us to build up protein networks into the Cytoscape environment. A number of hierarchical links involving most of the proteins, some with key positions, both within a given pathway and across pathways, were identified. We next treated human CRC cell lines by anticancer drugs alone or in combination with Lovastatin, a cholesterol-lowering drug that can efficiently trigger apoptosis, and analyzed marker gene expression in response to the drugs. This led us to sort out genes that responded to treatments in a way that would lead to restoring gene expression as it stands in non-cancerous cells. On the whole, we believe that this work based on an integrative approach, in addition to identifying new deregulated gene expression networks in colon cancer, highlighted some interactions between the protein products of these genes, and identified genes whose cancer-specific expression could be reverted by pharmacological agents already used in the treatment of patients, or that may be so by other drugs like Lovastatin.

## Methods

### Tissue samples processing

Colon and rectum tissue samples were obtained after surgical removal and informed patient consent. The samples were processed anonymously: 95 colorectal carcinomas (CRC) and 19 normal tissues (NT) were collected between 2005 and 2011. Detailed patient information is presented in Table 1. Additional patient characteristics, such as potential comorbidity conditions at time of diagnosis (obesity, drug absorption, tobacco exposure or alcohol consumption, etc.) were not available. H/E staining was performed for all samples, and the tumor content within cancer samples was above 80%. Repartition of low grade (stages 0, I and II) and high-grade carcinoma samples (stages III and IV) was homogeneous. The tissue fragments were stored in RNAlater® stabilization solution (Ambion, France), a reagent that prevents mRNA from degradation. Total RNA was extracted with the AllPrep DNA/RNA Mini kit (Qiagen, Courtaboeuf, France) from homogenized tissue samples (20 mg). RNA purity and integrity were evaluated by measuring the optical density ratio (A260/A280) and the RNA Integrity Number (RIN) using the RNA 6000 Nano LabChip and the 2100 BioAnalyzer (Agilent, Massy, France), respectively. Only RNA samples with a 28S/18S ratio > 1.0 and RIN > 5.0 were used.

**Table 1:** Characteristics of the colorectal cancer patients used in the present study. Clinical and pathological information from normal and cancer samples were indicated according to number, sex ratio, age of patient, location and stage grading

		PCR array (Paired)		PCR array (All)			Western Blot (Paired)	
Group		NT	CRC	NT	CRC	CRC^a^	NT	CRC
Number of samples		17	17	19	95	75	27	27
Sex Ratio		11 M / 6 W		13 M / 6 W	57 M / 38 W	45 M / 30 W	14 M / 13 W	
Mean Age (±SD)		72 (± 3.1)		73 (± 2.8)	69 (± 1.4)	70 (± 1.5)	70 (± 2.5)	
Location		15 colon / 2 rectum		16 colon / 3 rectum	73 colon / 21 rectum	56 colon / 19 rectum	23 colon / 4 rectum	
Stage	0	/	0 (0%)	/	2 (2%)	1 (1%)	/	0 (0%)
	I	/	3 (18%)	/	16 (17%)	14 (19%)	/	6 (22%)
	II	/	4 (24%)	/	30 (31%)	25 (33%)	/	6 (22%)
	III	/	8 (47%)	/	28 (30%)	21 (28%)	/	7 (26%)
	IV	/	2 (12%)	/	19 (20%)	14 (19%)	/	8 (30%)

*Abbreviations*: *NT* Colorectal normal tissue, *CRC* colorectal carcinoma, *M* Man, *W* Women

^a^Only for analysis with the “Lipoprotein signaling and cholesterol metabolism” PCR array (the 75 samples were among the set of 95 CRC)

### Cultured cell lines and viability assay

HT-29 cells were cultured in Dulbecco’s modified Eagle’s medium (DMEM; 4.5 g/L glucose) (Lonza, Belgium) supplemented with 10% fetal bovine serum (FBS) (Gibco Invitrogen, U.S.A.), and HCT-116 cells were maintained in Dulbecco’s modified Eagle’s medium: Nutrient Mixture F-12 (DMEM/F-12) (Lonza, Belgium), supplemented with 5% FBS. All cultures were incubated at 37 °C in a humidified atmosphere containing 5% CO_2_. The medium was changed every 2 days, and cells were passaged using 0.05%/1 mM Trypsin/EDTA.

Cell viability was measured by the colorimetric MTT (3-(4,5-dimethylthiazol-2-yl)-2,5-diphenyltetrazolium bromide) test (EMD Millipore, U.S.A.). Cells (10^3^) were seeded in 100 μL medium into each well of 96-well plates and incubated for 24 h at 37 °C. The medium was then changed with fresh medium and exposed for 72 h to the drugs at the following concentrations: 1 and 10 μM Oxaliplatin (Teva Sante, France), 1 and 10 μM 5-Fluorouracil (Pfizer, U.S.A.) or 0.01 and 0.1 μM Camptothecin (Sigma, U.S.A.), in combination or not with 5 μM Lovastatin (TCI, Belgium). After the incubation periods, 10 μL of MTT reagent (5 mg/mL in PBS) were added into each well and cells were incubated at 37 °C for 3 h to allow MTT cleavage to occur. The reaction was then stopped with 100 μL isopropanol with 10% Triton X100 and 0.1 N hydrochloric acid. The absorbance was measured within 1 h, on a multiplate reader (Thermo Labsystems Multiskan spectrum, UV/Visible Microplate Reader, U.S.A.) with a test wavelength of 570 nm and a background wavelength of 690 nm.

The effects of drug treatment on transcripts levels were evaluated after 24 h of exposure to the drugs used, as mentioned above.

### Gene expression profiling by PCR array

Reverse transcription of 4 μg of total RNA was performed using the High Capacity cDNA RT kit, according to the manufacturer’s instructions (Applied Biosystems). Differential expression between CRC and NT was evaluated by real-time PCR (ABI 7000 and ABI 7300, Applied Biosystems) with the RT^2^ Profiler PCR array (Qiagen) in 96-wells plates according to the manufacturer’s instructions (Qiagen). Five types of plates (to assay expression of 84 specific genes each) were used: Apoptosis (PAHS-012A), Cancer pathways (PAHS-033A), Lipoprotein signaling and cholesterol metabolism (PAHS-080Z), Drug metabolism (PAHS-002A) and Wnt signaling pathway (PAHS-043A). Gene composition and Qiagen’s functional gene groups are indicated in Additional file 1. Nineteen genes were both present in two different PCR arrays: 14 genes in the “Apoptosis” and “Cancer pathways” arrays (*AKT1, APAF1, BAD, BAX, BCL2, BCL2L1, CASP8, CFLAR, FAS, TNF, TNFRSF10B, TNFRSF11A, TNFRSF25* and *TP53*), 2 genes in the “Cancer pathways” and “Wnt signaling pathway” arrays (*JUN* and *MYC*), 2 genes in the “Lipoprotein signaling and cholesterol metabolism” and “Drug Metabolism” arrays (*APOE* and *CYB5R3*) and 1 gene (*LRP6*) in the “Lipoprotein signaling and cholesterol metabolism” and “Wnt signaling pathway” arrays. Data analysis was performed using the ΔΔCt method [10] with normalization of the raw data to housekeeping genes. NormFinder [11] and geNorm [12] algorithms were used to determine the optimal normalization genes. A set of 4 invariant genes was used for each specific PCR array: *BIRC2, CASP8, FADD* and *TP53BP2* for the Apoptosis array; *MTA1, MTA2, PNN* and *RAF1* for the Cancer pathway array; *LDLRAP1, OSBPL5, PMVK* and *SNX17* for the Lipoprotein signaling and cholesterol metabolism array; *ADH5, ALDH, CES2* and *SMARCAL1* for the Drug metabolism array and *BTRC, CTBP1, EP300* and *PPP2CA* for the Wnt pathway array. Homogeneity of gene expression in the group of normal tissues was validated through splitting randomly the samples into two groups. Gene expression showed no significant difference in fold-changes between the two groups. Subsequently we used the mean of the pooled sample of NT for the calculation of fold-changes. Statistical analyses were performed using the R software. For each gene and each pathway, the difference in means of log_2_-transformed fold-change between NT and CRC was tested using a Student t-test. To control for multiple testing of the genes and pathways, the raw *p*-values obtained from this analysis were further adjusted with the *q*-value method. The false discovery rate (FDR)-adjusted *q*-values (FDR 5%) were calculated using the p.adjust command in R. Significance was set at q < 0.05 and fold-change limit was set at 1.7-fold. The Principal Component Analyses have been performed with R and the FactoMineR package (FactoMineR: An R Package for Multivariate Analysis, Journal of Statistical Software, March 2008, Volume 25, Issue 1).

### Protein extraction and Western blot analysis

Acetone precipitation of proteins was performed from the flow-through of the RNeasy spin column, according to the manufacturer’s instructions (Qiagen). Precipitated cytosolic proteins were suspended in 500 μL of boiling buffer (1% SDS, 1 mM Na_3_VO_4_, 10 mM Tris-HCl pH 7.4) for Western blot application. Protein quantification was carried out using the Bio-Rad DC Protein Assay system and the absorbance was measured within 1 h on a multiplate reader (Thermo Labsystems Multiskan spectrum, UV/Visible Microplate Reader, U.S.A.) at 750 nm. Forty micrograms of protein extracts were boiled in Laemmli buffer for 5 min before separation by SDS-PAGE using 8 to 12% polyacrylamide gels and transfer onto a polyvinylidene fluoride membrane (GE Healthcare, France) by electroblotting. Membranes were saturated using Sea Block Blocking Buffer (Thermo Scientific, France) for 1 h at room temperature. Primary antibodies (Bcl-2, Casp7 (Cell Signaling Technology, U.S.A.); Adh1c, Bcl2l1, Gstp1, Igf1, Nme1 (Abnova, Taïwan); Fdps (Epitomics, France); Cyp39a1, Gpi, Hmgcs1, Pcsk9, Pkm2, Hsc70 (Santa Cruz Biotechnology, U.S.A.)) were diluted in blocking buffer (Odyssey LI-COR Biosciences, U.S.A.), containing 0.1% Tween®20. Membranes were incubated with the suitable primary antibody, at 4 °C overnight, then washed four times with PBS / 0.1% Tween®20 at room temperature and incubated with the infrared absorbing secondary antibody (Odyssey®) for 1 h at room temperature. Membranes were washed again and protein bands revealed using the Odyssey®-LI-COR imaging system. Twenty paired CRC/NT samples were analyzed for each antibody. Protein expression fold-changes between 20 CRC and paired NT were evaluated by densitometry analysis, using Hsc-70 as normalization control.

### Immunohistochemistry

Formalin-fixed and paraffin-embedded samples of colorectal adenocarcinomas (*n* = 27, same patients used for Western blot analyses, Table 1) were used for the immuno-histochemical (IHC) analyses. For each case, a block containing adenocarcinoma areas and adjacent normal mucosa was selected. Immuno-histochemical techniques were performed for each case on 4 μm formalin-fixed and paraffin-embedded tissue sections mounted on Superfrost® Plus slides (Thermo Scientific, Saint-Herblain, France) dried overnight at 37 °C before processing. IHC processing was performed on a Ventana Benchmark XT® automated slide preparation system (Roche Diagnostics, Meylan, France) using the UltraView Universal DAB Detection Kit (Roche Diagnostics). The following antibodies were used: NME1 (mouse monoclonal antibody, clone 1D7, Abnova, 1:200 diluted), FDPS (rabbit monoclonal antibody, clone EPR4628, Epitomics, 1:400) and CCND1 (rabbit monoclonal antibody, clone SP4, Thermo Scientific). The slides were pre-treated with cell conditioner 1 (pH 8) for 30 min., followed by incubation with each antibody at 37 °C for 1 h. After washing, the slides were counterstained with one drop of hematoxylin for 12 min and one drop of bluing reagent for 4 min. Slides were next washed in water with dishwashing detergent and mounted. For each case, the staining intensities were compared between the adenocarcinoma glands and the adjacent normal glands. Staining of the stroma was also noted.

### Real-time polymerase chain reaction

To evaluate the impact of drug treatment on the transcript content of HCT-116 and HT-29 cells, the differential expression of selected genes was analyzed by real time PCR (StepOne plus, Applied Biosystems). Briefly, total RNA was extracted using TRIzol® reagent according to the manufacturer’s instructions (Invitrogen, France). Two micrograms of total RNA were reverse-transcribed using random hexamers and M-MLV (Moloney-murine-leukaemia virus) reverse transcriptase (New England Biolabs, France). Retro-transcribed RNA (~5 ng cDNA) was used as template for all PCR experiments (except for the 18S ribosomal RNA which was amplified from ~5 pg cDNA). PCR was performed with the Power SYBR GREEN PCR Master Mix, according to the manufacturer’s instructions (Applied Biosystems). All conditions were normalized relative to the 18S control rRNA. Primer sequences (and amplicons lengths) were: TCACTTGTGGCCCAGATAGGC and GGATGCCTTTGTGGAACTGT for BCL2 (136pb), AGGTTTAGCGCCACTCTGC and GAGCCTCAACATCCGACTCC for CHECK2 (98pb), TGCAGTGTCTCGGGACTTCG and CGCTGGTGTGGAACATCTGG for CYP11A1 (218pb), TGGAGGAATGGTATACCCTG and TCCTTTGACTGATGATGAAGTAGC for CYP51A1 (310pb), ATTTGGAGCACAGGTGTCTA and TGTCATTGGTGACGCCATCT for DHCR7(163pb), CCTGCACACCTTCCAAAACG and GTAGCAGTCGGCATACAGC for DHCR24 (236pb), TGTGGCACCAGATGTCTTCG and GCTGTGGCAGGGAGTCTTG for HMGCS1(271pb),TTCTGCAGCTCTGTGTGAAGG and TTTGGGGTGGAAAGGTTTGG for IL8 (97pb), GGAGTGGCTTTCCTGAGAAC and GGTGAGAAGCTGGCTGAGAG for ITGA2 (285pb), AAAGGATTCCGCCTTGTTGGT and GCCCTGAGTGCATGTATTTCAC for NME1 (124pb), TCCTGTATGGCTCCGAGACC and ACACCACGTCATACTCATCC for NOS3 (114pb), CTCAGAGCGGAGAAAGCATTTG and TCACCGCCTCGGCTTGTCAC for VEGFA (259pb), TAGAGGGACAAGTGGCGTTC and CGCTGAGCCAGTCAGTGTAG for 18S (104pb). The results were analyzed using the ΔΔCt method [10]. Reactions were run in three independent experiments. Statistical analyses were performed using the Student *t*-test (p<0.05).

### Data integration into biological networks

To obtain biological information about this set of deregulated genes in CRC as compared to NT, we searched for direct (physical) and indirect (functionally associated with no direct interaction) associations between 111 genes identified by transcriptome analysis in the STRING (Search Tool for the Retrieval of Interacting Genes/Proteins) database (version 10.5) [13]. Briefly, interactions in STRING are derived from multiple sources (experimental/biochemical experiments, curated databases, genomic context prediction, co-expression and automated text mining). Confidence level of edge, i.e. interaction between 2 nodes or protein/gene, is computed in a combined score (ranging from 0 to 1). Interactions and transcriptome data were imported and used for network generation in the Cytoscape environment (version 3.4) [14] focusing only on deregulated genes retrieved for each specific PCR array or on all deregulated genes. Topological analysis was performed with Network Analyzer to identify nodes parameters, including degree, clustering coefficient and betweenness centrality [15].

## Results

### RT-qPCR analysis of selected genes in colorectal tissue samples

**Fig. 1:**
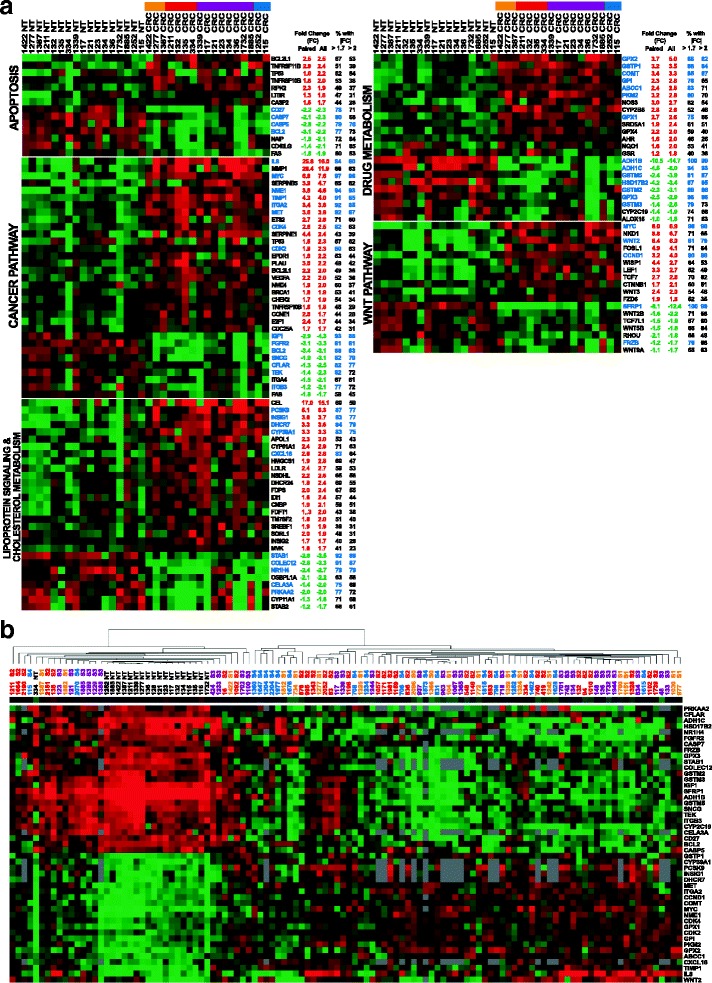
Colorectal carcinoma classification based on gene expression profiling. **a** Heat map analysis of genes deregulated in colorectal carcinoma (CRC) as compared to normal tissue (NT). Representation of the log_2_-transformed fold-change values from 111 cellular genes identified as significantly deregulated in colorectal carcinoma (CRC), compared to normal tissue (NT), with an absolute fold-change >1.7 and a q-value <0.05 (Student-t-test adjusted by FDR) using Heat Map analysis (median centered data performed in Cluster and TreeView) [63]. Deregulated genes were shown according to the type of specific PCR array and ranging from decreasing fold-change (red color for up-regulated genes and green color for down-regulated genes): Apoptosis: 14 genes, Cancer pathway: 33 genes, Lipoprotein signaling and cholesterol metabolism: 29 genes, Drug metabolism: 23 genes, Wnt pathway: 18 genes. The mean fold-change expression obtained from paired samples analysis (*n* = 17 paired CRC and NT) and all samples (*n* = 95 CRC versus *n* = 19 NT) analysis is indicated on the right of each graphic. Percentage of CRC showing an absolute fold-change superior to 1.7 and superior to 2 is also indicated on the right of each graphic. A blue color for gene names indicates a deregulation (> 1.7-fold) observed in more than 75% of tumor samples. A (−) sign and a green font refer to down-regulation; a red font refers to up-regulation. **b** Hierarchical clustering of colorectal carcinoma and normal colon tissue based on the expression profile of 47 genes. Log_2_-transformed fold-change values from 47 genes fulfilling the criteria of deregulation in more than 75% of CRC samples (fold-change >1.7 and q < 0.05) were subjected to treatment by Cluster and Treeview using uncentered correlation and average linkage [63]. Red and green colors indicate transcript levels above and below the median values, respectively. NT, normal colon tissue (*n* = 19) and CRC, colorectal carcinoma (*n* = 95). Tumor samples are identified by a number followed by the Tumor Bank running number (S0, S1, S2, S3, S4) corresponding to a grading stage according to the pathological classification. Samples names above the dendrogram were colored according to stages: orange: 0 and I, red: II, purple: III and blue: IV. Genes identified by their gene symbol appear on the right side of each panel. Each column gives the gene expression profile of a sample, and each line indicates the variations in the level of expression of a given gene among tissue samples. The length of the branches on the trees forming the dendrograms on the top of each panel reflects the degree of similarity between samples; the longer the branch, the larger the difference in gene expression

We selected five biological pathways to compare gene expression in 95 colorectal cancers (CRC) to a set of 19 normal colon tissues (NT) by RT-qPCR. These genes were classified in functional groups for each pathway (Additional file 1). We set the statistical variation threshold at a fold-change (FC) of 1.7, and a q-value of less than 0.05. From a comparison of 17 CRC to paired NT, 111 mRNAs (28% of the genes surveyed) were either up- or down-regulated in CRC samples (Fig. 1a). For each gene, we also show the % of samples with a FC of 1.7 or more (from 39% (*SREBF1*) to 100% (*ADH1B* and *SFRP1*)) and, secondly, with a FC of 2.0 or more (from 23% (*FZD6*) to 99% (*ADH1B* and *SFRP1*)). As shown in the “All” column, the data from the 17 paired samples matched well with the majority of data obtained from all samples, paired and unpaired (*n* = 95 CRC and 19 NT). There were 3 up- / 11 down-regulated genes in the Apoptosis array, 24 up- / 9 down-regulated genes in the Cancer pathway array, 21 up- / 8 down-regulated genes in the Lipoprotein signaling and cholesterol metabolism array, 14 up- / 9 down-regulated genes in the Drug metabolism array and 11 up- / 7 down-regulated genes in the Wnt pathway. The strongest increase in gene expression in CRC vs. NT was for the *IL8* gene in paired samples (25.8-fold, 16-fold for unpaired), whereas the largest decrease was −10.5-fold for *ADH1B* in paired samples (−14.7-fold for unpaired).

Hierarchical clustering analysis from each PCR array based on differentially expressed genes showed an overall clear CRC vs. NT sample separation for all but the Apoptosis pathway (Additional file 2). About 1/4 CRC did not separate out from NT samples. The majority of samples from this set behaved similarly among the five clustering analyses. They could not be discriminated according to specific stages or other clinical data (data not shown). However, hierarchical marker clustering, taking 22 up- and 25 down-regulated genes that all displayed altered (>1.7-fold) expression in at least 75% of CRC samples, we could separate most CRC out of NT samples. Only 11 out of 95 (11%) CRC samples still clustered among the NT samples (Fig. 1b). In addition, no separation was observed among cancer samples according to stage (S1 to S4). Of note, the 47 genes-based clustering was highly contributed by genes from the Lipoprotein signaling and cholesterol metabolism and (*n* = 10) and Drug metabolism (*n* = 15) pathways. These data highlight the importance of these two pathways not previously associated to CRC pathogenesis (Table 2) but showing a deregulation in the majority of CRC.

**Table 2:** Comparison between genes uncovered in this study and other cancers. Literature analysis of the genes not previously reported as deregulated in CRC, but found deregulated in other cancer types. Different expression data information was listed for each gene: i) from our study performed by PCR array technology in CRC (*n* = 95) as compared to NT (*n* = 19); ii) from literature data by focusing on expression analysis obtained in other cancers (RNA or protein level) and iii) from other literature data obtained from genetic association, epigenetic and functional studies in other cancers. Bold lines highlight newly deregulated genes in CRC and not associated to other cancers

Gene Symbol	PCR Array Data (CRC vs. NT)			Bibliography Data			
	Fold-change	*q*-value	Pathway	Type of Cancer	Expression Change (vs. NT)	Ref.	Other data
CEL	15.08	< 0.001	Chol. Met.	Pancreas	↓ (RNA)	[25]	/
				Nasopharynx	↑ (RNA)	[24]	/
PCSK9	6.28	< 0.001	Chol. Met.	Lung	↓ (RNA)	[23]	PCSK9 deficiency reduces liver metastasis [64].
INSIG1	3.69	< 0.001	Chol. Met.	Breast	↑ (protein)	[65]	/
DHCR7	3.56	< 0.001	Chol. Met.	/	**/**	**/**	**/**
CYP39A1	3.35	< 0.001	Chol. Met.	Cholangiosarcoma	↓ (protein)	[66]	/
APOL1	2.97	< 0.001	Chol. Met.	/	**/**	**/**	**/**
CYP51A1	2.95	< 0.001	Chol. Met.	/	**/**	**/**	**/**
HMGCS1	2.79	< 0.001	Chol. Met.	/	**/**	**/**	Non-synonymous mutation associated to hepatocellular carcinoma [67].
NSDHL	2.61	< 0.001	Chol. Met.	/	**/**	**/**	**/**
CYP2B6	2.61	< 0.05	Drug Met.	Breast	↑ (RNA)	[68]	**/**
				Hepatocellular	↑ (RNA)	[69]	
DHCR24	2.44	< 0.01	Chol. Met.	Adrenals	↓ (RNA)	[70]	/
				Prostate	↑ low risk and ↓ advanced (protein)	[71]	
FDPS	2.40	< 0.001	Chol. Met.	Prostate	↑ (protein, RNA)	[72, 73]	/
SRD5A1	2.37	< 0.001	Drug Met.	Non-small cell lung cancer	↑ (RNA)	[74]	/
				Prostate	↑ (RNA, protein)	[75]	Associated with biological aggressiveness in prostate cancer [76].
				Breast	↑ (RNA)	[77]	/
IDI1	2.37	< 0.001	Chol. Met.	/	**/**	**/**	Altered expression in response to paclitaxel treatment in ovarian carcinoma (nude mice xenografts) [78].
EPDR1	2.25	< 0.01	Cancer	/	/	/	/
CNBP	2.09	< 0.001	Chol. Met.	/	**/**	**/**	**/**
FDFT1	2.01	< 0.01	Chol. Met.	Gastric	↑ (RNA, protein)	[79]	The A allele of rs2645429 (promoter) was significantly associated with prostate cancer risk in a Japanese familial population (~increase promoter activity) [80].
				Prostate	↑ (RNA)	[80]	
TM7SF2	1.96	< 0.001	Chol. Met.	Adrenocortical tumors	↓ (RNA)	[81]	**/**
				Follicular thyroid carcinoma	↑ aggressive vs. non-aggressive (RNA)	[82]	
RIPK2	1.95	< 0.001	Apoptosis	Breast	↑ (RNA)	[83]	KO of RIPK2 in murine model of bladder Cancer induces large tumors and higher incidence of metastases [84].
				Oral squamous cell carcinoma	↓ (protein)	[85]	
SREBF1	1.93	< 0.001	Chol. Met.	Hepatocellular	↑ (RNA, protein)	[86]	/
SORL1	1.90	< 0.01	Chol. Met.	Astrocytoma	↑ (RNA)	[87]	/
GSR	1.82	< 0.05	Drug Met.	/	/	/	/
MVK	1.72	< 0.001	Chol. Met.	/	/	/	/
GSTM5	−3.92	< 0.001	Drug Met.	Barett’s esophagus	↓ (RNA)	[27]	Associated to DNA hypermethylation in Barett’s adenocarcinoma [27], in human salivary gland adenoid cystic carcinoma [88], in myelodysplasic syndrome [89], in glioblastoma [26].
				Glioblastoma		[26]	
COLEC12	−3.32	< 0.001	Chol. Met.	Anaplastic thyroid carcinoma	↓ (RNA)	[90]	/
GSTM2	−3.10	< 0.001	Drug Met.	Barett’s esophagus	↓ (RNA)	[27]	Associated to DNA hypermethylation [27, 91].
				Oral squamous cell carcinoma		[91]	
GSTM3	−2.49	< 0.001	Drug Met.	Barett’s esophagus	↓ (RNA)	[27]	Associated to DNA hypermethylation in Barett’s adenocarcinoma [27].
				Lung		[92]	
CD27	−2.30	< 0.001	Apoptosis	Bladder	↓ (RNA)	[93]	/
				B Cell Lymphoma	↑ (protein)	[94]	
				CLL	↑ (RNA)	[95]	
TEK	−2.30	< 0.001	Cancer	Non-small cell lung cancer	↓ (RNA)	[96]	/
				Angiosarcoma	↑ (RNA)	[97]	
				AML and CML		[98]	
				Thyroid	↑ (RNA, protein)	[99]	
				Breast	↑ (protein)	[100]	
RHOU	−1.78	< 0.001	Wnt	Prostate	↓ (RNA)	[101]	/
WNT2B	−2.19	< 0.001	Wnt	Pancreas	↑ (protein)	[102]	/
				Stomach	↑ (RNA)	[103]	
				Basal carcinoma (skin)	↑ (RNA)	[104]	
ITGA4	−2.13	< 0.001	Cancer	CLL	↑ (RNA, protein)	[105]	/
ITGB3	−2.11	< 0.001	Cancer	Liver	↓ (RNA, protein)	[106]	/
				Ovary	↑ (RNA, protein)	[107]	
CD40LG	−2.09	< 0.001	Apoptosis	Inflammatory breast cancer	↑ (RNA)	[108]	/
CELA3A	−2.04	< 0.001	Chol. Met.	Mucinous pancreatic cyst	↑ (protein) vs non mucinous	[109]	/
PRKAA2	−1.96	< 0.001	Chol. Met.	Ovary	↑ (RNA)	[110]	/
CYP2C19	−1.94	< 0.001	Drug Met.	Breast	↓ (protein)	[68]	/
				Liver	↑ (RNA)	[111]	
CYP11A1	−1.85	< 0.001	Chol. Met.	Prostate	↓ (RNA)	[112]	Associated to DNA hypermethylation in prostate cancer [112].
				Endometrium		[113]	
WNT5B	−1.83	< 0.001	Wnt	CLL	↑ (RNA)	[114, 115]	/
				Uterine leiomyoma		[116]	
FRZB	−1.73	< 0.01	Wnt	Liver	↓ (RNA)	[117]	Associated to DNA hypermethylation in hepatocellular carcinoma [117], medulloblastoma [118] and bladder cancer [119].
				Melanoma		[120]	
				Medulloblastoma		[118]	
				Bladder		[119]	
				Gastric		[121]	
				Breast		[122]	
WNT9A	−1.68	< 0.001	Wnt	CLL	↑ (RNA)	[114, 115]	/
STAB2	−1.68	< 0.001	Chol. Met.	Liver	↓ (protein)	[123]	/

*Abbreviations*: *Chol. Met*. Lipoprotein signaling and cholesterol metabolism, *Drug Met* Drug metabolism, *Wnt* Wnt signaling, Cancer: Cancer pathway; *CLL* Chronic lymphocytic leukemia, *AML* Acute myeloid leukemia, *CML* Chronic myeloid leukemia

Quite expectedly, several of our observed gene expression changes agreed with former reports, including a previous microarray study that we recently reported [16]. For all those mRNAs that were detected by both PCR arrays and microarrays, there was a 100% concordance in the up- or down-regulations observed (28 / 28 genes in CRC and 41 / 41 genes in colorectal adenomas). However, there were a few discrepancies between our studies and some other reported data (Additional file 3). For instance, BCL2 mRNA and protein levels were decreased in our samples (−2.18-fold in the PCR array, −2.71-fold in microarray in CRA, −5.62-fold in microarray in CRC and −4.76 in TCGA study [17]), but increased in the study of Sun et al. [18]. NOS3 mRNA was increased in our study and in that of Yagihashi et al. [19], but decreased in the study of Yu et al. [20] and showed no change in TCGA study [17]. GPX1 mRNA was increased in our study and in that of Yagublu et al. [21] and a small increase in TCGA study [17], but decreased in that of Nalkiran et al. [22].

**Fig. 2:**
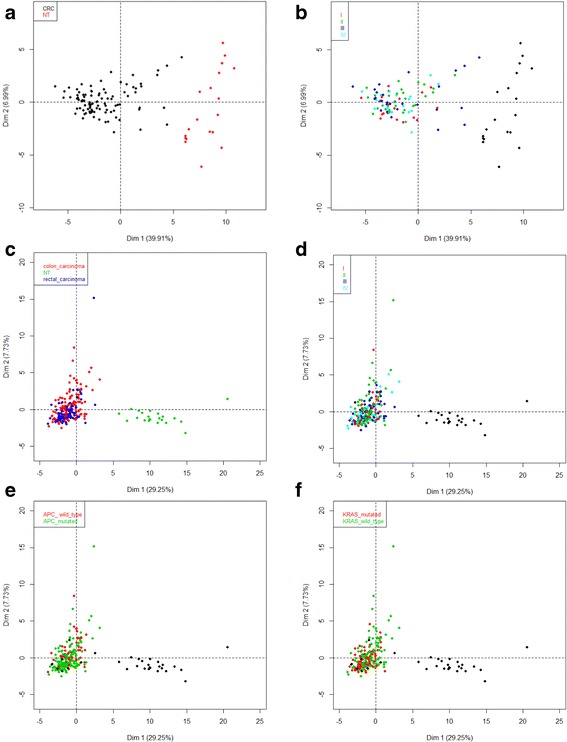
Principal Component Analysis of two independent datasets of colorectal carcinoma based on the expression of 47 genes. Principal component analysis (PCA) were performed based on the expression in normal tissue and colorectal carcinoma of 47 genes selected by the criteria of deregulation in more than 75% of CRC samples (fold-change >1.7 and *q*-value <0.05). PCA was performed on our samples dataset (19 NT and 95 CRC) (panels **a** and **b**) and on expression data retrieved from the colorectal cancer cohort from TCGA (gdac.broadinstitute.org) composed the TCGA tumor dataset. Repartition of grading stage of TCGA colorectal carcinoma was: I: 47, II: 86, III: 54 and IV: 34. Fifty-eight tumors were APC wild-type and 153 were mutated. One hundred and twenty-six were KRAS wild-type and 85 mutated. Individuals were colored according to a category variable in each PCA plot: i) according to histological type (panels **a** and **c**); ii) according to grading stage (panels **b** and **d**); iii) and, only for the TCGA PCA plot, according to the mutational status of APC (panel **e**) and KRAS (panel **f**). Samples corresponding to NT and tumors that presented no available information about categorical variables were colored in black for panels **b**, **d**, **e**, **f**

To confront our data to other available data sets, we used these same genes to test for the correct separation of NT and CRC using the colorectal carcinoma cohort (COADREAD) from The Cancer Genome Atlas (TCGA) dataset (gdac.broadinstitute.org). We performed a Principal Component Analysis (PCA) using expression data for 47 genes selected in our study (fold-change >1.7, retrieved in more than 75% of CRC samples). Similarly to our hierarchical clustering (Fig. 1b), the PCA clearly distinguished two groups: NT and CRC, thereby validating the expression data from our sample (Fig. 2). Furthermore, based on expression of the same 47 genes, colon and rectum carcinoma were undistinguishable on PCA plots. A similar observation was made from PCA performed with other datasets (sets of deregulated genes related to functional Qiagen classification and all of the 111 deregulated genes identified with our samples) (Additional file 4). Finally, the colorectal tumors could not be separated according to the grading stage or the APC and KRAS mutational status of (Fig. 2, Additional file 4), indicating that the deregulated gene expression of genes identified with our samples was independent of tumor grades and of APC and KRAS mutations.

A high proportion of the deregulated genes found here have not been reported in CRC, although the very large majority had been described in other cancers, mostly solid cancers, at the mRNA and/or protein levels (Table 2). Not surprisingly, the most deregulated genes had been identified in CRC in previous studies, whereas less deregulated genes were new. For instance, *PCSK9* (6.3-fold increase), *CEL* (15.1-fold increase), or *GSTM5* (3.9-fold decrease), despite their relatively important deregulation, had only been reported in lung cancer for *PCSK9* [23], pancreatic and nasopharyngeal carcinoma for *CEL* [24, 25], Barret esophagus and glioblastoma for *GSTM5* [26, 27]. Some of the deregulated genes had no known deregulation linked with cancer, including several genes that belonged to the Lipoprotein signaling and cholesterol metabolism pathway (*DHCR7, CYP51A1, NSDHL, HMGCS1, IDI1, CNBP, APOL1, SREBF1*), and a few from other pathways, like *RHOU* (Wnt signaling pathway) and *EPDR1* (Cancer pathway).

To analyze the repercussion of these gene expression changes at the protein level, we selected a set of genes deregulated in more than of 75% of CRC (Fig. 1d). Western Blot analysis was conducted for 9 up-regulated (*BCL2L1*, *CYP39A1, FDPS, GPI, GSTP1, HMGCS1, NME1, PCSK9* and *PKM2*) and 4 down-regulated genes (*ADH1C, BCL2, CASP7* and *IGF1*). The majority of changes in mRNA (19 or 20 out of 20 analyzed CRC samples) levels were associated with comparable changes in protein levels, with the exception of *PCSK9*, which showed a large mRNA increase but a small and not significant drop at the protein level, and for GPI, which remained essentially unchanged in the majority of CRC (17 out 20) (Additional file 5: Figure S3). As expected, with respect to transcript levels, these changes occurred regardless of cancer stage, for both up- and down-regulated genes. In addition, BCL2L1 mRNA up-regulation, which occurred in 63% of CRC, was associated with a parallel increase in protein level. Immunohistochemistry analysis of NME1 and FDPS proteins conducted for 27 paired NT/CRC samples confirmed the deregulation observed in Western blot analyses (Additional file 6). CCND1, a Wnt signaling target gene that showed mRNA up-regulation in 82% of CRC, was also increased in CRC vs. NT in IHC analyses.

### In silico protein pathways analyses

**Fig. 3:**
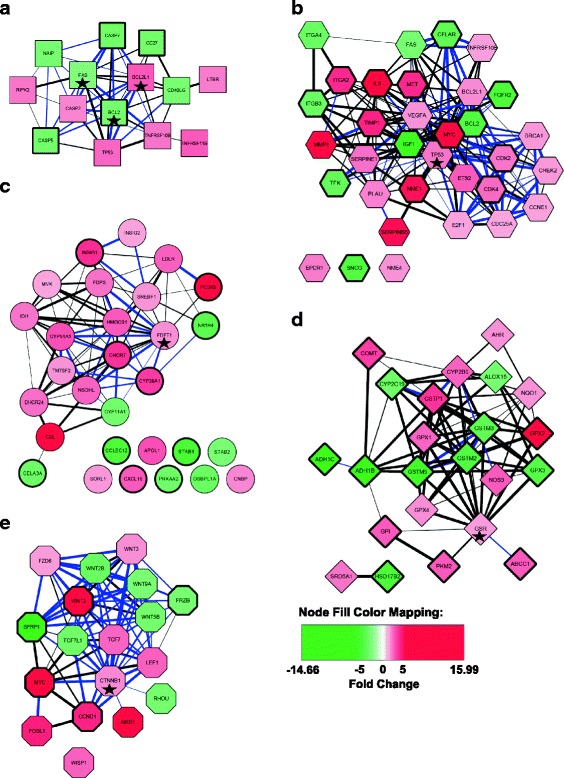
Network building from deregulated genes in different types of PCR arrays. Interactions between sets of deregulated genes identified by transcriptome comparison of CRC and NT were retrieved from the STRING database (version 10.5) for 5 different PCR arrays: **a**) Apoptosis, **b**) Cancer pathway, **c**) Lipoprotein signaling and cholesterol metabolism, **d**) Drug metabolism and **e**) Wnt pathway. The interactions include direct (physical) and indirect (functional) associations. All interaction sources questioned by STRING were used and the minimum required interaction score was 0.4 (medium confidence). The blue color of edges indicated a physical interaction between 2 nodes. The thickness of edges was correlated with score confidence (large for high score). Color intensity of nodes was related to transcriptome deregulation (red for up-regulation and green for down-regulation in CRC as compared to NT). Genes showing a deregulation in more than 75% of CRC were characterized by a bold line of shape node. Star symbols indicated the node showing the highest number of interactions in the network

In view of the overall good correlation between mRNA and protein expression changes, we went on to build up presumptive protein-protein interaction maps. We used the STRING database and the Cytoscape environment to integrate interactions data into biological networks (Fig. 3) [13, 14]. STRING allows recognition of both demonstrated protein-protein interactions (PPI) or members of canonical pathways, predicted associations based on genomic context or co-expression and literature text mining. Symbols with large lines around proteins (nodes) reflect the genes showing mRNA deregulation in more than 75% of CRC, as compared to NT, and the colors indicate the level of up- and down-regulation by a color gradient (pink to dark red for up-regulated genes and light to dark green for down-regulated genes). Thick lines between proteins indicate interactions (edges) associated with a stronger probability (confidence score ranging between 0.4 and 1) and blue lines represent an experimentally validated physical PPI from the source of evidence for the considered edges.

Taken individually, each network derived from the corresponding PCR array showed many functional associations (Fig. 3). Some proteins showed a prominent position in each pathway, characterized by a high number of degrees, i.e. interactions (also named “molecular hubs”), like BCL2, BCL2L1 and FAS (Apoptosis pathway), TP53 (Cancer pathway), FDFT1 (Lipoprotein signaling and cholesterol metabolism pathway), GSR (Drug metabolism pathway) and CTNNB1 (Wnt pathway). The Cancer and the Wnt pathways showed the highest density of interactions (33 nodes and 174 edges, and 18 nodes and 75 edges, respectively), while the apoptosis pathway showed the least interactions (14 nodes and 45 edges) (Fig. 3). Fusion of these 5 individual networks associated to each functional category (from each PCR array) led to a global network (111 nodes and 590 edges) (Additional file 7: Figure S5A). Thirty-nine percent of interactions included a physical PPI and 33.5% included components of canonical pathways. All 5 pathways showed many links with one another, with only very few proteins (8 out of 111, 7.2%) not apparently involved in any interaction with the other proteins. The original affiliations to each functional group were maintained with the Wnt pathway group at the top right, the Lipoprotein signaling and cholesterol metabolism to the bottom left, the Drug metabolism to the top left and mixed Apoptosis and Cancer groups to the center of the network. However, we also observed an association between these groups through connecting nodes (NME1, CTNNB1, CYP51A1, ABCC1, CYP2B6, NOS3, INSIG2, PCSK9, SREBF1, LDLR, NQO1 …).

To gain some insight into biological processes and identify key nodes in the global network, we performed a topological analysis with NetworkAnalyzer, a Cytoscape plugin [15]. The top-5 proteins with higher interactions, which are considered as molecular hubs, were TP53, MYC, CTTNB1, BCL2 and VEGFA (Additional file 7: Figure S5B) Fifty-two percent of the proteins had more than 10 interaction partners and 9% had more than 20 partners. These hub proteins were more important than poorly connected proteins (less than 11 partners, i.e. the median degree of nodes) and were relevant for colorectal cancer pathogenesis. To better identify important nodes, we looked at the clustering coefficient that refers to the tendency of node’s neighbors to connect to each other (Additional file 8: Figure S6A). Since many cellular processes are governed by subsets of components that form an interaction module, it is expected that nodes with high clustering coefficients should be biologically relevant. Interestingly, we could identify a group of 32 proteins, corresponding in majority to members of the Cancer pathway but also the Wnt pathway, which combined a higher degree and a higher clustering coefficient (Additional file 8: Figure S6B). To a lesser extent, we could note the presence of IL8, TIMP1 and MMP1 (Cancer group) that shared these characteristics.

A second set of proteins (*n* = 25), characterized by a high degree and a low clustering coefficient (inferior to the threshold, i.e. the median), retained our attention since this group concentrated: i) the hubs of the network (nodes with degree superior to 20) and ii) the top-10 proteins with highest betweenness centrality (TP53, CTNNB1, MYC, IGF1, CYP2B6, VEGFA, IL8, BCL2, LDLR and GSTP1), which measures the number of shortest paths going through a certain node. Therefore, nodes with the highest betweenness control most of the information flow in the network, representing the critical points of the network, or “bottlenecks”. Hub-bottleneck nodes are presumably most important for network organization, because their elimination leads to network disruption and, from a biological viewpoint, can abrogate the flow of information. In addition, many hubs qualified as hub-bottleneck nodes, like TP53, CTNNB1, MYC, IGF1, VEGFA, IL8, BCL2, but others, like CYP2B6, LDLR, GSTP1, should be viewed as hub-non-bottleneck nodes.

**Fig. 4:**
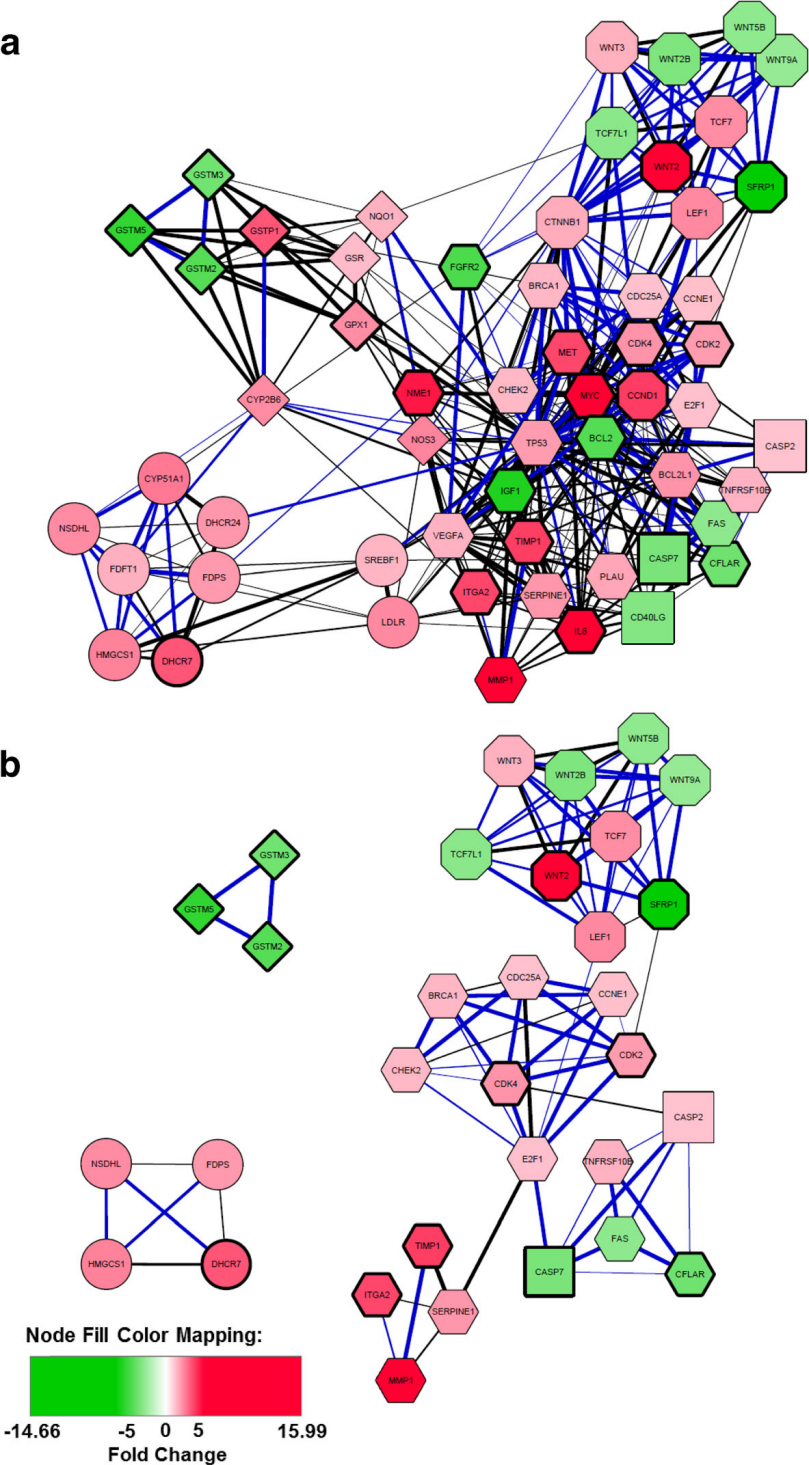
Network based on a restricted number of genes after selection of topological parameters. **a** Network based on 57 genes showing a node degree superior to 11 (median of node degree of 111 genes-based network). **b** Network based on 32 genes showing a node degree superior to 11 and a clustering coefficient superior to 0.55 (median of clustering coefficient of 111 genes-based network). Blue color of edges indicated a physical interaction between 2 nodes. The thickness of edges was correlated with the score confidence (large for a high score). The node shape illustrated the specific PCR array: Apoptosis: square, Cancer Pathway: hexagon, Lipoprotein signaling and cholesterol metabolism: circles, Drug metabolism: diamond and Wnt pathway: octagon. The color intensity of nodes was related to transcriptome deregulation (red for up-regulation and green for down-regulation in CRC as compared to NT). Genes showing a deregulation in more than 75% of CRC were characterized by bold line of shape node

Finally, we selected 57 proteins, out of the 111 proteins from the global network (Additional file 7: Figure S5A), with a median degree above 11 (appearing to the right of the vertical dotted line, Additional file 8: Figure S6B), to build a new network (Fig. 4a). This network combined the proteins implicated in formation of topological modules i.e. with a locally dense neighborhood in the initial network (Fig. 4b). It also included other important proteins, molecular hubs, behaving or not as “bottlenecks”. Reduction of the initial network to these particular nodes highlighted the Wnt signaling, Lipoprotein signaling and cholesterol metabolism and Drug metabolism modules, characterized by a high level of interaction between these members and indirect relations with other components of the network through hub-bottleneck nodes. They could offer two angles of attack by therapeutic drugs. In the same way, targeting central nodes (as IL8, CHECK2, NME1, SFRP1, FGFR2, NOS3, CYPB6, SREBF1 etc.) could influence all partners belonging to Wnt signaling, Lipoprotein signaling and cholesterol metabolism or Drug metabolism modules. Hence, components of reduced networks (Fig. 4b) could be proposed as future targets by new chemotherapeutic strategies.

#### Effect of drugs on selected gene expression

We have previously demonstrated the potential beneficial effects of Lovastatin, a competitive inhibitor of the 3-Hydroxy-Methyl-Glutaryl-CoA reductase (HMG-CoA reductase), the first enzyme from the cholesterol synthesis pathway, in gastric cancer. Indeed, although Lovastatin is not an anticancer agent, it triggered a high degree of apoptosis of the gastric carcinoma HGT-1 cell line, an effect linked to the shortage of intermediary metabolites from the cholesterol synthesis pathway [28]. In addition, we showed that a combination of Lovastatin and Docetaxel had a synergistic effect on induction of HGT-1 cells’ apoptosis. Here, Lovastatin (5 μM, 72 h) strongly reduced HCT116 cells’ viability. A stronger effect was observed for the combination of Lovastatin and 5-Fluorouracile or Camptothecin, even for lower concentrations of the drugs (Additional file 9: Figure S7A). However, the impact of Lovastatin on HT-29 viability was small (Additional file 9: Figure S7B) but it became quite clear when a higher concentration was used (12.5 μM; data not shown).

In order to see if any of the gene deregulations could be counteracted by these drugs, we looked at their effects on expression of a subset of genes in HCT116 and/or HT29 human colon cancer cells, according to their network behavior: hub-bottleneck genes such as *BCL2, ITGA2, NOS3* and *HMGCS1*, hubs as *VEGFA*, high clustering coefficient genes like *CHECK2* and *IL8* and connector’s nodes i.e. genes showing edges between functional groups, like *NME1, CYP51A1, CYP11A1* and *DHCR7*. Gene expression levels were analyzed after 24 h of treatment, i.e. before appearance of apoptotic features, by Oxaliplatin, 5-Fluorouracile or Camptothecin, either alone or combined with Lovastatin (Table 3). We observed that Oxaliplatin amplified the effects seen in CRC for most genes in both cell lines. 5-FU amplified the variation seen in CRC (for *CHECK2* in HT-29, for *NOS3* in HT-29 and HT-116), but opposed it (*CHECK2* and *CYP51A1* in HCT-116, *VEGFA* in HT-29, *HMGCS1* in both cell lines). Camptothecin amplified the effects seen in cancer (*CHECK2, BCL-2* and *IL8*), but opposed them for *CYP51A1, ITGA2, DHCR7* or HMGCS1. Lovastatin had either no effect (in HCT116 cells), or opposed the deregulation of the *CHECK2, NME1, ITGA2, IL8, NOS3* or *VEGFA* (in HT-29 cells). Lovastatin mixed with Oxaliplatin opposed the effect seen upon treatment by Oxaliplatin alone for the *NME1* and *ITGA2* genes in HCT-116 cells. The combination of Lovastatin and 5-Fluorouracile had a quite distinct effect from 5-Fluoruracile alone, especially for the *NME1, ITGA2* or *HMGCS1* genes that showed opposite regulations. The association of Lovastatin and Camptothecin essentially did not modify the response to Camptothecin, except for the NME1 gene that showed decreased expression in response to the combination of treatments.

**Table 3:** Changes in mRNA levels in human CRC cell lines treated by different chemotherapeutic drugs and/or by Lovastatin. After 24 h of treatment by different chemotherapeutic drugs (Oxaliplatin (10 μM), 5-Fluorouracile (10 μM), Camptothecin (0.1 μM) in combination or not with Lovastatin (5 μM), total RNA was extracted and submitted to reverse transcription. Real-time PCR for different genes was performed. Three independent experiments were analyzed. Fold-change was indicated when the *p*-value was significant Student’s-t test)

Gene Symbol	Fold-change CRC vs. NT	CRC line	Oxa	5-FU	Cpt	Lova	Oxa + Lova	5-FU + Lova	Cpt + Lova
CHECK2	2.08***	HCT-116	NC	0.69*	NC	NC	0.56***	0.44**	NC
		HT-29	2.74*	2.43	1.40*	0.51*	2.60*	1.84*	2.33**
CYP51A1	2.80***	HCT-116	NC	0.44*	0.31*	NC	0.56**	0.22**	(0.64)
CYP11A1	0.32**	HCT-116	NC	NC	NC	NC	0.53*	0.78**	NC
BCL2	0.31***	HCT-116	0.23***	0.46*	0.52*	NC	0.14***	0.17***	0.61**
NME1	4.6***	HCT-116	2.72***	NC	NC	NC	0.60**	0.30**	0.52*
		HT-29	1.81*	NC	NC	0.52*	NC	(0.61)	NC
ITGA2	3.8***	HCT-116	1.77**	NC	NC	NC	0.68*	0.35*	NC
		HT-29	NC	NC	(0.41)	0.38*	0.50**	(0.51)	0.47**
IL8	8.1**	HCT-116	3.35***	NC	NC	NC	(3.87)	3.37*	(3.01)
		HT-29	2.92*	4.74*	2.33*	0.64***	3.24*	3.81*	3.52***
NOS3	2.3***	HCT-116	1.87**	1.63***	NC	NC	NC	2.13*	NC
		HT-29	NC	3.42**	NC	0.56**	NC	1.97*	(2.16)
DHCR7	3.6***	HCT-116	NC	NC	0.48*	NC	NC	NC	0.49**
		HT-29	NC	NC	NC	NC	1.50*	2.29***	NC
HMGCS1	2.46***	HCT-116	0.82*	0.46**	0.23***	NC	NC	NC	0.32*
		HT-29	NC	0.37**	0.42*	NC	NC	NC	NC
VEGFA	2.04***	HT-29	NC	NC	NC	0.43**	NC	0.54*	NC

*Abbreviations*: *Oxa* oxaliplatin, *5-FU* 5-Fluorouracile, *Cpt* Camptothecin, *Lova* Lovastatin

*NC* no change; * *p* < 0.05; ** *p* < 0.01; *** *p* < 0.001

Numbers in parentheses were not significant on three independent experiments, although a tendency to variation was recorded in two out of three experiments

Finally, we searched for potential associations between the four drugs and deregulated gene products in CRC by interrogating the STITCH database [29]. Interestingly, many links were identified with genes having a high degree or clustering coefficient, predicting a high impact on the network in the case of combination of Lovastatin and anticancer drugs (Additional file 10).

## Discussion

In the present study, we analyzed expression of a subset of protein-encoding genes in human colon cancers, relative to that of healthy colon tissue. All tissue fragments were retrieved from surgical pieces from patients not taking anticancer drugs. Surprisingly, although we found already described alterations, about 10% of the changes had occurred in genes with no reported link with colon cancer. We identified 111 genes (28%), among a total set of 401 tested genes, which were deregulated in cancer samples (FC > 1.7, q-value < 0.05). Although, as expected, several of these gene deregulations occurring in CRC had already been reported, 48 (12%) had no known links with this cancer. All pathways showed clear clustering distinctions between CRC and NT, with the exception of the Apoptosis pathway, for which the FC and the number of deregulated genes were lower than for the other pathways. Although all changes were statistically significant, the proportion of genes with modified expression in at least 75% of the CRC samples ranged from 1/5 (Wnt pathway) up to 1/2 (Lipoprotein signaling and cholesterol metabolism, Drug metabolism pathways). Our clustering and PCA could not separate CRC samples according to stage (from I to IV), neither at the mRNA, nor at the protein levels, possibly due to the limited numbers of samples from each pathological category, or from the target genes surveyed (Figs. 1b and 2b). However, the same analyses conducted on an independent cohort (retrieved to TCGA) containing more samples for each grading stage (> 30 per category), have failed to discriminate CRC according to stage (Fig. 2d). Furthermore, our PCA performed with the TCGA cohort, based on expression data from different gene sets (all deregulated genes or subsets of genes from the Qiagen PCR Arrays) did not allow discriminating colon vs. rectal tumors or CRC samples according to APC or KRAS mutational status (Fig. 2, Additional file 4). Importantly, based on a set of 13 genes analyzed in 20 CRC/NT sample pairs, the same regulation occurred at both the mRNA and protein levels in at least 17 samples (roughly 90% concordance with mRNA levels), except for the PCSK9 protein, which showed no consistent protein expression pattern, or the GPI protein that remained essentially unchanged (Additional file 5: Figure S3C).

In view of the overall good correlation between mRNA and protein expression changes, we went on to build up putative protein-protein interaction maps, using the STRING interaction database. Looking at the individual PCR-based pathways, it appeared that almost all cancer proteins were densely connected. The MYC and the IL8 proteins were the most up-regulated proteins and the IGF1 and FGFR2 proteins the most down-regulated, in at least 75% of the CRC samples. These observations agreed with previous reports [30–36] and further emphasized the major roles played by these proteins in the control of CRC development. The SNCG protein was not interacting with any of the other proteins from the pathway, yet its down-regulation occurred in more than 75% of the CRC samples. Increased expression of the *SNGG* gene was suggested to be a predictor of poor prognosis in esophageal or endometrial cancer patients [37, 38], which is distinct from the negative regulation observed here. *SFRP1*, from the Wnt pathway, was strongly down-regulated in all CRC samples of this study, like most of the genes from the pathway, as opposed to the *WNT2* and the *CCND1* genes, which were increased in more than 75% of the CRC samples, agreeing with previous results [39, 40]. The number of PPI was also the highest for this network. The *ADH1B* and the *GPX2* genes, from the Drug metabolism pathway, were the most down- and up-regulated, respectively, in more than 75% of the samples. Although the up-regulation of *GPX2* by the Wnt pathway had been reported [41], this is the first instance of a deregulation in cancer for ADH1B mRNA. Similarly, no report was available for the regulation of ADH1C mRNA in cancer, and it showed no integration into our network except a physical interaction with ADH1B [42]. A recent publication identified ADH1B and XYLT1 (an enzyme implicated in the biosynthesis of glycosaminoglycan chains) as interaction partners of ADH1C using high-throughput affinity purification [42]. Furthermore, XYLT1 is an interaction partner of MYC [43], the second most up-regulated gene in CRC and the second top molecular hub identified in our network. *MYC* down-regulated *ADH1B* and *ADH1C,* pointing to a contribution of these detoxification enzymes in CRC pathogenesis. The Lipoprotein signaling and cholesterol metabolism pathway showed the highest number of proteins not interacting with any other protein. Notably, two of the most important changes occurred for PCSK9 (up-regulation) and NR1H4 (FXR) (down-regulation) mRNAs, both linked to the LDLR protein. Again, this is the first report of a clear link of PCSK9 up-regulation in colon cancer, while NR1H4 down-regulation has been reported to result from promoter methylation with this cancer [44]. Several of the unlinked proteins, including COLEC12 and STAB1, showed changes in at least 75% of the CRC samples. No report was available for the COLEC12 transcript, whereas PRKAA2 was shown to promote colon cancer [45]. STAB1 was increased in tumor infiltrating macrophages [46, 47]. Finally, a small number of affected proteins belonged in the Apoptosis pathway, with the down-regulation occurring in more than 75% of the CRC samples, including the CASP7, CASP5, the BCL2 and the CD27 proteins. Although CASP7 was decreased in colon cancer as a consequence of gene deletion, no data was available for the *CASP5* or *CD27* genes, while *BCL2* was over-expressed in cancer tissue [18].

An expression change in any protein connected to another one, from either the same network or from a separate network, is expected to alter the functional links both with the immediate partner and, by association, with other activities of the network or of associated networks. Hence, one way to compensate for alterations occurring in one of these networks altered in CRC, could be to restore network function by mimicking the effect of the interactions, by drugs for instance. Despite the fact that the individual networks were quite identifiable by their position within the global network, strong links between the sub-networks - often with a high degree of confidence - could be readily distinguished. Importantly, among the main hub genes identified through the whole transcriptome-based approach [6], the *IL8* gene was also sorted out here, suggesting that this gene is indeed a master gene strongly deregulated in CRC. None of the other top 10 hub genes from the global approach was surveyed in our PCR approach. However, the major hub genes identified here, such as *TP53*, *MYC* or *BCL2*, were not picked up by the global approach. However, the authors pointed to an enrichment of drug metabolism pathway from their down-regulated genes [6], confirming our results and further stressing the importance of components of this pathway in CRC pathogenesis.

Unexpected links were identified using different sources in the STRING database. For example, a physical interaction, indexed in the BioGRID database [48], was identified between NME1, which possesses geranyl and farnesyl pyrophosphate kinase activities, and FDPS, a key enzyme in isoprenoid biosynthesis, which catalyzes the formation of farnesyl diphosphate [49]. The up-regulation of transcript and protein levels in CRC highlights the importance of this complex for synthesizing secondary metabolites (geranyl and farnesyl pyrophosphates) in pathogenesis. Acting on the mevalonate pathway to reduce secondary metabolites and ultimately counteract the activities of FDPS and NME1 looks attracting as a novel cancer therapy, in agreement with several data [50]. Because NME1 transcript levels were decreased in colon cancer cells upon Lovastatin treatment (Table 3), it is tempting to speculate that acting on this complex might permit counteracting other members of the cholesterol metabolism pathway that act downstream of NME1, together with key cancer nodes identified by topology network analysis (MYC, CCND1, BCL2…) linked to NME1. Expectedly, blunting the mevalonate pathway in cancer cells would lead to apoptosis [28].

Another example of connection between 2 functional groups is the association, retrieved by text mining, between *MYC* and *SREBF1* (Cancer and Lipid signaling and cholesterol metabolism, respectively). The authors showed that the SREBP-1 protein interacted with c-myc and facilitated its binding to downstream targets. This promoted mouse somatic cell reprogramming by expression of pluripotent genes [51]. In addition, we also identified the *CASP7* (Apoptosis) gene as a positive target of the SREBP-1 protein [52].

NQO1, an NADPH dehydrogenase implicated in the detoxification system, has been shown to directly interact with NME1 and TP53 (Cancer Pathway) in studies retrieved from the BioGrid database [48]. Furthermore, NQO1 stabilized TP53 and partially inhibited its degradation under conditions of oxidative stress, making it a possible contributor to tumor development [53].

Putative homologs of CHECK2 and INSIG1 from *Saccharomyces cerevisiae*, of DHCR7 and CYP51A1 and of CYP2B6 and NSDHL were found to have physical interactions in Intact and BioGRID databases, which may explain the relations observed in our network [48, 54]. Putative homologs of CYP2B6 and NR1H4 in *Caenorhabditis elegans* were also found in these databases. Further studies will be required to understand this relation.

Visualization of our 113 genes-network, on the basis of two topological parameters, the degree and the clustering coefficient, highlighted 4 modules related to functional gene groups characterized by different levels of clustering coefficient and node degree (Additional file 8: Figure S6A). Because chemotherapy is commonly used to treat colon cancer patients, we looked at the activity of prototypic anticancer drugs and/or Lovastatin on expression of deregulated genes occupying central places within the networks. At first sight, a drug or drug combination that would oppose the observed deregulation of such “major genes” would be useful, provided these genes are central to normal tissue homeostasis. Conversely, drugs that would enhance the deregulation effects might be inappropriate, if the given genes are important for cancer initiation or progression, according to their expression level at baseline. The *IL8* gene, which was clearly over-expressed in CRC, was further up-regulated in HT29 cells in response to all drugs or drug combinations, except in response to Lovastatin that decreased its expression. In the case of this important inflammatory mediator, it would appear that none of the common anticancer drugs used would contribute to inflammation reduction, at least at the colon cell level. Similarly, *CHECK2* was always induced (HT29 cells), except in response to Lovastatin that decreased it. In this case, however, it might be beneficial to treat CRC patients bearing tumors addicted to *CHECK2* for growth with common CRC anticancer drugs and *CHECK2* inhibitors [55].

Several actions could be taken to reestablish normal transcript levels through using selected drugs: i) targeting proteins with high numbers of interactions (hubs proteins: large size on Additional file 8: Figure S6) or ii) targeting one protein in an interacting module (with a high clustering coefficient (yellow/orange color on Additional file 8: Figure S6) (for example the Wnt or the Lipoprotein signaling and cholesterol metabolism module). Interrogation of the STITCH database (Search Tools for Interactions of Chemicals) [29] for associations between deregulated genes in CRC and the 3 chemotherapeutic drugs tested in our study showed that these two types of genes could be targeted (Additional file 10). On the other hand, none of the drugs targeted members from the Wnt signaling pathway, except CCND1 that could be affected by Oxaliplatin and 5-Fluorouracile [56–58]. Anticancer agents targeting Wnt/β-catenin signaling have been developed in recent years and their use could be proposed in combination with classical colon cancer therapies. As expected, in light of our network analysis, the use of Lovastatin seems to be very interesting to target members of the Lipoprotein signaling and cholesterol metabolism pathway. Nevertheless, in absence of a comprehensive analysis of the effects of the drugs on several additional endpoints (apoptosis, gene/protein expression profiling, etc.), it is not possible to conclude on a direct role of the genes analyzed in the control of cell death and, specifically, to ascertain that the observed cytotoxicity after 72 h is directly linked to the deregulation of gene expression after 24 h of treatment. It should also be kept in mind that our data were obtained in a single cell model, and cannot be taken as fully conclusive, as this study did not address the point in the target population, i.e. human subjects. One possibility to approach this goal could be to conduct similar analyses in several other CRC cell lines and/or animals bearing human CRC (Additional file 11).

In addition, although our cohort of CRC subjects fitted well with the French population in terms of age at CRC diagnosis and sex ratio, several potentially confounding factors were not taken into account, as the data were not available to us. We should mention that we could not figure out if the RNAseq reported data on CRC patients from TCGA have taken any of these concerns into account. These included obesity (BMI above 30), which affects roughly 18.7% of the population over 65 in France, or concomitant drug absorption that occurs in virtually all subjects of 65 and above. Obesity and drugs (antidiuretics, cholesterol-lowering drugs, anti-diabetic drugs, or any combination of these) are known to modulate gene expression, at least in experimental models [59–62], but probably less so than the chemotherapeutic drugs used in our study, which act genome-wide. Although it would certainly be a major challenge to constitute homogenous groups of CRC patients on a given drug, at a given dose, for a given time, smoking or not, having alcohol drinking habits, etc., with or without a BMI above 30, this would in principle be attainable. One way to approach such composite situations could possibly be to use model systems, as CRC cells in culture or laboratory animals bearing human tumors. Further prospective recruitment of CRC patients could readily compare lean vs. obese patients using the methodology used in this manuscript.

## Conclusion

In conclusion to this work, we have obtained selected transcriptome data through a dedicated PCR array approach comparing colon cancer samples with normal colon mucosae. Although the majority of deregulations were already reported in CRC, about 10% of those events were not known to occur in colon cancer or in cancer in general. The genes most important to the structures of the networks indeed were known to play important regulatory roles in the tumors, but some were new, most significantly from the Lipoprotein signaling and cholesterol metabolism pathway. Use of this array also revealed that eight genes from this pathway were not engaged in any interaction within the corresponding sub-network or with other networks. This observation, which likely reflects the smaller number of investigations on those genes, also opens the path to uncover novel targets to treat colon cancer, potentially through the use of lipid-lowering drugs such as statins whose potential in combination with anticancer drugs is under scrutiny through clinical trials, as recorded at the National Cancer Institute.

## Additional files

Additional file 1: Table S1. List of genes from the five different RT² Profiler™ PCR Array Human Systems used in this study, classified in functional groups. See manufacturer’s information website (http://www.sabiosciences.com/PCRArrayPlate.php): A, Apoptosis (PAHS-012A); B, Cancer Pathway Finder (PAHS-033A); C, Lipoprotein Signaling & Cholesterol Metabolism (PAHS-080Z); D, Drug Metabolism (PAHS-002A); E, Wnt Signaling Pathway (PAHS-043A). Gene expression level deregulations uncovered in this study were indicated by red or green color for an up-regulation or a down-regulation, respectively, in CRC as compared to NT. (DOCX 25 kb)

Additional file 2: Figure S1. Hierarchical clustering of colorectal carcinoma (CRC) and normal colon tissue (NT) based on gene expression profiling obtained from each type of PCR array. Log2-transformed fold-change values from genes fulfilling the criteria of PCR Data Analysis (Qiagen) obtained for each specific PCR array (fold-change > 1.7 and q < 0.05) were analyzed with Cluster and Treeview, using centered correlation and average linkage [63]. Red and green colors indicate transcript levels above and below the median values, respectively. NT, normal colon tissue (n=19) and CRC, colorectal carcinoma (n=95). Tumor samples were identified by a number followed by the Tumor Bank running number (S0, S1, S2, S3, S4) corresponding to the grading stage according to the pathological classification. Genes identified by their gene symbol appear on the right side of each panel. Each column gives the gene expression profile of a sample, and each line indicates the variations in the level of expression of a given gene among tissue samples. The length of the branches of the trees forming the dendrograms on the top of each panel reflects the degree of similarity between samples; the longer the branch, the larger the difference in gene expression. A, Hierarchical clustering based on 14 deregulated genes from the “Apoptosis” PCR array; B, Hierarchical clustering based on 33 deregulated genes from the “Cancer Pathway” PCR array; C, Hierarchical clustering based on 29 deregulated genes from the “Lipoprotein signaling and cholesterol metabolism” PCR array; D, Hierarchical clustering based on 23 deregulated genes from the “Drug metabolism” PCR array; E, Hierarchical clustering based on 18 deregulated genes from the “Wnt pathway” PCR array. (ZIP 2488 kb)

Additional file 3: Table S2. Comparative differential expression data focused on genes identified on RT² Profiler™ PCR Array analysis and previously published data. Comparative expression data of selected genes deregulated in CRC as compared to NT in RT² Profiler™ PCR Array analysis, related to Apoptosis (2A, n=14), Cancer Pathway (2B, n= 33), Lipoprotein signaling and Cholesterol metabolism (2C, n=29), Drug metabolism (2D, n=23) and Wnt signaling pathway (2E, n=218). Differential expression data information was listed for each gene: i) from our study performed by PCR array technology in CRC (n=95) as compared to NT (n=19); ii) from our previous study performed with whole-genome microarray technology in CRC (n=25), CRA (n=55) and NT paired to CRA or CRC (n=27) [16]; iii) from microarray expression data of COADREAD cohort of TCGA consortium (153 colon and 69 rectal carcinomas as compared to 22 NT) ([17], gdac.broadinstitute.org) iv) from literature data by focusing on expression analysis obtained in CRC (RNA or protein level) and v) from other literature data obtained from genetic association, epigenetic and functional studies. Underlined gene names indicated that the genes were largely referenced in PubMed in association with colorectal cancer. (DOCX 299 kb)

Additional file 4: Figure S2. Principal component analysis of colorectal dataset of TCGA based on the expression of genes identified in our study. Principal component analysis (PCA) was performed on expression data retrieved from colorectal cohort from TCGA ([17], gdac.broadinstitute.org) composed of 22 NT, 153 colon carcinoma and 69 rectal carcinoma. PCA were performed on different sets of deregulated genes according to functional group: Panel A) Apoptosis (14 genes); Panel B) Cancer pathway (33 genes); Panel C) Lipoprotein signaling and cholesterol metabolism (29 genes); Panel D) Drug metabolism (23 genes); Panel E) Wnt signaling pathway (18 genes); Panel F) all genes identified in our study (111 genes). Individuals are colored according to a categorical variable in each PCA plot: i) according to histological type (NT: green, colon carcinoma: red, rectal carcinoma: blue); ii) according to grading stage (I: red, II: green, III: blue, IV: light blue); iii) according to mutational status of APC and KRAS (wild type: red, mutated: green). Samples corresponding to NT and tumors that present no available information about stage and mutational status are colored in black. (PDF 494 kb)

Additional file 5: Figure S3. Western blot analysis of protein level in paired colorectal carcinoma (CRC) and normal colon mucosae (NT). Forty µg of protein extracts from 20 CRC and paired NT samples were subjected to Western blot analysis using selected antibodies and a monoclonal HSC70 antibody (for normalization). Tumor and normal tissue were analyzed simultaneously. Quantification of protein levels was performed by measuring the fluorescence intensity (Odyssey Li-Cor). A. Western blot analysis of 9 protein-coding genes previously identified as up-regulated in CRC as compared to NT by PCR array: BCL2L1, NME1, PKM2, GSTP1, GPI, FDPS, HMGCS1, CYP39A1 and PCSK9. B. Western blot analysis of 4 protein-coding genes previously identified as down-regulated in CRC as compared to NT by PCR array: BCL2, CASP7, IGF1 and ADH1C. The numbers on the left identified the samples; the numbers at the bottom indicate the number of samples that showed a similar regulation at both the mRNA and the protein levels. C. Comparison of average fold-changes between CRC and paired NT (n=20) obtained from transcriptome (PCR array) and proteomic (Western blot) analyses. Error bars represent the Standard Error of the Mean (SEM). The upper panel represents data from 8 up-regulated genes and the lower panel represents data from 4 down-regulated genes in CRC as compared to NT. (PDF 223 kb)

Additional file 6: Figure S4. Representative immunostaining pattern obtained for NT and CRC paired tissues. A. Strong cytoplasmic NME1-staining of the adenocarcinomatous glands (right) compared to the normal adjacent colonic glands (left) (x50, NME1 immunohistochemistry, hematoxylin counter coloration). B. Strong cytoplasmic FDPS-staining of the adenocarcinomatous glands (right) compared with the normal adjacent colonic glands (left) (x50, FDPS immunohistochemistry, hematoxylin counter coloration). C. Strong cytoplasmic and nuclear CCND1-staining (known to be over-expressed in CRC) of the adenocarcinomatous glands (left) compared with the normal adjacent colonic glands (right) (x50, CCND1 immunohistochemistry, hematoxylin counter coloration). (PDF 252 kb)

Additional file 7: Figure S5. Network building from all deregulated genes.A. Interactions between 111 deregulated genes identified by transcriptome comparison of CRC and NT were retrieved from the STRING database (version 10.5). The interactions include direct (physical) and indirect (functional) associations. All interaction sources questioned by STRING were used and the minimum required interaction score was 0.4 (medium confidence). The blue color of edges indicated a physical interaction in the source of evidence between 2 nodes. The thickness of edges was correlated with the score confidence (large for high score). The node shape illustrates the specific PCR array: Apoptosis: square, Cancer Pathway: hexagon, Lipoprotein signaling and cholesterol metabolism: circle, Drug metabolism: diamond and Wnt pathway: octagon. The color intensity of nodes was related to transcriptome deregulation (red for up-regulation and green for down-regulation in CRC as compared to NT). Genes showing a deregulation in more than 75% of CRC were characterized by a bold line of shape node.B. Node degree distribution of 111 genes-based network. The X axis shows the degree of a node, while the Y axis shows the number of nodes for each degree in the network. This scatter plot highlights the molecular hubs of the network. Molecular hubs showing a deregulation in more than 75% of CRC were indicated by a black symbol. (PDF 234 kb)

Additional file 8: Figure S6. Topological parameters analysis on global network building from the functional association between 111 genes deregulated in CRC vs. NT. A. Network visualization of node degree and clustering coefficient parameters. The node degree, i.e. the number of edges linked to a node, was visually mapped by the node size: nodes with a low degree were smaller, in contrast to nodes with a high degree. The clustering coefficient, another computed topological parameter reflecting the tendency of neighbors of each node to interact together, was mapped by node gradient color: nodes with lowest clustering coefficient are blue and nodes with highest clustering coefficient are orange (medium clustering coefficient are yellow). As mentioned in Additional file 7: Figure S5, the thickness of edges was correlated with score confidence (large for high score), the blue line indicated physical interaction experimentally demonstrated in the source of evidence and the node shape illustrated the specific PCR array: Apoptosis: square, Cancer Pathway: hexagon, Lipoprotein signaling and cholesterol metabolism: circle, Drug metabolism: diamond and Wnt pathway: octagon. B. Scatter plot displaying the correlation between node degrees and clustering coefficients in the global network composed of 111 nodes showing transcriptome deregulation in CRC as compared to NT. Dotted lines indicated the median of node degree (value of 11) and the median of clustering coefficient (value of 0.54). Genes showing a deregulation in more than 75% of CRC were indicated by a black symbol. (PDF 300 kb)

Additional file 9: Figure S7. Viability assay of colorectal cell lines treated by chemotherapeutic drugs combined or not with Lovastatin. The viability of two colorectal cell lines (A: HCT-116, B: HT-29) was evaluated by MTT assays after 72h of treatment by different chemotherapeutic drugs combined (black bars) or not (white bars) with Lovastatin (5 µM). Control cells were treated by DMSO (less than 0.1% final concentration). Two different concentrations were used for the chemotherapeutic drugs: 1 µM and 10 µM for Oxaliplatin and 5-Fluorouracile, 0.01 µM and 0.1 µM for Camptothecin. The results are from three independent experiments. Error bars represent the SEM. (PDF 576 kb)

Additional file 10: Figure S8. Gene-Drug interaction network. Interactions were retrieved in STITCH v5 (Search Tools for Interactions of Chemicals) between deregulated genes in CRC and 4 drugs: 3 chemotherapeutic drugs used in CRC treatment (5-Fluorouracile (5-FU), Oxaliplatin (OXA) and Camptothecin (CPT), and Lovastatin (LOVA). To facilitate network visualization, we have hidden edges between genes. Node size and node color reflect the interaction degree and the clustering coefficient characterizing nodes in 111 genes based-network presented in Additional file 8. The thickness of edges was correlated with score confidence (large for high score) retrieved in STITCH database (version 5) and the blue color of edges indicated a physical interaction in the source database. The bold line of shape node characterizes genes deregulated in more than 75% of CRC. (PDF 93 kb)

Additional file 11: Table S3. Raw quantitative-PCR data for each PCR array used in this study. Clinical data associated to CRC samples were indicated in the excel file. (XLSX 412 kb)

## Abbreviations

CRC: Colorectal carcinoma
FC: Fold-change
NT: Normal Tissue
STITCH: Search Tools for Interactions of Chemicals
STRING: Search Tool for the Retrieval of Interacting Genes/Proteins
