# Supplementary material for: A transcriptome-based protein network that identifies new therapeutic targets in colorectal cancer
Source: BMC Genomics. 2017 Sep 30;18:758. doi: 10.1186/s12864-017-4139-y (PMC5622428; doi:10.1186/s12864-017-4139-y)
Supplement: Supplementary file 1 — List of genes from the five different RT² Profiler™ PCR Array Human Systems used in this study, classified in functional groups. See manufacturer’s information website (http://www.sabiosciences.com/PCRArrayPlate.php): A, Apoptosis (PAHS-012A); B, Cancer Pathway Finder (PAHS-033A); C, Lipoprotein Signaling & Cholesterol Metabolism (PAHS-080Z); D, Drug Metabolism (PAHS-002A); E, Wnt Signaling Pathway (PAHS-043A). Gene expression level deregulations uncovered in this study were indicated by red or green color for an up-regulation or a down-regulation, respectively, in CRC as compared to NT. (DOCX 25 kb) [file 12864_2017_4139_MOESM1_ESM.docx]

**Additional file 1: Table S1. List of genes composing five different RT² Profiler™ PCR Array Human Systems used in this study, classified in functional groups.**

Table S1.A. List of genes in the RT² Profiler™ PCR Array Human Apoptosis classified in functional groups.

| **Apoptosis (PAHS-012A)** | **Induction of Apoptosis** | **Death Domain Receptors** | CRADD, FADD, TNF, TNFRSF10B (DR5). |
| --- | --- | --- | --- |
|  |  | **DNA Damage** | ABL1, CIDEA, CIDEB, TP53, TP73. |
|  |  | **Extracellular Signals** | CFLAR (CASPER), DAPK1, TNFRSF25 (DR3). |
|  |  | **Other** | BAD, BAK1, BAX, BCL10, BCL2L11, BCLAF1, BID, BIK, BNIP1, BNIP3, BNIP3L, CASP1 (ICE), CASP10 (MCH4), CASP14, CASP2, CASP3, CASP4, CASP6, CASP8, CD27 (TNFRSF7), CD70 (TNFSF7), DFFA, FAS (TNFRSF6), FASLG (TNFSF6), GADD45A, HRK, LTA (TNFB), NOD1 (CARD4), PYCARD (TMS1/ASC), TNFRSF10A, TNFRSF9, TNFSF10 (TRAIL), TNFSF8, TP53BP2, TRADD, TRAF3. |
|  | **Anti-Apoptosis** | | AKT1, BAG1, BAG3, BAG4, BAX, BCL2, BCL2A1 (Bfl-1/A1), BCL2L1 (BCL-X), BCL2L10, BCL2L2, BFAR, BIRC3 (c-IAP1), BIRC6, BIRC8, BNIP1, BNIP2, BNIP3, BNIP3L, BRAF, CD27 (TNFRSF7), CD40LG (TNFSF5), CFLAR (CASPER), DAPK1, FAS (TNFRSF6), HRK, IGF1R, MCL1, NAIP (BIRC1), NOL3, RIPK2, TNF, XIAP (BIRC4). |
|  | **Regulation of Apoptosis** | **Negative Regulation** | BAG1, BAG3, BAG4, BCL10, BCL2, BCL2A1 (Bfl-1/A1), BCL2L1 (BCL-X), BCL2L10, BCL2L2, BFAR, BIRC3 (c-IAP1), BIRC6, BIRC8, BNIP1, BNIP2, BNIP3, BNIP3L, BRAF, CASP3, CD27 (TNFRSF7), CD40LG (TNFSF5), CFLAR (CASPER), CIDEA, DAPK1, DFFA, FAS (TNFRSF6), IGF1R, MCL1, NAIP (BIRC1), NOL3, TP53, TP73, XIAP (BIRC4). |
|  |  | **Positive Regulation** | ABL1, AKT1, BAD, BAK1, BAX, BCL2L11, BCLAF1, BID, BIK, BNIP3, BNIP3L, CASP1 (ICE), CASP10 (MCH4), CASP14, CASP2, CASP4, CASP6, CASP8, CD70 (TNFSF7), CIDEB, CRADD, FADD, FASLG (TNFSF6), HRK, LTA (TNFB), NOD1 (CARD4), PYCARD (TMS1/ASC), RIPK2, TNF, TNFRSF10A, TNFRSF10B (DR5), TNFRSF25 (DR3), TNFRSF9, TNFSF10 (TRAIL), TNFSF8, TP53, TP53BP2, TRADD, TRAF2, TRAF3, TRAF4. |
|  | **TNF/TNFR Domain Proteins** | | CD27 (TNFRSF7), CD40 (TNFRSF5), FAS (TNFRSF6), FASLG (TNFSF6), LTA (TNFB), LTBR, TNF, TNFSF10 (TRAIL), TNFSF8, TNFRSF10A, TNFRSF10B (DR5), TNFRSF11B, TNFRSF21, TNFRSF25 (DR3), TNFRSF9. |
|  | **BCL2 and BAG Domain Proteins** | | BAG1, BAG3, BAG4, BAX, BCL2, BCL2A1 (Bfl-1/A1), BCL2L1 (BCL-X), BCL2L10, BCL2L11, BCL2L2, MCL1. |
|  | **BIR Domain Proteins** | | BIRC2 (c-IAP2), BIRC3 (c-IAP1), BIRC6, BIRC8, NAIP (BIRC1), XIAP (BIRC4). |
|  | **CARD Domain Proteins** | | APAF1, BCL10, BIRC2 (c-IAP2), BIRC3 (c-IAP1), CARD6, CARD8, CASP1 (ICE), CASP2, CASP4, CASP5, CASP9, CRADD, NOD1 (CARD4), NOL3, PYCARD (TMS1/ASC), RIPK2. |
|  | **DEATH Domain Proteins** | | CRADD, DAPK1, FADD, TNFRSF10A, TNFRSF10B (DR5), TNFRSF11B, TNFRSF1A, TNFRSF21, TNFRSF25 (DR3), TRADD. |
|  | **TRAF Domain Proteins** | | TRAF2, TRAF3, TRAF4. |
|  | **Caspases and Regulators** | **Caspases** | CASP1 (ICE), CASP10 (MCH4), CASP14, CASP2, CASP3, CASP4, CASP5, CASP6, CASP7, CASP8, CASP9, CFLAR (CASPER), CRADD, PYCARD (TMS1/ASC). |
|  |  | **Caspase Activators** | APAF1, BAX, BCL2L10, CARD8, CASP1 (ICE), CASP9, NOD1 (CARD4), PYCARD (TMS1/ASC), TNFRSF10A, TNFRSF10B (DR5), TP53. |
|  |  | **Caspase Inhibitors** | CD27 (TNFRSF7), XIAP (BIRC4). |

Table S1.B. List of genes in the RT² Profiler™ PCR Array Human Cancer Pathway Finder classified in functional groups.

| **Cancer Pathway Finder (PAHS-033A)** | **Cell Cycle Control & DNA Damage Repair** | ATM, BRCA1, CCNE1 (cyclin E1), CDC25A, CDK2, CDK4, CDKN1A (p21Waf1), CDKN2A (p16Ink4), CHEK2 (chk2 / Rad53), E2F1, MDM2, RB1, S100A4, TP53 (p53). |
| --- | --- | --- |
|  | **Apoptosis and Cell Senescence** | APAF1, BAD, BAX, BCL2, BCL2L1 (bcl-X), CASP8, CFLAR (CASPER), FAS, GZMA, HTATIP2, TERT (telomerase), TNFRSF1A (TNF-a receptor), TNFRSF10B (DR5), TNFRSF25 (DR3). |
|  | **Signal Transduction Molecules and Transcription Factors** | AKT1, ERBB2, ETS2, FOS, JUN, MAP2K1 (MEK), MYC, NFKB1 (NFκB), NFKBIA (IκBα), PIK3R1 (PI3K p85α), RAF1, SNCG. |
|  | **Adhesion** | ITGA1 (integrin α1), ITGA2 (integrin α2), ITGA3 (integrin α3), ITGA4 (integrin α4), ITGAV (integrin αV), ITGB1 (integrin β1), ITGB3 (integrin β3), ITGB5 (integrin β5), MCAM, MTSS1, PNN, SYK, EPDR1. |
|  | **Angiogenesis** | ANGPT1 (angiopoietin-1), ANGPT2 (angiopoietin-2), COL18A1 (endostatin), FGFR2, IFNA1 (IFNα), IFNB1 (IFNβ), IGF1, IL8, PDGFA, PDGFB, TEK (tie-2), TGFB1, TGFBR1 (ALK-5), THBS1 (thrombospondin-1), TNF, VEGFA. |
|  | **Invasion and Metastasis** | MET, MMP1 (collagenase-1), MMP2 (gelatinase A), MMP9 (gelatinase B), MTA1, MTA2, NME1, NME4, PLAU, PLAUR, S100A4, SERPINB5 (maspin), SERPINE1 (PAI1), TIMP1, TIMP3, TWIST1. |

Table S1.C. List of genes in the RT² Profiler™ PCR Array Human Lipoprotein Signaling & Cholesterol Metabolism classified in functional groups.

| **Lipoprotein Signaling & Cholesterol Metabolism (PAHS-080Z)** | **LDL Receptors and Associated Proteins** | **LDL Receptors** | CXCL16, LDLR, LRP10, LRP12, LRP1B, LRP6, OLR1, STAB1, STAB2, VLDLR. |
| --- | --- | --- | --- |
|  |  | **LDL Receptor Associated Proteins** | LRPAP1, PCSK9, SNX17. |
|  | **LDL Associated Proteins** | | ANKRA2, APOA4, APOC3, CDH13, COLEC12, SCARF1, SORL1. |
|  | **HDL Associated Proteins** | | APOA1, APOD, APOF, APOL2, APOL5. |
|  | **Cholesterol Transport** | **Cholesterol Transporters** | ABCA1, ABCG1, APOA1, APOE, CETP, STARD3. |
|  |  | **Cholesterol Efflux** | ABCA1, ABCG1, APOA1, APOA4, APOC3, APOE. |
|  |  | **Reverse Cholesterol Transport** | ABCA1, APOA1, APOA2, APOA4, APOC3, APOE, CETP, LCAT. |
|  |  | **Other Genes Involved in Cholesterol Transport** | APOB, LDLR, NPC1L1, OSBPL5. |
|  | **Cholesterol Metabolism** | **Cholesterol Absorption** | CEL, LDLR, NPC1L1. |
|  |  | **Cholesterol Catabolism** | AKR1D1, APOE, CEL, CYP39A1, CYP46A1, CYP7A1, SCARF1, SNX17, TRERF1. |
|  |  | **Cholesterol Homeostasis** | ABCA1, ABCG1, ANGPTL3, APOA1, APOA2, APOA4, APOC3, APOE, CETP, LCAT, LDLR, LDLRAP1, PCSK9. |
|  |  | **Cholesterol Biosynthesis** | ACAA2, CNBP, CYB5R3 (DIA1), CYP51A1, DHCR24, DHCR7, FDFT1, FDPS, HMGCR, HMGCS1, HMGCS2, IDI1, IDI2, MVD, MVK, NPC1L1, NSDHL, PMVK, PRKAA1, PRKAA2, PRKAG2, TM7SF2. |
|  |  | **Other Genes Involved in Cholesterol Metabolism** | APOB, APOF, APOL1, APOL2, CYP11A1, CYP7B1, CELA3A, CELA3B, HDLBP, IL4, INSIG1, INSIG2, LEP, LIPE, MBTPS1, NR0B2, NR1H4, OSBPL1A, OSBPL5, PPARD, SCAP, SOAT1, SORL1, SREBF1, SREBF2, STARD3, VLDLR. |

Table S1.D. List of genes in the RT² Profiler™ PCR Array Human Drug Metabolism classified in functional groups.

| **Drug Metabolism (PAHS-002A)** | **Drug Transporters** | **Metallothioneins** | MT2A, MT3. |
| --- | --- | --- | --- |
|  |  | **P-Glycoprotein family** | ABCB1 (PGY1, mdr-1), ABCC1, GPI. |
|  | **Phase I Metabolizing Enzymes** | **P450 gene family** | CYP11B2, CYP17A1, CYP19A1, CYP1A1, CYP2B6, CYP2C19, CYP2C8, CYP2C9, CYP2D6, CYP2E1, CYP2F1, CYP2J2, CYP3A5. |
|  | **Phase II Metabolizing Enzymes** | **Carboxylesterase** | CES2, CES1P1. |
|  |  | **Decarboxylase** | GAD1. |
|  |  | **Dehydrogenase** | ADH1B, ADH1C, ADH4, ADH5, ADH6, ALAD, ALDH1A1, HSD17B1, HSD17B2, HSD17B3. |
|  |  | **Glutathione peroxidases** | GPX1, GPX2, GPX3, GPX4, GPX5, GSTA3, GSTA4, GSTM2, GSTM3, GSTM5, GSTP1, GSTT1, GSTZ1, LPO, MPO. |
|  |  | **Lipoxygenase** | ALOX12, ALOX15, ALOX5, APOE. |
|  |  | **Hydrolases** | EPHX1, FAAH, FBP1. |
|  |  | **Kinases** | HK2, PKLR, PKM2. |
|  |  | **Oxidoreductases** | BLVRA, BLVRB, CYB5R3 (DIA1), GPX1, GPX2, GSR, MTHFR, NOS3, NQO1, SRD5A1, SRD5A2. |
|  |  | **Paraoxonase** | PON1, PON2, PON3. |
|  |  | **Glutathione S-Transferases** | GSTA3, GSTA4, GSTM2, GSTM3, GSTM5, GSTP1, GSTT1, MGST1, MGST2, MGST3. |
|  |  | **Transferases** | CHST1, NAT1, NAT2, COMT, GGT1. |
|  | **Other Related Genes** | | ABP1, AHR, ARNT, ASNA1, GCKR, MARCKS, SMARCAL1, SNN. |

Table S1.E. List of genes in the RT² Profiler™ PCR Array Human Wnt Signaling Pathway classified in functional groups.

| **Wnt Signaling Pathway (PAHS-043A)** | **WNT Signaling Pathway** | **Canonical** | AES (TLE/Groucho), APC, AXIN1, BCL9 , CSNK1A1, CSNK1D, CSNK1G1, CSNK2A1, CTBP1, CTBP2, CTNNB1, CTNNBIP1 (ICAT), CXXC4, DIXDC1, DKK1, DVL1, DVL2, EP300, FRAT1, FZD1, FZD2, FZD3, FZD4, FZD5, FZD6, FZD7, FZD8, GSK3A, GSK3B, LEF1, LRP5, LRP6, NKD1, PORCN, PPP2CA, PPP2R1A, PYGO1, SENP2, SFRP1, SFRP4, SOX17, TCF7, TCF7L1, WIF1, WNT1, WNT10A, WNT16, WNT2, WNT2B, WNT3, WNT3A, WNT4, WNT6, WNT7A, WNT7B, WNT8A. |
| --- | --- | --- | --- |
|  |  | **Planar Cell Polarity (PCP)** | DAAM1, DVL1, DVL2, NKD1, RHOU, WNT9A. |
|  |  | **WNT/Ca2+** | FZD2, WNT1, WNT10A, WNT11, WNT16, WNT2, WNT2B, WNT3, WNT3A, WNT4, WNT5A, WNT5B, WNT6, WNT7A, WNT7B, WNT8A, WNT9A. |
|  | **WNT Signaling Negative Regulation** | | APC, AXIN1, BTRC (bTrCP), CCND1, CTBP1, CTBP2, CTNNBIP1 (ICAT), CXXC4, DKK1, FBXW11, FBXW2, FBXW4, FRZB (FRP-3), KREMEN1, LRP6, NLK, NKD1, SENP2, SFRP1, SFRP4, SOX17, TLE1, TLE2, WIF1. |
|  | **WNT Signaling Target Genes** | | BTRC (bTrCP), CCND1, CCND2, CCND3, FOSL1 (FRA-1), JUN, MYC, PITX2, T (Brachyury). |
|  | **Developmental Processes** | **Cell Fate** | CTNNB1, DKK1, WNT1, WNT3, WNT3A. |
|  |  | **Tissue Polarity** | FZD2, FZD3, FZD5, FZD6. |
|  |  | **Cell Growth & Profileration** | APC, CCND1, CCND2, CCND3, CTBP1, CTBP2, CTNNB1, CTNNBIP1 (ICAT), EP300, FGF4, FOSL1, FOXN1, FSHB, FZD3, JUN, LRP5, MYC, PPP2CA, PPP2R1A, T (Brachyury), WISP1, WNT3A. |
|  | **Cell Migration** | | APC, DKK1, LRP5, LRP6, WNT1. |
|  | **Cell Cycle** | | APC, BTRC (bTrCP), CCND1, CCND2, CCND3, CTNNB1, EP300, FOSL1, JUN, MYC, RHOU, TCF7L1. |
|  | **Cellular Homeostasis** | | APC, FZD2, JUN, MYC, SLC9A3R1. |
