# Supplementary figures and images for: A transcriptome-based protein network that identifies new therapeutic targets in colorectal cancer
Source: BMC Genomics. 2017 Sep 30;18:758. doi: 10.1186/s12864-017-4139-y (PMC5622428; doi:10.1186/s12864-017-4139-y)

Figure S1

A. Apoptosis

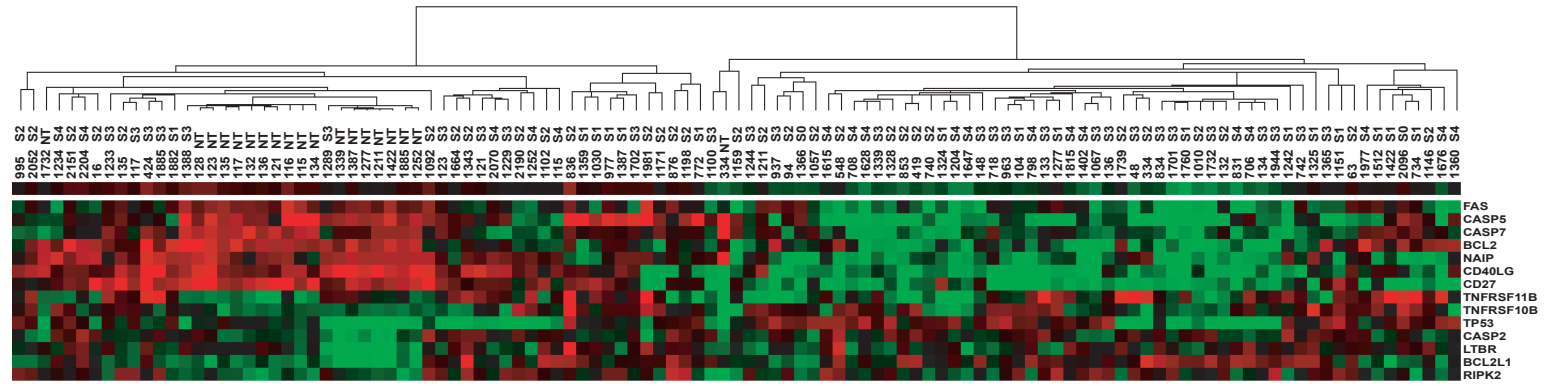

B. Cancer Pathway

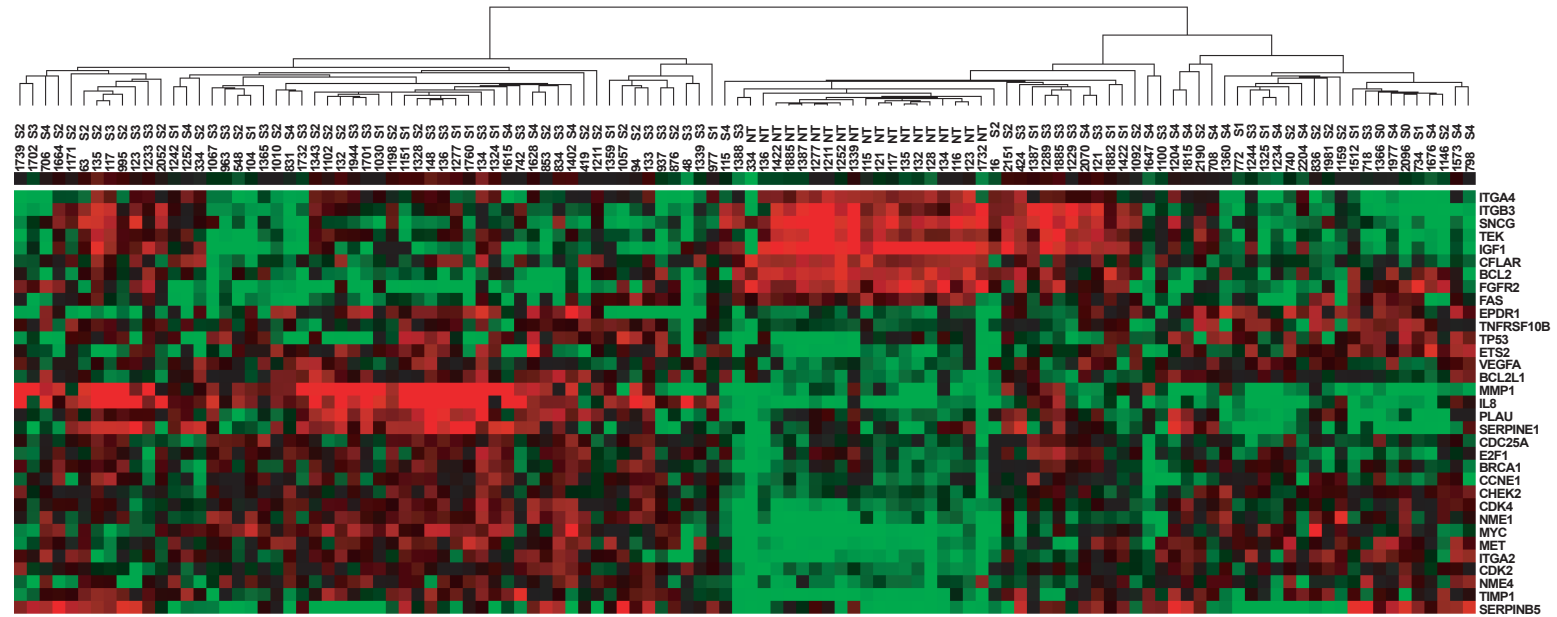

C. Lipoprotein Signaling and Cholesterol Metabolism

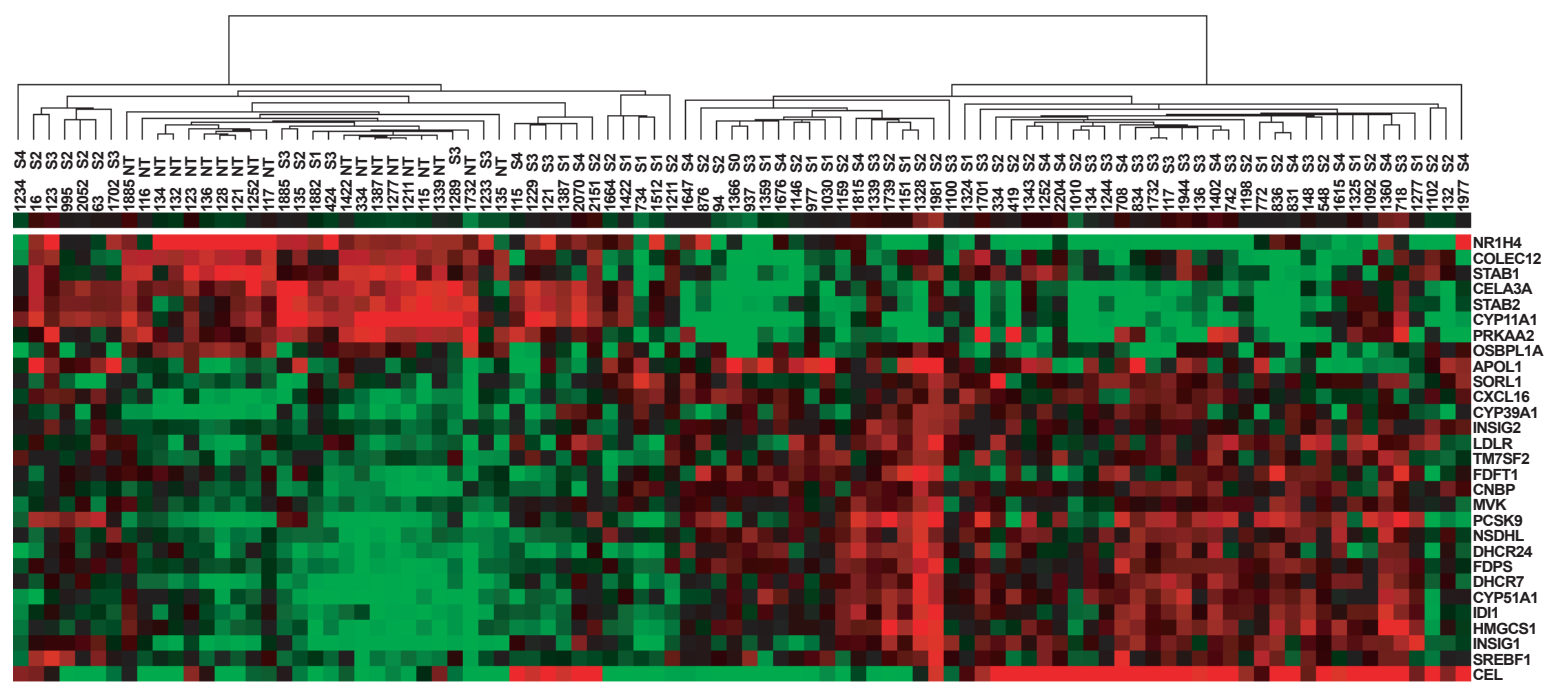

Supplement: Supplementary file 2 — Hierarchical clustering of colorectal carcinoma (CRC) and normal colon tissue (NT) based on gene expression profiling obtained from each type of PCR array. Log2-transformed fold-change values from genes fulfilling the criteria of PCR Data Analysis (Qiagen) obtained for each specific PCR array (fold-change > 1.7 and q < 0.05) were analyzed with Cluster and Treeview, using centered correlation and average linkage [63]. Red and green colors indicate transcript levels above and below the median values, respectively. NT, normal colon tissue (n=19) and CRC, colorectal carcinoma (n=95). Tumor samples were identified by a number followed by the Tumor Bank running number (S0, S1, S2, S3, S4) corresponding to the grading stage according to the pathological classification. Genes identified by their gene symbol appear on the right side of each panel. Each column gives the gene expression profile of a sample, and each line indicates the variations in the level of expression of a given gene among tissue samples. The length of the branches of the trees forming the dendrograms on the top of each panel reflects the degree of similarity between samples; the longer the branch, the larger the difference in gene expression. A, Hierarchical clustering based on 14 deregulated genes from the “Apoptosis” PCR array; B, Hierarchical clustering based on 33 deregulated genes from the “Cancer Pathway” PCR array; C, Hierarchical clustering based on 29 deregulated genes from the “Lipoprotein signaling and cholesterol metabolism” PCR array; D, Hierarchical clustering based on 23 deregulated genes from the “Drug metabolism” PCR array; E, Hierarchical clustering based on 18 deregulated genes from the “Wnt pathway” PCR array. (ZIP 2488 kb) [file 12864_2017_4139_MOESM2_ESM.zip › Additional file 2(S1_ABC).pdf]

D. Drug Metabolism

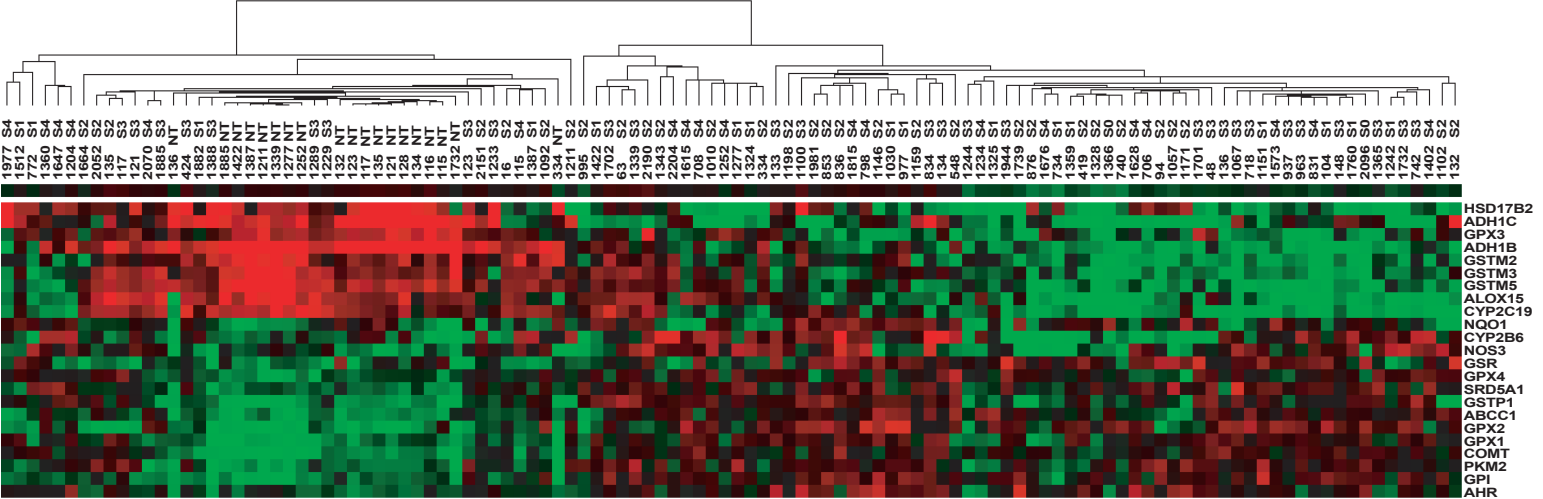

E. Wnt Signaling Pathway

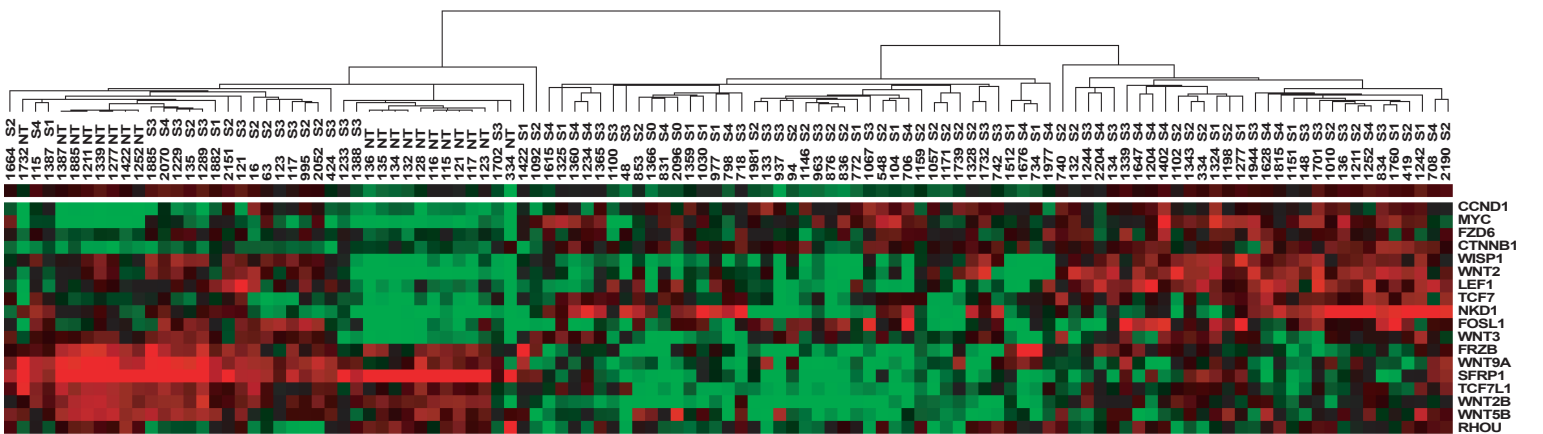

Supplement: Supplementary file 2 — Hierarchical clustering of colorectal carcinoma (CRC) and normal colon tissue (NT) based on gene expression profiling obtained from each type of PCR array. Log2-transformed fold-change values from genes fulfilling the criteria of PCR Data Analysis (Qiagen) obtained for each specific PCR array (fold-change > 1.7 and q < 0.05) were analyzed with Cluster and Treeview, using centered correlation and average linkage [63]. Red and green colors indicate transcript levels above and below the median values, respectively. NT, normal colon tissue (n=19) and CRC, colorectal carcinoma (n=95). Tumor samples were identified by a number followed by the Tumor Bank running number (S0, S1, S2, S3, S4) corresponding to the grading stage according to the pathological classification. Genes identified by their gene symbol appear on the right side of each panel. Each column gives the gene expression profile of a sample, and each line indicates the variations in the level of expression of a given gene among tissue samples. The length of the branches of the trees forming the dendrograms on the top of each panel reflects the degree of similarity between samples; the longer the branch, the larger the difference in gene expression. A, Hierarchical clustering based on 14 deregulated genes from the “Apoptosis” PCR array; B, Hierarchical clustering based on 33 deregulated genes from the “Cancer Pathway” PCR array; C, Hierarchical clustering based on 29 deregulated genes from the “Lipoprotein signaling and cholesterol metabolism” PCR array; D, Hierarchical clustering based on 23 deregulated genes from the “Drug metabolism” PCR array; E, Hierarchical clustering based on 18 deregulated genes from the “Wnt pathway” PCR array. (ZIP 2488 kb) [file 12864_2017_4139_MOESM2_ESM.zip › Additional file 2(S1_DE).pdf]

Figure S8

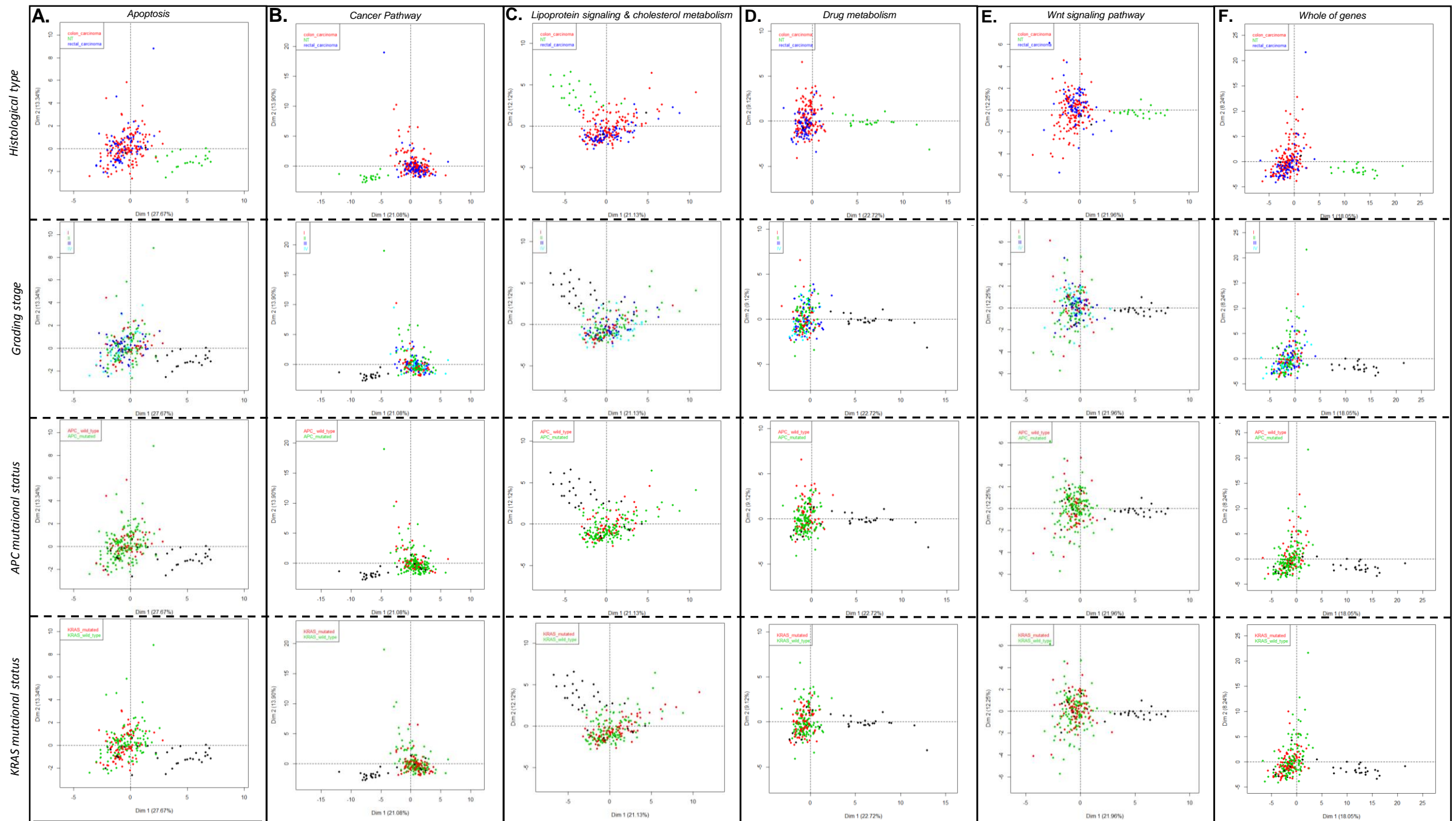

Supplement: Supplementary file 4 — Principal component analysis of colorectal dataset of TCGA based on the expression of genes identified in our study. Principal component analysis (PCA) was performed on expression data retrieved from colorectal cohort from TCGA ([17], gdac.broadinstitute.org) composed of 22 NT, 153 colon carcinoma and 69 rectal carcinoma. PCA were performed on different sets of deregulated genes according to functional group: Panel A) Apoptosis (14 genes); Panel B) Cancer pathway (33 genes); Panel C) Lipoprotein signaling and cholesterol metabolism (29 genes); Panel D) Drug metabolism (23 genes); Panel E) Wnt signaling pathway (18 genes); Panel F) all genes identified in our study (111 genes). Individuals are colored according to a categorical variable in each PCA plot: i) according to histological type (NT: green, colon carcinoma: red, rectal carcinoma: blue); ii) according to grading stage (I: red, II: green, III: blue, IV: light blue); iii) according to mutational status of APC and KRAS (wild type: red, mutated: green). Samples corresponding to NT and tumors that present no available information about stage and mutational status are colored in black. (PDF 494 kb) [file 12864_2017_4139_MOESM4_ESM.pdf]

**Figure S3**

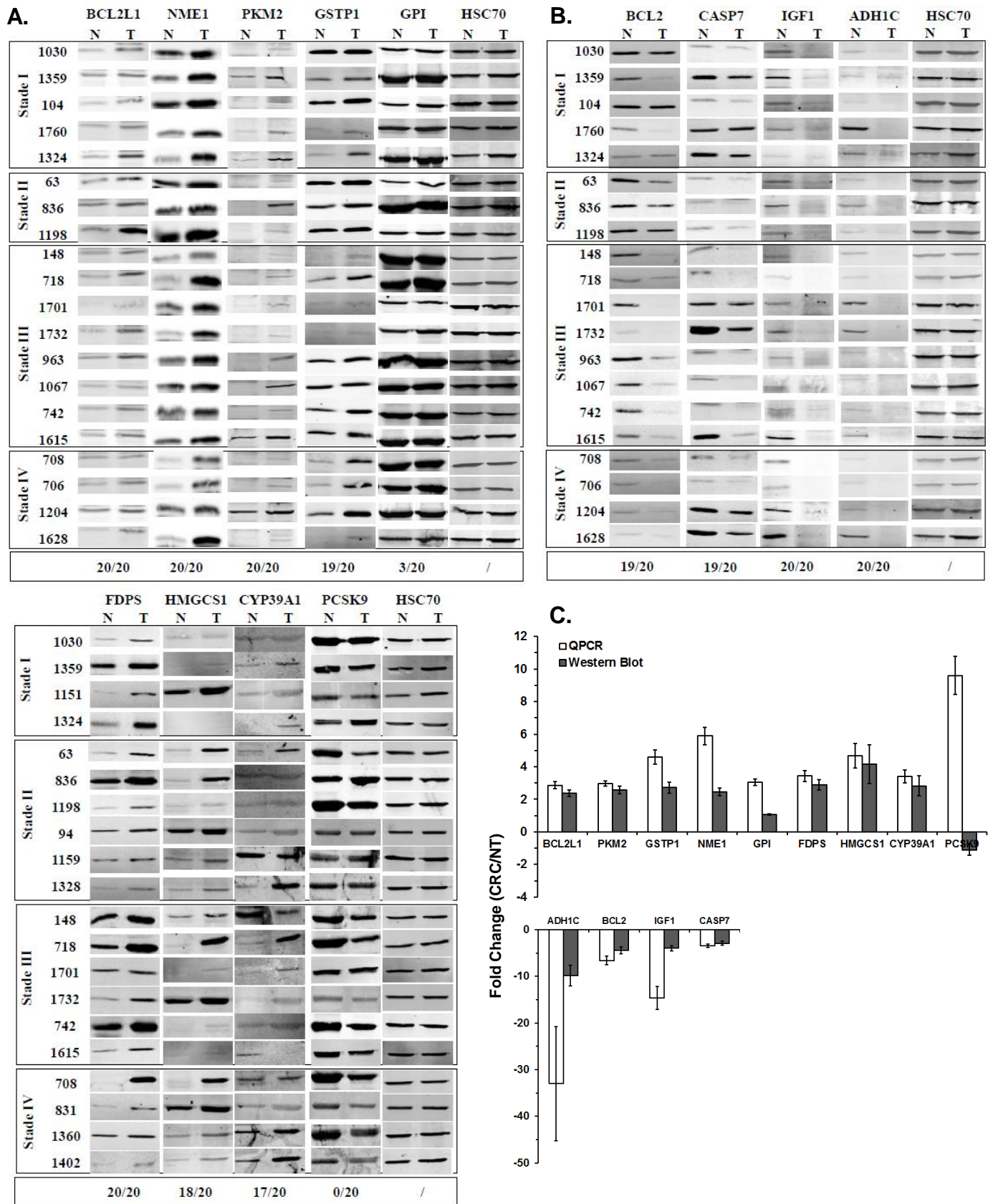

Supplement: Supplementary file 5 — Western blot analysis of protein level in paired colorectal carcinoma (CRC) and normal colon mucosae (NT). Forty µg of protein extracts from 20 CRC and paired NT samples were subjected to Western blot analysis using selected antibodies and a monoclonal HSC70 antibody (for normalization). Tumor and normal tissue were analyzed simultaneously. Quantification of protein levels was performed by measuring the fluorescence intensity (Odyssey Li-Cor). A. Western blot analysis of 9 protein-coding genes previously identified as up-regulated in CRC as compared to NT by PCR array: BCL2L1, NME1, PKM2, GSTP1, GPI, FDPS, HMGCS1, CYP39A1 and PCSK9. B. Western blot analysis of 4 protein-coding genes previously identified as down-regulated in CRC as compared to NT by PCR array: BCL2, CASP7, IGF1 and ADH1C. The numbers on the left identified the samples; the numbers at the bottom indicate the number of samples that showed a similar regulation at both the mRNA and the protein levels. C. Comparison of average fold-changes between CRC and paired NT (n=20) obtained from transcriptome (PCR array) and proteomic (Western blot) analyses. Error bars represent the Standard Error of the Mean (SEM). The upper panel represents data from 8 up-regulated genes and the lower panel represents data from 4 down-regulated genes in CRC as compared to NT. (PDF 223 kb) [file 12864_2017_4139_MOESM5_ESM.pdf]

**Figure S4**

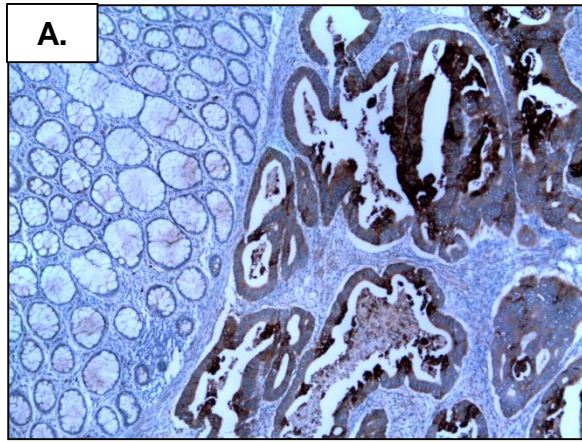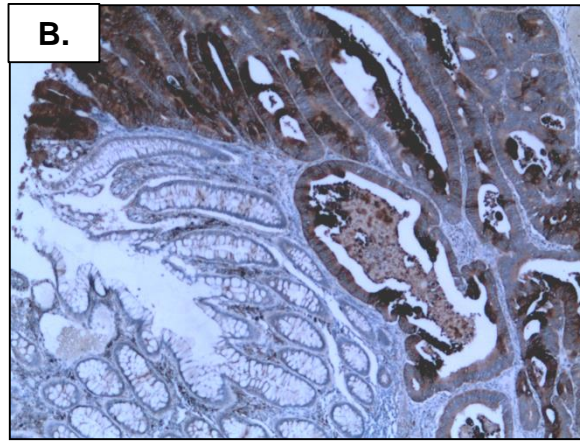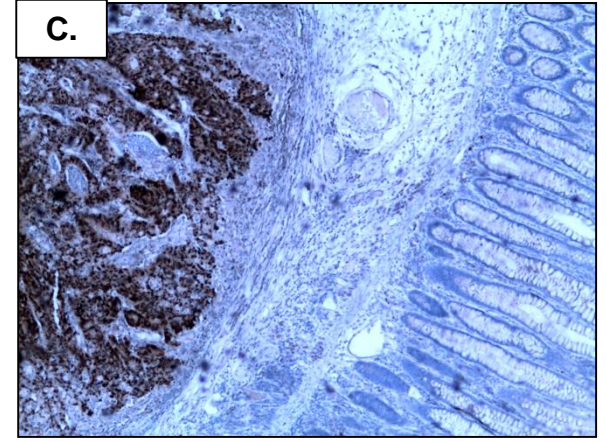

Supplement: Supplementary file 6 — Representative immunostaining pattern obtained for NT and CRC paired tissues. A. Strong cytoplasmic NME1-staining of the adenocarcinomatous glands (right) compared to the normal adjacent colonic glands (left) (x50, NME1 immunohistochemistry, hematoxylin counter coloration). B. Strong cytoplasmic FDPS-staining of the adenocarcinomatous glands (right) compared with the normal adjacent colonic glands (left) (x50, FDPS immunohistochemistry, hematoxylin counter coloration). C. Strong cytoplasmic and nuclear CCND1-staining (known to be over-expressed in CRC) of the adenocarcinomatous glands (left) compared with the normal adjacent colonic glands (right) (x50, CCND1 immunohistochemistry, hematoxylin counter coloration). (PDF 252 kb) [file 12864_2017_4139_MOESM6_ESM.pdf]

### Figure S5

**A.**

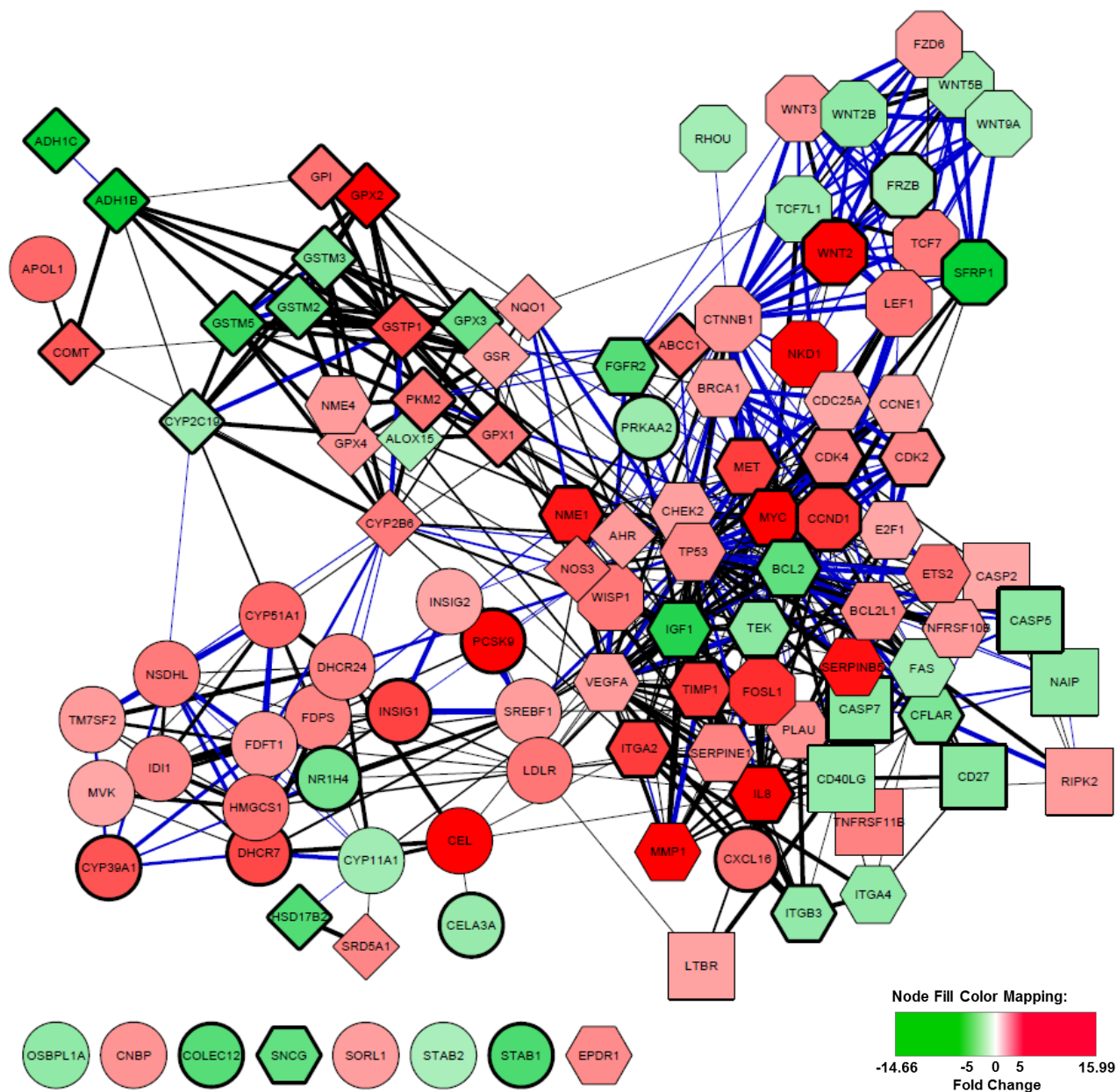

**B.**

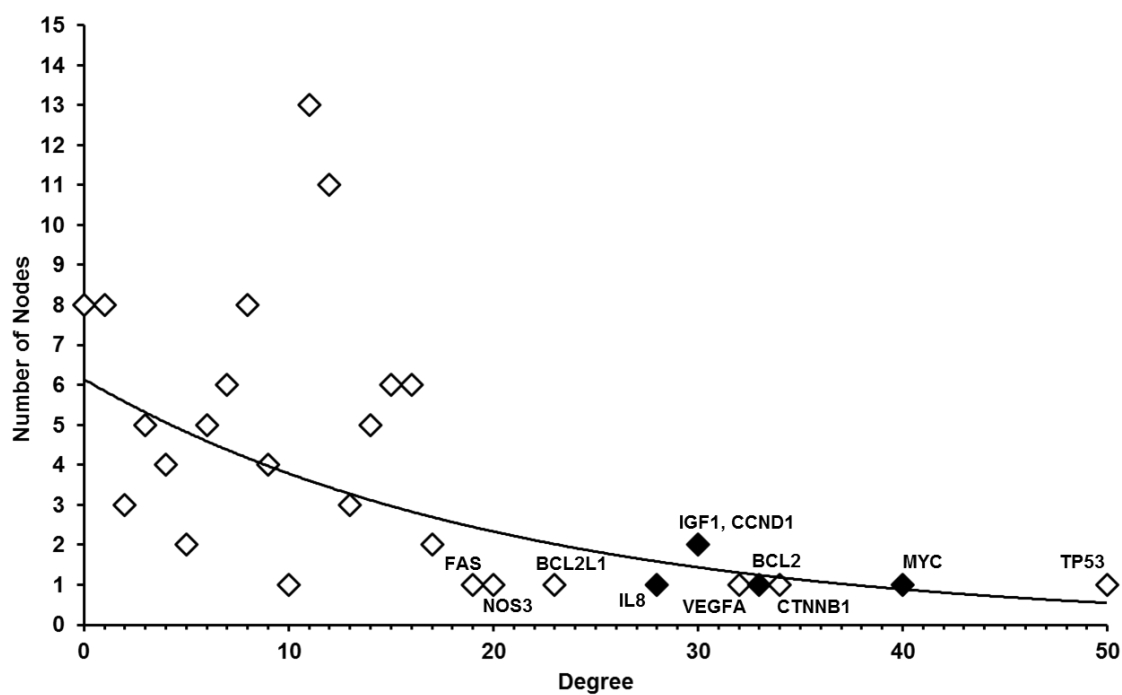

Supplement: Supplementary file 7 — Network building from all deregulated genes.A. Interactions between 111 deregulated genes identified by transcriptome comparison of CRC and NT were retrieved from the STRING database (version 10.5). The interactions include direct (physical) and indirect (functional) associations. All interaction sources questioned by STRING were used and the minimum required interaction score was 0.4 (medium confidence). The blue color of edges indicated a physical interaction in the source of evidence between 2 nodes. The thickness of edges was correlated with the score confidence (large for high score). The node shape illustrates the specific PCR array: Apoptosis: square, Cancer Pathway: hexagon, Lipoprotein signaling and cholesterol metabolism: circle, Drug metabolism: diamond and Wnt pathway: octagon. The color intensity of nodes was related to transcriptome deregulation (red for up-regulation and green for down-regulation in CRC as compared to NT). Genes showing a deregulation in more than 75% of CRC were characterized by a bold line of shape node.B. Node degree distribution of 111 genes-based network. The X axis shows the degree of a node, while the Y axis shows the number of nodes for each degree in the network. This scatter plot highlights the molecular hubs of the network. Molecular hubs showing a deregulation in more than 75% of CRC were indicated by a black symbol. (PDF 234 kb) [file 12864_2017_4139_MOESM7_ESM.pdf]

Figure S6

A.

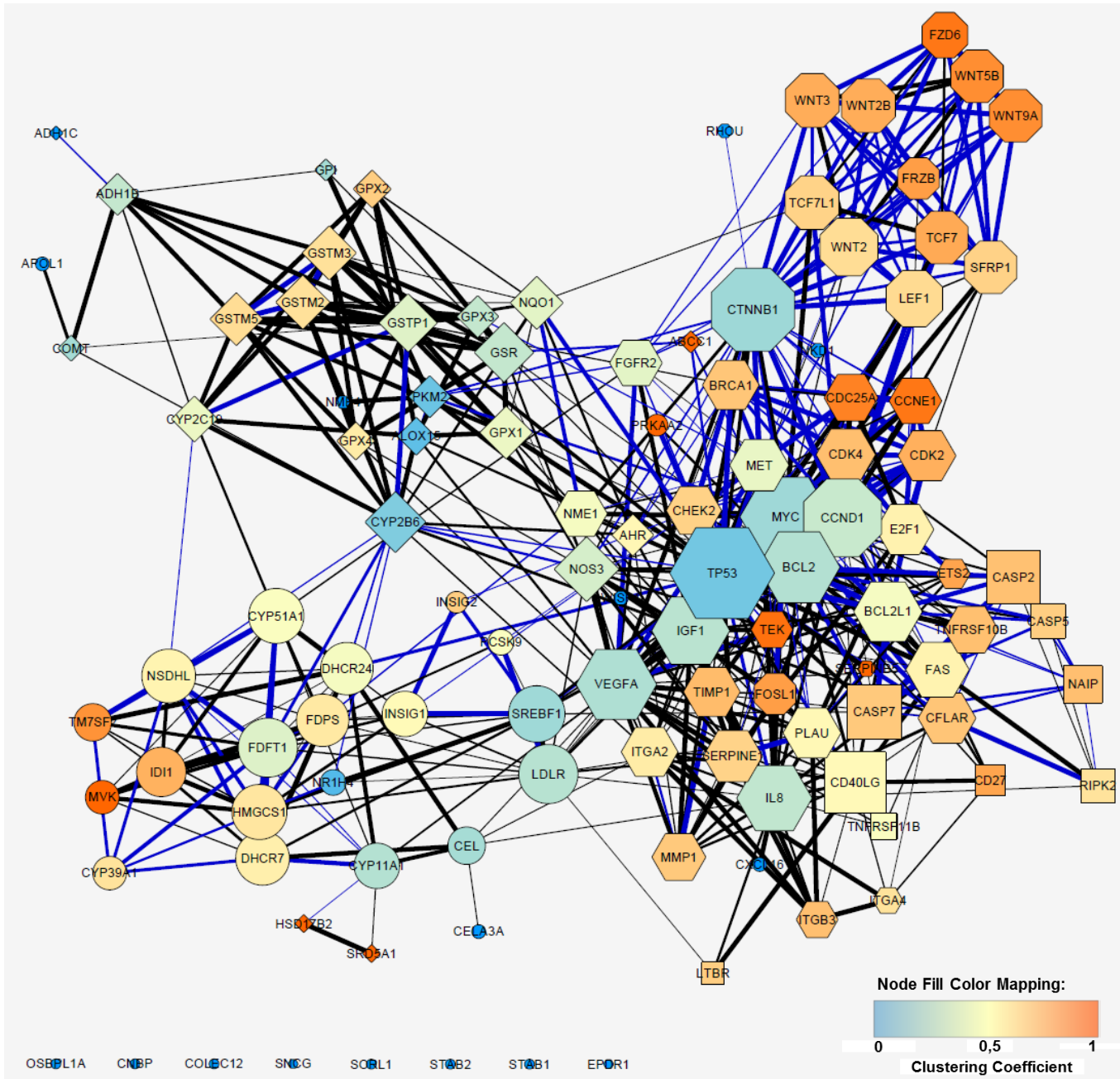

B.

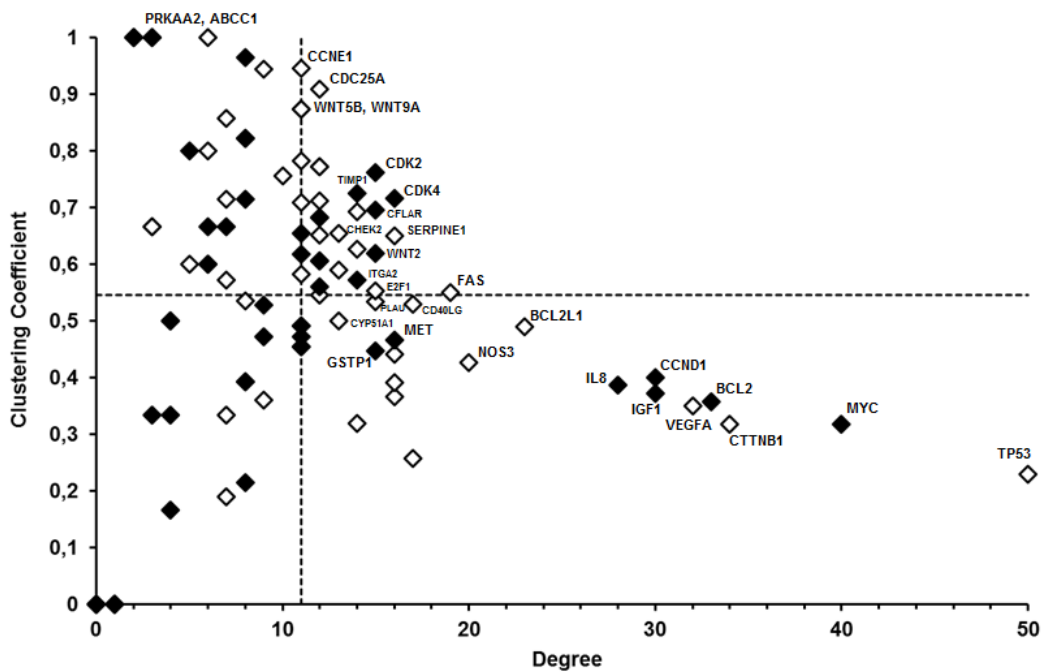

Supplement: Supplementary file 8 — Topological parameters analysis on global network building from the functional association between 111 genes deregulated in CRC vs. NT. A. Network visualization of node degree and clustering coefficient parameters. The node degree, i.e. the number of edges linked to a node, was visually mapped by the node size: nodes with a low degree were smaller, in contrast to nodes with a high degree. The clustering coefficient, another computed topological parameter reflecting the tendency of neighbors of each node to interact together, was mapped by node gradient color: nodes with lowest clustering coefficient are blue and nodes with highest clustering coefficient are orange (medium clustering coefficient are yellow). As mentioned in Additional file 7: Figure S5, the thickness of edges was correlated with score confidence (large for high score), the blue line indicated physical interaction experimentally demonstrated in the source of evidence and the node shape illustrated the specific PCR array: Apoptosis: square, Cancer Pathway: hexagon, Lipoprotein signaling and cholesterol metabolism: circle, Drug metabolism: diamond and Wnt pathway: octagon. B. Scatter plot displaying the correlation between node degrees and clustering coefficients in the global network composed of 111 nodes showing transcriptome deregulation in CRC as compared to NT. Dotted lines indicated the median of node degree (value of 11) and the median of clustering coefficient (value of 0.54). Genes showing a deregulation in more than 75% of CRC were indicated by a black symbol. (PDF 300 kb) [file 12864_2017_4139_MOESM8_ESM.pdf]

**Figure S7**

**A. HCT-116 colorectal cell line**

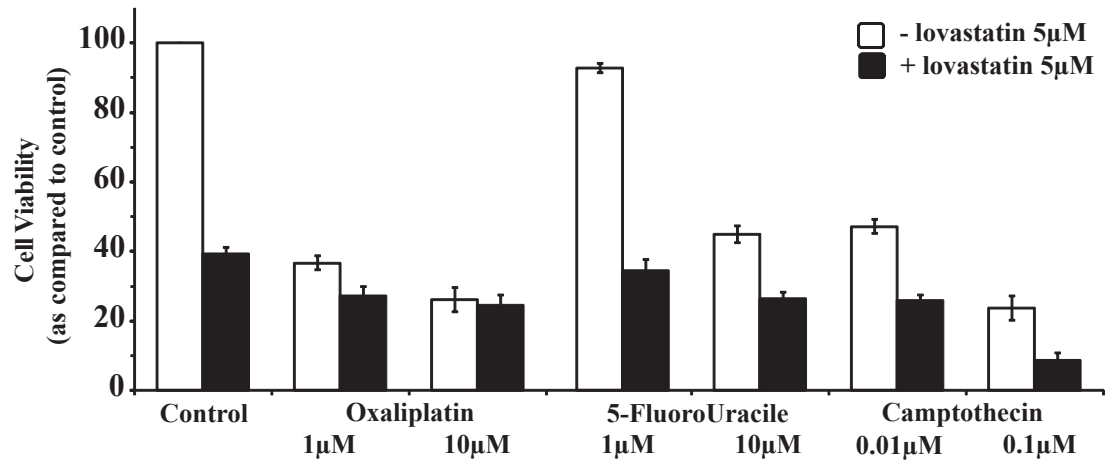

**B. HT-29 colorectal cell line**

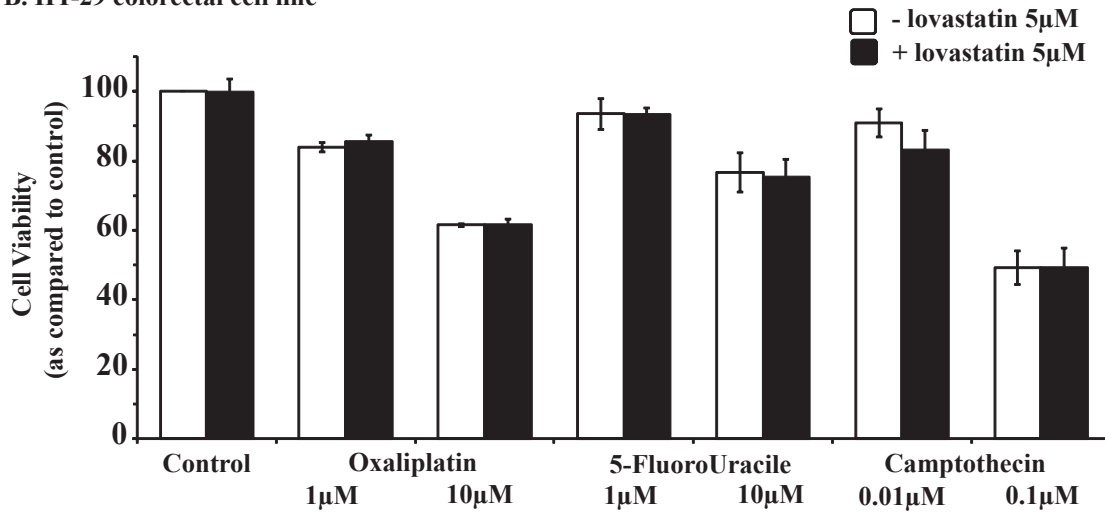

Supplement: Supplementary file 9 — Viability assay of colorectal cell lines treated by chemotherapeutic drugs combined or not with Lovastatin. The viability of two colorectal cell lines (A: HCT-116, B: HT-29) was evaluated by MTT assays after 72h of treatment by different chemotherapeutic drugs combined (black bars) or not (white bars) with Lovastatin (5 µM). Control cells were treated by DMSO (less than 0.1% final concentration). Two different concentrations were used for the chemotherapeutic drugs: 1 µM and 10 µM for Oxaliplatin and 5-Fluorouracile, 0.01 µM and 0.1 µM for Camptothecin. The results are from three independent experiments. Error bars represent the SEM. (PDF 576 kb) [file 12864_2017_4139_MOESM9_ESM.pdf]

## Figure S8

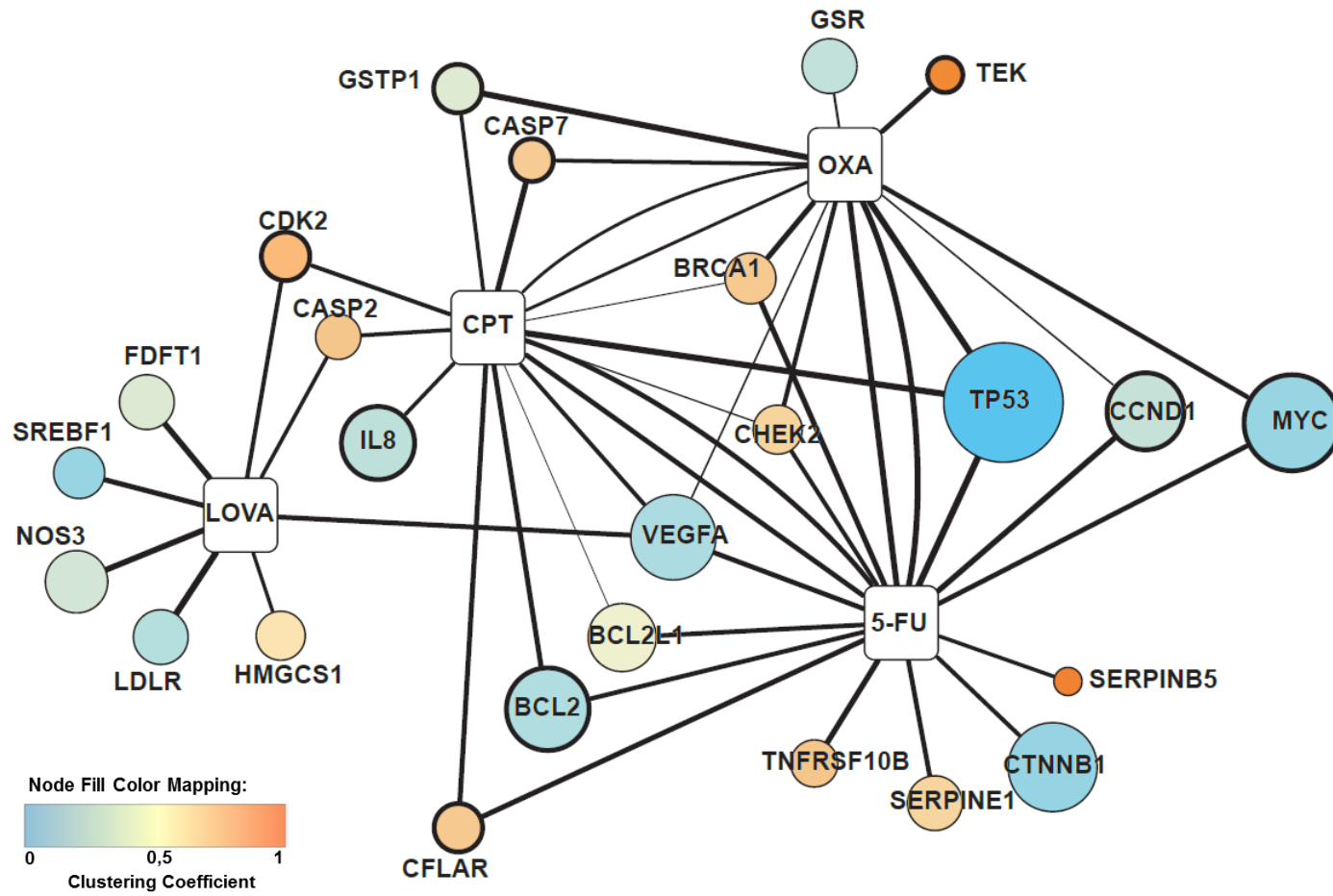

Supplement: Supplementary file 10 — Gene-Drug interaction network. Interactions were retrieved in STITCH v5 (Search Tools for Interactions of Chemicals) between deregulated genes in CRC and 4 drugs: 3 chemotherapeutic drugs used in CRC treatment (5-Fluorouracile (5-FU), Oxaliplatin (OXA) and Camptothecin (CPT), and Lovastatin (LOVA). To facilitate network visualization, we have hidden edges between genes. Node size and node color reflect the interaction degree and the clustering coefficient characterizing nodes in 111 genes based-network presented in Additional file 8. The thickness of edges was correlated with score confidence (large for high score) retrieved in STITCH database (version 5) and the blue color of edges indicated a physical interaction in the source database. The bold line of shape node characterizes genes deregulated in more than 75% of CRC. (PDF 93 kb) [file 12864_2017_4139_MOESM10_ESM.pdf]
