# Supplementary material for: A transcriptome-based protein network that identifies new therapeutic targets in colorectal cancer
Source: BMC Genomics. 2017 Sep 30;18:758. doi: 10.1186/s12864-017-4139-y (PMC5622428; doi:10.1186/s12864-017-4139-y)
Supplement: Supplementary file 3 — Comparative differential expression data focused on genes identified on RT² Profiler™ PCR Array analysis and previously published data. Comparative expression data of selected genes deregulated in CRC as compared to NT in RT² Profiler™ PCR Array analysis, related to Apoptosis (2A, n=14), Cancer Pathway (2B, n= 33), Lipoprotein signaling and Cholesterol metabolism (2C, n=29), Drug metabolism (2D, n=23) and Wnt signaling pathway (2E, n=218). Differential expression data information was listed for each gene: i) from our study performed by PCR array technology in CRC (n=95) as compared to NT (n=19); ii) from our previous study performed with whole-genome microarray technology in CRC (n=25), CRA (n=55) and NT paired to CRA or CRC (n=27) [16]; iii) from microarray expression data of COADREAD cohort of TCGA consortium (153 colon and 69 rectal carcinomas as compared to 22 NT) ([17], gdac.broadinstitute.org) iv) from literature data by focusing on expression analysis obtained in CRC (RNA or protein level) and v) from other literature data obtained from genetic association, epigenetic and functional studies. Underlined gene names indicated that the genes were largely referenced in PubMed in association with colorectal cancer. (DOCX 299 kb) [file 12864_2017_4139_MOESM3_ESM.docx]

**Additional file 3: Table S2.** **Comparative differential expression data focused on genes identified on RT² Profiler™ PCR Array analysis and previously published data.**

Table S2.A. Comparative expression data of selected genes deregulated in CRC as compared to NT in RT² Profiler™ PCR Array analysis, related to Apoptosis (n=14).

| **Gene Symbol** | **PCR Array Data** | | **Microarray Data [1]** | | **TCGA Microarray Data [2]** | | **Bibliographic Data (Expression Analysis)** | | | **Other Bibliographic Data** |
| --- | --- | --- | --- | --- | --- | --- | --- | --- | --- | --- |
|  | **Fold Change CRC vs NT** | **q-value** | **Fold Change CRA vs NT** | **Fold Change CRC vs NT** | **Fold Change CRC vs NT** | **q-value** | **Expression Change** | **RNA and/or Protein Level** | **Ref.** |  |
| BCL2L1 | 2.50 | < 0.001 | 1.56 | / | 1.33 | < 0.001 | ↑ CRC vs NT | RNA, Protein | [3] | Protein expression associated with 20q gain [4]. |
| TNFRSF11B | 2.44 | < 0.01 | 2.61 | / | 3.73 | < 0.001 | ↑ CRC vs NT, ↑ metastasized CRC | RNA, Protein | [5] | / |
| TP53 | 2.20 | < 0.05 | / | / | 1.72 | < 0.001 | ↑ CRC vs NT | RNA, Protein | [6] | TP53 mutations higher in metastases as compared to primary tumors [7]. |
| TNFRSF10B | 1.96 | < 0.001 | 2.34 | / | 2.17 | < 0.001 | ↑ CRC vs NT | RNA, Protein | [8-9] | DR5 expression decreased progressively with CRC stage [10]. |
| RIPK2 | 1.95 | < 0.001 | / | 2.63 | 2.61 | < 0.001 | / | / | / | / |
| LTBR | 1.81 | < 0.05 | / | / | 1.20 | < 0.05 | / | / | / | / |
| CASP2 | 1.71 | < 0.001 | / | / | 1.29 | < 0.001 | / | / | / | / |
| CD27 | -2.30 | < 0.001 | -2.52 | -3.91 | -2.40 | < 0.001 | / | / | / | / |
| CASP7 | -2,30 | < 0.001 | / | -2.66 | -3.15 | < 0.001 | ↓ CRC vs NT | Protein | [11] | / |
| CASP5 | -2,24 | < 0.001 | / | / | -1.53 | < 0.001 | ↑ CRC vs NT | Protein | [12] | Mutated in microsatellite instability (MSI)-positive colon carcinoma [13]. |
| BCL2 | -2,18 | < 0.001 | -2.71 | -5.62 | -4.76 | < 0.001 | ↑ CRC vs NT | RNA, Protein | [14] | / |
| NAIP | -2,15 | < 0.001 | / | -2.54 | -1.17 | < 0.05 | ↓ CRC vs NT | RNA | [15] | Transgenic mouse study shows that NAIP protect against colonic tumorigenesis [16]. |
| CD40LG | -2,09 | < 0.001 | / | / | -1.59 | < 0.001 | / | / | / | / |
| FAS | -1,85 | < 0.001 | / | / | -1.82 | < 0.001 | ↓ CRC vs NT | RNA | [17] | Promoter hypermethylation in 53% of CRC [17]. |

Table S2.B. Comparative expression data of selected genes deregulated in CRC as compared to NT in RT² Profiler™ PCR Array analysis, related to Cancer Pathway (n=33).

| **Gene Symbol** | **PCR Array Data** | | **Microarray Data [1]** | | **TCGA Microarray Data [2]** | | **Bibliographic Data (Expression Analysis)** | | | **Other Bibliographic Data** |
| --- | --- | --- | --- | --- | --- | --- | --- | --- | --- | --- |
|  | **Fold Change CRC vs NT** | **q-value** | **Fold Change CRA vs NT** | **Fold Change CRC vs NT** | **Fold Change CRC vs NT** | **q-value** | **Expression Change** | **RNA and/or Protein Level** | **Ref.** |  |
| IL8 | 15.99 | < 0.001 | / | / | 31,55 | < 0.001 | ↑ CRC, CRA vs NT | RNA, protein | [18-19] | Increased expression of IL-8 in the tumor microenvironment enhanced colon cancer growth and metastasis (transgenic mouse study) [20]. |
| MMP1 | 11.86 | < 0.001 | 9.31 | 26.3 | 28,11 | < 0.001 | ↑ liver metastasis vs primary CRC. | RNA | [21] | Increased immuno-reactivity in carcinomatous vs. adenomatous epithelium of polyps [22]; Relation between MMP1 -1607 ins/del G polymorphisms and risk of small adenoma [23]; Polymorphism MMP1 -1607 1G>2G is significantly associated with a significantly increased risk of cancers [24]. |
| MYC | 7.6 | < 0.001 | 2.83 | / | 3,78 | < 0.001 | ↑ CRC vs NT | RNA | [25] | Genomic amplification of 8q24 in CRC [26]. |
| SERPINB5 | 4.70 | < 0.01 | / | / | 9,82 | < 0.001 | ↑ CRC, CRA vs NT | RNA | [27] | Marker for detection of circulating tumor cells in peripheral blood of colorectal cancer patients [28]. |
| NME1 | 4.59 | < 0.001 | / | 2.14 | 1,84 | < 0.001 | ↑ CRC vs NT | RNA, Protein | [29-30] | / |
| TIMP1 | 3.95 | < 0.001 | 2.11 | 4.39 | 3,66 | < 0.001 | ↑ liver metastasis or invasive CRC vs primary CRC. | RNA, Protein | [21, 31] | Increased immuno-reactivity in carcinomatous vs. adenomatous epithelium of polyps [22]. |
| ITGA2 | 3.83 | < 0.001 | / | / | 2,97 | < 0.001 | ↑ CRC with liver metastasis | Protein | [32] | The ITGA2 807C>T polymorphism was associated with reduced colorectal cancer risk [33]. |
| MET | 3.80 | < 0.001 | 3.1 | / | 3,21 | < 0.001 | ↑ CRC vs NT | RNA | [34] | High c-Met expression levels could predict a poor prognosis in colorectal cancer patients [35] (meta-analysis). |
| ETS2 | 2.78 | < 0.001 | 2.53 | / | 1,34 | < 0.01 | ↑ CRC vs NT | Protein | [36] | / |
| CDK4 | 2.50 | < 0.001 | 2.3 | 2.44 | 2,52 | < 0.001 | ↑ CRC vs NT, CRA | Protein | [37] | / |
| SERPINE1 | 2.43 | < 0.05 | / | / | 2,47 | < 0.001 | ↑ CRC vs NT | Protein | [38-39] | Overexpression related to tumor invasiveness and aggressiveness [38]. |
| TP53 | 2.35 | < 0.05 | / | / | 1,72 | < 0.001 | ↑ CRC vs NT | RNA, Protein | [6] | TP53 mutations higher in metastases as compared to primary tumors [7]. |
| CDK2 | 2.34 | < 0.001 | / | / | 2,06 | < 0.001 | ↑ CRC vs NT | Protein | [40] | / |
| EPDR1 | 2.25 | < 0.01 | / | / | 2,07 | < 0.001 | / | / | / | Relationship between KRAS mutation (G13D) and expression of EPDR1 (cell lines study) [41]. |
| PLAU | 2.17 | < 0.01 | / | / | 3,11 | < 0.001 | ↑ in advanced CRC | Protein | [42] | / |
| BCL2L1 | 2.04 | < 0.001 | 1.56 | / | 1,33 | < 0.001 | ↑ CRC vs NT | RNA, Protein | [3] | Protein expression associated with 20q gain [4]. |
| VEGFA | 1.96 | < 0.001 | / | / | 2,33 | < 0.001 | ↑ CRC vs NT | RNA, Protein | [43-44] | Known as therapeutic target in CRC (Bevacizumab); Differential splicing in CRC [45]. |
| NME4 | 1.95 | < 0.01 | 2.29 | / | 1,20 | n.s | ↑ CRC vs NT | RNA, Protein | [46-47] | / |
| BRCA1 | 1.88 | < 0.05 | / | 2.1 | 1,96 | < 0.001 | ↑ CRC vs NT | RNA | [48] | / |
| CHEK2 | 1.87 | < 0.001 | / | / | 1,62 | < 0.001 | ↓ CRC vs NT | Protein | [49] | Expression of activated CHEK2 increase with tumor invasivity [49]. |
| TNFRSF10B | 1.78 | < 0.001 | 2.34 | / | 2,17 | < 0.001 | ↑ CRC vs NT | RNA, Protein | [8-9] | DR5 expression decreased progressively with CRC stage [10]. |
| CCNE1 | 1.74 | < 0.001 | / | 2.2 | 2,14 | < 0.001 | ↑ CRC vs NT | Protein | [50] | / |
| E2F1 | 1.74 | < 0.05 | 2.4 | 4.39 | 2,03 | < 0.001 | ↑ CRC vs NT | RNA, Protein | [51-52] | E2F1 protein expression was inversely correlated with tumor growth, suggesting a tumor suppressive behavior in CRC [53-54]. |
| CDC25A | 1.72 | < 0.01 | / | / | 1,89 | < 0.001 | ↑ CRC vs NT | Protein | [55] | / |
| IGF1 | -4.31 | < 0.001 | -5.16 | -6.175 | -4,73 | < 0.001 | ↑ CRC vs NT | RNA, Protein | [56-57] | IGF1(CA)19 polymorphism is a candidate gene polymorphism for cancer susceptibility [58]. |
| FGFR2 | -3.30 | < 0.001 | -2.43 | -4.1 | -3,82 | < 0.001 | ↑ CRC vs NT | Protein | [59] | / |
| BCL2 | -3.13 | < 0.001 | -2.71 | -5.62 | -4,76 | < 0.001 | ↑ CRC vs NT | RNA and Protein | [14] | / |
| SNCG | -3.07 | < 0.001 | / | / | -1,35 | < 0.001 | ↑ CRC vs NT, CRA | Protein | [60] | Associated to poor prognosis of CRC [61-62]. |
| CFLAR | -2.54 | < 0.001 | / | / | -1,15 | < 0.01 | ↑ CRC and CRA vs NT | RNA, Protein | [63-64] | / |
| TEK | -2.30 | < 0.001 | -3.3 | / | -2,14 | < 0.001 | / | / | / | / |
| ITGA4 | -2.13 | < 0.001 | / | / | -1,37 | < 0.001 | / | / | / | Promoter hypermethylation in CRC [65-66]. |
| ITGB3 | -2.11 | < 0.001 | / | / | -1,47 | < 0.01 | / | / | / | ITGB3 rs2317676 SNP were associated with an increased risk of CRC [67]. |
| FAS | -1.74 | < 0.001 | / | / | -1,82 | < 0.001 | ↓ CRC vs NT | RNA | [17] | Promoter hypermethylation in 53% of CRC [17]. |

Table S2.C. Comparative expression data of selected genes deregulated in CRC as compared to NT in RT² Profiler™ PCR Array analysis, related to Lipoprotein signaling and Cholesterol metabolism (n=29).

| **Gene Symbol** | **PCR Array Data** | | **Microarray Data [1]** | | **TCGA Microarray Data [2]** | | **Bibliographic Data (Expression Analysis)** | | | **Other Bibliographic Data** |
| --- | --- | --- | --- | --- | --- | --- | --- | --- | --- | --- |
|  | **Fold Change CRC vs NT** | **q- value** | **Fold Change CRA vs NT** | **Fold Change CRC vs NT** | **Fold Change CRC vs NT** | **q-value** | **Expression Change** | **RNA and/or Protein Level** | **Ref.** |  |
| CEL | 15.08 | < 0.001 | / | / | 2,26 | < 0.001 | / | / | / | / |
| PCSK9 | 6.28 | < 0.001 | / | 8.68 | 5,11 | < 0.001 | / | / | / | / |
| INSIG1 | 3.69 | < 0.001 | / | / | 2,49 | < 0.001 | / | / | / | / |
| DHCR7 | 3.56 | < 0.001 | 2.12 | 2.8 | 2,23 | < 0.001 | / | / | / | No association observed between DHCR7 SNP (genetic markers of circulating 25(OH)D) and colorectal cancer [68]. |
| CYP39A1 | 3.35 | < 0.001 | / | / | 3,61 | < 0.001 | / | / | / | / |
| APOL1 | 2.97 | < 0.001 | / | / | 1,21 | n.s | / | / | / | / |
| CYP51A1 | 2.95 | < 0.001 | / | / | 1,29 | < 0.01 | / | / | / | / |
| CXCL16 | 2.80 | < 0.001 | 2.54 | 2.3 | 1,25 | n.s | ↑ CRC vs NT | Protein | [69] | / |
| HMGCS1 | 2.79 | < 0.001 | / | / | 1,29 | n.s | / | / | / | / |
| LDLR | 2.68 | < 0.001 | / | / | 1,34 | n.s | ↑ CRC vs NT | RNA, Protein | [70-72] | / |
| NSDHL | 2.61 | < 0.001 | / | / | 1,63 | < 0.001 | / | / | / | / |
| DHCR24 | 2.44 | < 0.01 | / | / | -1,08 | n.s | / | / | / | / |
| FDPS | 2.40 | < 0.001 | / | / | 1,20 | < 0.05 | / | / | / | / |
| IDI1 | 2.37 | < 0.001 | / | / | 1,01 | n.s | / | / | / | / |
| CNBP | 2.09 | < 0.001 | / | / | -1,22 | < 0.001 | / | / | / | / |
| FDFT1 | 2.01 | < 0.01 | / | / | 1,39 | < 0.01 | / | / | / | / |
| TM7SF2 | 1.96 | < 0.001 | / | / | 1,27 | < 0.001 | / | / | / | / |
| SREBF1 | 1.93 | < 0.001 | / | / | 1,05 | n.s | / | / | / | / |
| SORL1 | 1.90 | < 0.01 | / | / | -1,57 | < 0.001 | / | / | / | / |
| INSIG2 | 1.74 | < 0.001 | / | / | 1,19 | < 0.05 | ↑ CRC vs NT | RNA | [73] | Expression correlates with CRC metastasis [74]. |
| MVK | 1.72 | < 0.001 | / | / | 1,03 | n.s | / | / | / | / |
| STAB1 | -3.52 | < 0.001 | -3.57 | -4.33 | -2,18 | < 0.001 | ↓ CRC vs NT | RNA | [75] | / |
| COLEC12 | -3.32 | < 0.001 | -7.34 | / | -3,64 | < 0.001 | / | / | / | / |
| NR1H4 | -2.66 | < 0.001 | / | / | -7,86 | < 0.001 | ↓ CRC vs NT | RNA, Protein | [76-77] | Promoter methylation in 12% of colon cancer [76]. |
| OSBPL1A | -2.16 | < 0.001 | -2.6 | -3.44 | -2,14 | < 0.001 | ↓ CRC vs NT (short variant) | RNA | [78] | / |
| CELA3A | -2.04 | < 0.001 | / | / | -2,45 | < 0.001 | / | / | / | / |
| PRKAA2 | -1.96 | < 0.001 | / | / | -1,10 | < 0.05 | / | / | / | / |
| CYP11A1 | -1.85 | < 0.001 | / | / | -1,12 | < 0.001 | / | / | / | / |
| STAB2 | -1.68 | < 0.001 | / | / | -3,93 | < 0.001 | / | / | / | 12q13-24 chromosomal deletion in CRC [79]. |

Table S2.D. Comparative expression data of selected genes deregulated in CRC as compared to NT in RT² Profiler™ PCR Array analysis, related to Drug metabolism (n=23).

| **Gene Symbol** | **PCR Array Data** | | **Microarray Data [1]** | | **TCGA Microarray Data [2]** | | **Bibliographic Data (Expression Analysis)** | | | **Other Bibliographic Data** |
| --- | --- | --- | --- | --- | --- | --- | --- | --- | --- | --- |
|  | **Fold Change CRC vs NT** | **q-value** | **Fold Change CRA vs NT** | **Fold Change CRC vs NT** | **Fold Change CRC vs NT** | **q-value** | **Expression Change** | **RNA and/or Protein Level** | **Ref.** |  |
| GPX2 | 5.04 | < 0.001 | 2.25 | / | 1,95 | < 0.001 | ↑ CRC vs NT | Protein ; RNA | [80-81] | / |
| GSTP1 | 3.54 | < 0.001 | 2.63 | / | 1,51 | < 0.001 | ↑ CRC vs NT | Protein ; RNA | [82-83] | Knockdown of GSTP-1 promoted the sensitivity of SNU-407 cells to the anticancer agent 5-fluorouracil [82]. |
| COMT | 3.30 | < 0.001 | 1.72 | / | 1,91 | < 0.001 | ↓ CRC vs NT | Protein | [84] | COMT has tumor-suppressive functions for CRC cell lines in vitro and in in vivo experiments [85]. |
| GPI | 2.78 | < 0.001 | / | / | 1,16 | < 0.05 | ↑ CRC vs NT | RNA | [86] | Overexpression of PGI (DLD-1 cells) significantly contributes to the aggressive phenotype of human colon cancer [87]. |
| ABCC1 | 2.77 | < 0.001 | 2.43 | 2.48 | 2,28 | < 0.001 | ↑ CRC vs NT | RNA | [88] | / |
| PKM2 | 2.77 | < 0.001 | 2.28 | 2.17 | 1,38 | < 0.001 | ↑ CRC vs NT | RNA | [86, 89] | Critical for CRC cell migration and adhesion by regulation of STAT3 signaling [90]; Role in EMT transition [91]. |
| NOS3 | 2.75 | < 0.001 | / | / | 1,09 | n.s | ↑ CRC vs NT | RNA, Protein | [92] | / |
|  |  |  |  |  |  |  | ↓ CRC vs NT | Protein | [93] |  |
| CYP2B6 | 2.61 | < 0.05 | / | / | 1,08 | n.s | / | / | / | / |
| GPX1 | 2.58 | < 0.001 | / | / | 1,37 | < 0.001 | ↑ CRC vs NT | RNA, Protein | [94] | / |
|  |  |  |  |  |  |  | ↓ CRC vs NT | RNA, Protein | [80, 95] |  |
| SRD5A1 | 2.37 | < 0.001 | / | / | 1,03 | n.s | / | / | / | / |
| GPX4 | 2.00 | < 0.001 | / | / | 1,23 | < 0.05 | ↑ CRC vs NT | Protein | [94] | GPx4 T/C 718 SNP is functional and T genotype is associated with lower risk of CRC [96]. |
| AHR | 1.98 | < 0.001 | / | / | 1,85 | < 0.001 | ↑ CRC vs NT | Protein | [97] | / |
| NQO1 | 1.96 | < 0.05 | 2.54 |  | 1,64 | < 0.05 | ↑ CRC vs NT | Protein | [98] | / |
| GSR | 1.82 | < 0.05 | / | / | -1,20 | < 0.001 | / | / | / | / |
| ADH1B | -14.66 | < 0.001 | / | / | -5,90 | < 0.001 | ↓ CRC vs NT | Protein | [99] | / |
| ADH1C | -6.05 | < 0.001 | -9.37 | -34.56 | -2,69 | < 0.001 |  |  |  | / |
| GSTM5 | -3.92 | < 0.001 | -4.13 | -3.29 | -3,84 | < 0.001 | / | / | / | Curcumin may suppress GSTM5 expression to enhance the lethal effect of irinotecan on LOVO cells [100]. |
| HSD17B2 | -3.39 | < 0.001 | -6.33 | -13.56 | -7,63 | < 0.001 | ↓ CRC vs NT | RNA | [101] | / |
| GSTM2 | -3.10 | < 0.001 | -2.21 | / | -1,92 | < 0.001 | / | / | / | Butyrate is an efficient inducer of GSTs and especially of GSTM2 and may act chemo-protectively by increasing detoxification capabilities in the colon mucosa [102]. |
| GPX3 | -2.88 | < 0.001 | -5.75 | -4.13 | -3,40 | < 0.001 | ↓ CRC vs NT | Protein | [80] | Has a tumor suppressor function (model of Gpx3-deficient mice) [103]. |
| GSTM3 | -2.49 | < 0.001 | / | / | -1,65 | < 0.001 | / | / | / | Increased protein expression in CRC with lymph node metastasis as compared to CRC without metastasis [104]; GSTM3 AA (compared with other GSTM3 genotypes combined) had longer disease-free survival [105]. |
| CYP2C19 | -1.94 | < 0.001 | / | / | -1,48 | < 0.01 | / | / | / | No relationship between CYP2C19 polymorphism and susceptibility to CRC [106]. |
| ALOX15 | -1.75 | < 0.01 | / | / | 1,17 | n.s | ↓ CRC vs NT | Protein | [107] | Acts as a tumor suppressor gene [108]. |

Table S2.E. Comparative expression data of selected genes deregulated in CRC as compared to NT in RT² Profiler™ PCR Array analysis, related to Wnt signaling pathway (n=18).

| **Gene Symbol** | **PCR Array Data** | | **Microarray Data [1]** | | **TCGA Microarray Data [2]** | | **Bibliographic Data (Expression Analysis)** | | | **Other Bibliographic Data** |
| --- | --- | --- | --- | --- | --- | --- | --- | --- | --- | --- |
|  | **Fold Change CRC vs NT** | **q-value** | **Fold Change CRA vs NT** | **Fold Change CRC vs NT** | **Fold Change CRC vs NT** | **q-value** | **Expression Change** | **RNA and/or Protein Level** | **Ref.** |  |
| MYC | 8.86 | < 0.001 | 2.83 | / | 3,78 | < 0.001 | ↑ CRC vs NT | RNA | [25] | Genomic amplification of 8q24 in CRC [26]. |
| NKD1 | 6.73 | < 0.01 | 4.0 | / | 14,23 | < 0.001 | ↑ CRC vs NT | RNA | [109-110] | NKD1 mutations promote Wnt-dependent tumorigenesis in a subset of DNA mismatch-repair-deficient colorectal adenocarcinomas [111]. |
| WNT2 | 5.96 | < 0.01 | / | / | 13,15 | < 0.001 | ↑ CRC vs NT | RNA, Protein | [112-113] | siRNA silencing of WNT2 induces apoptosis of human colorectal cancer cells [114] ; Hypermethylated in CRC [115]. |
| FOSL1 | 4.11 | < 0.01 | / | / | 1,80 | < 0.001 | ↑ CRC vs NT | Protein | [116-117] | / |
| CCND1 | 3.97 | < 0.001 | 2.2 | 2.61 | 2,42 | < 0.001 | ↑ CRC vs NT | Protein | [118] | / |
| WISP1 | 2.68 | < 0.05 | / | / | 4,39 | < 0.001 | ↑ CRC vs NT | RNA, protein | [119] | Genomic DNA was amplified in colon cancer cell lines and in human colon tumors [120]. |
| LEF1 | 2.68 | < 0.01 | 2.37 | / | 2,77 | < 0.001 | ↑ CRC vs NT | RNA, Protein | [121-122] | Knockdown of LEF1 expression inhibited colon cancer growth in vitro and in vivo [121]. |
| TCF7 | 2.60 | < 0.001 | 3.56 | / | 2,21 | < 0.001 | ↑ CRC vs NT | Protein | [123] | / |
| CTNNB1 | 2.07 | < 0.001 | / | / | 1,34 | < 0.05 | ↑ CRC, CRA vs NT | Protein | [124] | Activating mutations of CTNNB1 in 80% of cases in TCGA cohort [2]. |
| WNT3 | 2.00 | < 0.05 | / | / | 2,61 | < 0.001 | CRC ≈ NT | RNA | [125] | / |
| FZD6 | 1.84 | < 0.001 | / | / | 2,08 | < 0.001 | ↑ CRC vs NT | RNA, Protein | [126] | / |
| SFRP1 | -12.43 | < 0.001 | -43.335 | -35.525 | -10,37 | < 0.001 | ↓ CRC, CRA vs NT | RNA | [127-128] | Promoter hypermethylation in colorectal tumors [127]. |
| WNT2B | -2.19 | < 0.001 | -4.96 | -2.42 | -3,07 | < 0.001 | / | / | / | / |
| TCF7L1 | -1.95 | < 0.001 | -1.58 | / | -1,47 | < 0.01 | ↑ stage II and III CRC | RNA and Protein | [129] | Hypermethylated in CRC [115]. |
| WNT5B | -1.83 | < 0.001 | / | -3.91 | -1,88 | < 0.001 | / | / | / | Minor allele of WNT5B rs2010851 T>G was significantly associated with a shorter Time Tumor Recurrence in high-risk stage II CC patients [130]. |
| RHOU | -1.78 | < 0.001 | -2.23 | / | -2,47 | < 0.001 | / | / | / | / |
| FRZB | -1.73 | < 0.01 | -2.97 | -4.24 | -1,72 | < 0.001 | / | / | / | Up-regulated in colon cancer hepatic metastasis (poor prognosis predictor) [131]. |
| WNT9A | -1.68 | < 0.001 | / | / | -1,20 | < 0.01 | / | / | / | Up-regulated in different cancer cell lines (gastric, pancreatic, breast) [132]. |

**References**

1. Pesson M, Volant A, Uguen A, Trillet K, De La Grange P, Aubry M, Daoulas M, Robaszkiewicz M, Le Gac G, Morel A *et al*: **A gene expression and pre-mRNA splicing signature that marks the adenoma-adenocarcinoma progression in colorectal cancer**. *PLoS One* 2014, **9**(2):e87761.

2. Cancer Genome Atlas N: **Comprehensive molecular characterization of human colon and rectal cancer**. *Nature* 2012, **487**(7407):330-337.

3. Jin-Song Y, Zhao-Xia W, Cheng-Yu L, Xiao-Di L, Ming S, Yuan-Yuan G, Wei D: **Prognostic significance of Bcl-xL gene expression in human colorectal cancer**. *Acta Histochem* 2011, **113**(8):810-814.

4. Sillars-Hardebol AH, Carvalho B, Belien JA, de Wit M, Delis-van Diemen PM, Tijssen M, van de Wiel MA, Ponten F, Fijneman RJ, Meijer GA: **BCL2L1 has a functional role in colorectal cancer and its protein expression is associated with chromosome 20q gain**. *J Pathol* 2012, **226**(3):442-450.

5. Tsukamoto S, Ishikawa T, Iida S, Ishiguro M, Mogushi K, Mizushima H, Uetake H, Tanaka H, Sugihara K: **Clinical significance of osteoprotegerin expression in human colorectal cancer**. *Clin Cancer Res* 2011, **17**(8):2444-2450.

6. Kowalczyk AE, Krazinski BE, Godlewski J, Kiewisz J, Kwiatkowski P, Sliwinska-Jewsiewicka A, Kiezun J, Sulik M, Kmiec Z: **Expression of the EP300, TP53 and BAX genes in colorectal cancer: Correlations with clinicopathological parameters and survival**. *Oncol Rep* 2017, **38**(1):201-210.

7. Dienstmann R, Elez E, Argiles G, Matos I, Sanz-Garcia E, Ortiz C, Macarulla T, Capdevila J, Alsina M, Sauri T *et al*: **Analysis of mutant allele fractions in driver genes in colorectal cancer - biological and clinical insights**. *Mol Oncol* 2017.

8. Oikonomou E, Kosmidou V, Katseli A, Kothonidis K, Mourtzoukou D, Kontogeorgos G, Andera L, Zografos G, Pintzas A: **TRAIL receptor upregulation and the implication of KRAS/BRAF mutations in human colon cancer tumors**. *Int J Cancer* 2009, **125**(9):2127-2135.

9. Jalving M, Heijink DM, Koornstra JJ, Boersma-van Ek W, Zwart N, Wesseling J, Sluiter WJ, de Vries EG, Kleibeuker JH, de Jong S: **Regulation of TRAIL receptor expression by beta-catenin in colorectal tumours**. *Carcinogenesis* 2014, **35**(5):1092-1099.

10. Perraud A, Akil H, Nouaille M, Petit D, Labrousse F, Jauberteau MO, Mathonnet M: **Expression of p53 and DR5 in normal and malignant tissues of colorectal cancer: correlation with advanced stages**. *Oncol Rep* 2011, **26**(5):1091-1097.

11. Palmerini F, Devilard E, Jarry A, Birg F, Xerri L: **Caspase 7 downregulation as an immunohistochemical marker of colonic carcinoma**. *Hum Pathol* 2001, **32**(5):461-467.

12. Flood B, Oficjalska K, Laukens D, Fay J, O'Grady A, Caiazza F, Heetun Z, Mills KH, Sheahan K, Ryan EJ *et al*: **Altered expression of caspases-4 and -5 during inflammatory bowel disease and colorectal cancer: Diagnostic and therapeutic potential**. *Clin Exp Immunol* 2015, **181**(1):39-50.

13. Soung YH, Jeong EG, Ahn CH, Kim SS, Song SY, Yoo NJ, Lee SH: **Mutational analysis of caspase 1, 4, and 5 genes in common human cancers**. *Hum Pathol* 2008, **39**(6):895-900.

14. Sun N, Meng Q, Tian A: **Expressions of the anti-apoptotic genes Bag-1 and Bcl-2 in colon cancer and their relationship**. *Am J Surg* 2010, **200**(3):341-345.

15. Endo T, Abe S, Seidlar HB, Nagaoka S, Takemura T, Utsuyama M, Kitagawa M, Hirokawa K: **Expression of IAP family proteins in colon cancers from patients with different age groups**. *Cancer Immunol Immunother* 2004, **53**(9):770-776.

16. Allam R, Maillard MH, Tardivel A, Chennupati V, Bega H, Yu CW, Velin D, Schneider P, Maslowski KM: **Epithelial NAIPs protect against colonic tumorigenesis**. *J Exp Med* 2015, **212**(3):369-383.

17. Manoochehri M, Borhani N, Karbasi A, Koochaki A, Kazemi B: **Promoter hypermethylation and downregulation of the FAS gene may be involved in colorectal carcinogenesis**. *Oncol Lett* 2016, **12**(1):285-290.

18. McLean MH, Murray GI, Stewart KN, Norrie G, Mayer C, Hold GL, Thomson J, Fyfe N, Hope M, Mowat NA *et al*: **The inflammatory microenvironment in colorectal neoplasia**. *PLoS One* 2011, **6**(1):e15366.

19. Nastase A, Paslaru L, Herlea V, Ionescu M, Tomescu D, Bacalbasa N, Dima S, Popescu I: **Expression of interleukine-8 as an independent prognostic factor for sporadic colon cancer dissemination**. *J Med Life* 2014, **7**(2):215-219.

20. Lee YS, Choi I, Ning Y, Kim NY, Khatchadourian V, Yang D, Chung HK, Choi D, LaBonte MJ, Ladner RD *et al*: **Interleukin-8 and its receptor CXCR2 in the tumour microenvironment promote colon cancer growth, progression and metastasis**. *Br J Cancer* 2012, **106**(11):1833-1841.

21. Gentner B, Wein A, Croner RS, Zeittraeger I, Wirtz RM, Roedel F, Dimmler A, Dorlaque L, Hohenberger W, Hahn EG *et al*: **Differences in the gene expression profile of matrix metalloproteinases (MMPs) and their inhibitors (TIMPs) in primary colorectal tumors and their synchronous liver metastases**. *Anticancer Res* 2009, **29**(1):67-74.

22. Jeffery N, McLean MH, El-Omar EM, Murray GI: **The matrix metalloproteinase/tissue inhibitor of matrix metalloproteinase profile in colorectal polyp cancers**. *Histopathology* 2009, **54**(7):820-828.

23. Lievre A, Milet J, Carayol J, Le Corre D, Milan C, Pariente A, Nalet B, Lafon J, Faivre J, Bonithon-Kopp C *et al*: **Genetic polymorphisms of MMP1, MMP3 and MMP7 gene promoter and risk of colorectal adenoma**. *BMC Cancer* 2006, **6**:270.

24. Lu L, Sun Y, Li Y, Wan P: **The polymorphism MMP1 -1607 (1G>2G) is associated with a significantly increased risk of cancers from a meta-analysis**. *Tumour Biol* 2015, **36**(3):1685-1693.

25. Kakisako K, Miyahara M, Uchino S, Adachi Y, Kitano S: **Prognostic significance of c-myc mRNA expression assessed by semi-quantitative RT-PCR in patients with colorectal cancer**. *Oncol Rep* 1998, **5**(2):441-445.

26. Camps J, Nguyen QT, Padilla-Nash HM, Knutsen T, McNeil NE, Wangsa D, Hummon AB, Grade M, Ried T, Difilippantonio MJ: **Integrative genomics reveals mechanisms of copy number alterations responsible for transcriptional deregulation in colorectal cancer**. *Genes Chromosomes Cancer* 2009, **48**(11):1002-1017.

27. Wentzensen N, Wilz B, Findeisen P, Wagner R, Dippold W, von Knebel Doeberitz M, Gebert J: **Identification of differentially expressed genes in colorectal adenoma compared to normal tissue by suppression subtractive hybridization**. *Int J Oncol* 2004, **24**(4):987-994.

28. Findeisen P, Rockel M, Nees M, Roder C, Kienle P, Von Knebel Doeberitz M, Kalthoff H, Neumaier M: **Systematic identification and validation of candidate genes for detection of circulating tumor cells in peripheral blood specimens of colorectal cancer patients**. *Int J Oncol* 2008, **33**(5):1001-1010.

29. Kapitanovic S, Cacev T, Berkovic M, Popovic-Hadzija M, Radosevic S, Seiwerth S, Spaventi S, Pavelic K, Spaventi R: **nm23-H1 expression and loss of heterozygosity in colon adenocarcinoma**. *J Clin Pathol* 2004, **57**(12):1312-1318.

30. Pasz-Walczak G, Salagacka A, Potemski P, Balcerczak E, Kordek R, Mirowski M: **Maspin and Nm23-H1 expression in colorectal cancer**. *Neoplasma* 2010, **57**(2):95-101.

31. Pellegrini P, Contasta I, Berghella AM, Gargano E, Mammarella C, Adorno D: **Simultaneous measurement of soluble carcinoembryonic antigen and the tissue inhibitor of metalloproteinase TIMP1 serum levels for use as markers of pre-invasive to invasive colorectal cancer**. *Cancer Immunol Immunother* 2000, **49**(7):388-394.

32. Yoshimura K, Jain A, Allen HE, Laird LS, Chia CY, Ravi S, Brockstedt DG, Giedlin MA, Bahjat KS, Leong ML *et al*: **Selective targeting of antitumor immune responses with engineered live-attenuated Listeria monocytogenes**. *Cancer Res* 2006, **66**(2):1096-1104.

33. Gerger A, Hofmann G, Langsenlehner U, Renner W, Weitzer W, Wehrschutz M, Wascher T, Samonigg H, Krippl P: **Integrin alpha-2 and beta-3 gene polymorphisms and colorectal cancer risk**. *Int J Colorectal Dis* 2009, **24**(2):159-163.

34. Liu Y, Li Q, Zhu L: **Expression of the hepatocyte growth factor and c-Met in colon cancer: correlation with clinicopathological features and overall survival**. *Tumori* 2012, **98**(1):105-112.

35. Gao H, Guan M, Sun Z, Bai C: **High c-Met expression is a negative prognostic marker for colorectal cancer: a meta-analysis**. *Tumour Biol* 2015, **36**(2):515-520.

36. Ito Y, Takeda T, Okada M, Matsuura N: **Expression of ets-1 and ets-2 in colonic neoplasms**. *Anticancer Res* 2002, **22**(3):1581-1584.

37. Zhao P, Hu YC, Talbot IC: **Expressing patterns of p16 and CDK4 correlated to prognosis in colorectal carcinoma**. *World J Gastroenterol* 2003, **9**(10):2202-2206.

38. Mazzoccoli G, Pazienza V, Panza A, Valvano MR, Benegiamo G, Vinciguerra M, Andriulli A, Piepoli A: **ARNTL2 and SERPINE1: potential biomarkers for tumor aggressiveness in colorectal cancer**. *J Cancer Res Clin Oncol* 2012, **138**(3):501-511.

39. Razik E, Kobierzycki C, Grzegrzolka J, Podhorska-Okolow M, Drag-Zalesinska M, Zabel M, Dziegiel P: **Plasminogen Activation System in Rectal Adenocarcinoma**. *Anticancer Res* 2015, **35**(11):6009-6015.

40. Izawa H, Yamamoto H, Ikeda M, Ikeda K, Fukunaga H, Yasui M, Ikenaga M, Sekimoto M, Monden T, Matsuura N *et al*: **Analysis of cyclin D1 and CDK expression in colonic polyps containing neoplastic foci: a study of proteins extracted from paraffin sections**. *Oncol Rep* 2002, **9**(6):1313-1318.

41. Riffo-Campos AL, Castillo J, Vallet-Sanchez A, Ayala G, Cervantes A, Lopez-Rodas G, Franco L: **In silico RNA-seq and experimental analyses reveal the differential expression and splicing of EPDR1 and ZNF518B genes in relation to KRAS mutations in colorectal cancer cells**. *Oncol Rep* 2016, **36**(6):3627-3634.

42. Halamkova J, Kiss I, Pavlovsky Z, Jarkovsky J, Tomasek J, Tucek S, Hanakova L, Moulis M, Cech Z, Zavrelova J *et al*: **Clinical relevance of uPA, uPAR, PAI 1 and PAI 2 tissue expression and plasma PAI 1 level in colorectal carcinoma patients**. *Hepatogastroenterology* 2011, **58**(112):1918-1925.

43. Cheung N, Wong MP, Yuen ST, Leung SY, Chung LP: **Tissue-specific expression pattern of vascular endothelial growth factor isoforms in the malignant transformation of lung and colon**. *Hum Pathol* 1998, **29**(9):910-914.

44. Tayama M, Furuhata T, Inafuku Y, Okita K, Nishidate T, Mizuguchi T, Kimura Y, Hirata K: **Vascular endothelial growth factor 165b expression in stromal cells and colorectal cancer**. *World J Gastroenterol* 2011, **17**(44):4867-4874.

45. Varey AH, Rennel ES, Qiu Y, Bevan HS, Perrin RM, Raffy S, Dixon AR, Paraskeva C, Zaccheo O, Hassan AB *et al*: **VEGF 165 b, an antiangiogenic VEGF-A isoform, binds and inhibits bevacizumab treatment in experimental colorectal carcinoma: balance of pro- and antiangiogenic VEGF-A isoforms has implications for therapy**. *Br J Cancer* 2008, **98**(8):1366-1379.

46. Hayer J, Engel M, Seifert M, Seitz G, Welter C: **Overexpression of nm23-H4 RNA in colorectal and renal tumours**. *Anticancer Res* 2001, **21**(4A):2821-2825.

47. Seifert M, Welter C, Mehraein Y, Seitz G: **Expression of the nm23 homologues nm23-H4, nm23-H6, and nm23-H7 in human gastric and colon cancer**. *J Pathol* 2005, **205**(5):623-632.

48. Garcia V, Garcia JM, Pena C, Silva J, Dominguez G, Rodriguez R, Maximiano C, Espinosa R, Espana P, Bonilla F: **The GADD45, ZBRK1 and BRCA1 pathway: quantitative analysis of mRNA expression in colon carcinomas**. *J Pathol* 2005, **206**(1):92-99.

49. Stawinska M, Cygankiewicz A, Trzcinski R, Mik M, Dziki A, Krajewska WM: **Alterations of Chk1 and Chk2 expression in colon cancer**. *Int J Colorectal Dis* 2008, **23**(12):1243-1249.

50. Qi F, Yuan Y, Zhi X, Huang Q, Chen Y, Zhuang W, Zhang D, Teng B, Kong X, Zhang Y: **Synergistic effects of AKAP95, Cyclin D1, Cyclin E1, and Cx43 in the development of rectal cancer**. *Int J Clin Exp Pathol* 2015, **8**(2):1666-1673.

51. Kasahara M, Takahashi Y, Nagata T, Asai S, Eguchi T, Ishii Y, Fujii M, Ishikawa K: **Thymidylate synthase expression correlates closely with E2F1 expression in colon cancer**. *Clin Cancer Res* 2000, **6**(7):2707-2711.

52. Yasui W, Fujimoto J, Suzuki T, Ono S, Naka K, Yokozaki H, Tahara E: **Expression of cell-cycle-regulating transcription factor E2F-1 in colorectal carcinomas**. *Pathobiology* 1999, **67**(4):174-179.

53. Bramis J, Zacharatos P, Papaconstantinou I, Kotsinas A, Sigala F, Korkolis DP, Nikiteas N, Pazaiti A, Kittas C, Bastounis E *et al*: **E2F-1 transcription factor immunoexpression is inversely associated with tumor growth in colon adenocarcinomas**. *Anticancer Res* 2004, **24**(5A):3041-3047.

54. Zacharatos P, Kotsinas A, Evangelou K, Karakaidos P, Vassiliou LV, Rezaei N, Kyroudi A, Kittas C, Patsouris E, Papavassiliou AG *et al*: **Distinct expression patterns of the transcription factor E2F-1 in relation to tumour growth parameters in common human carcinomas**. *J Pathol* 2004, **203**(3):744-753.

55. Dixon D, Moyana T, King MJ: **Elevated expression of the cdc25A protein phosphatase in colon cancer**. *Exp Cell Res* 1998, **240**(2):236-243.

56. Liu R, Hu LL, Sun A, Cao YJ, Tang T, Zhang XP, Zhang QH: **mRNA expression of IGF-1 and IGF-1R in patients with colorectal adenocarcinoma and type 2 diabetes**. *Arch Med Res* 2014, **45**(4):318-324.

57. Shiratsuchi I, Akagi Y, Kawahara A, Kinugasa T, Romeo K, Yoshida T, Ryu Y, Gotanda Y, Kage M, Shirouzu K: **Expression of IGF-1 and IGF-1R and their relation to clinicopathological factors in colorectal cancer**. *Anticancer Res* 2011, **31**(7):2541-2545.

58. Quan H, Tang H, Fang L, Bi J, Liu Y, Li H: **IGF1(CA)19 and IGFBP-3-202A/C gene polymorphism and cancer risk: a meta-analysis**. *Cell Biochem Biophys* 2014, **69**(1):169-178.

59. Matsuda Y, Hagio M, Seya T, Ishiwata T: **Fibroblast growth factor receptor 2 IIIc as a therapeutic target for colorectal cancer cells**. *Mol Cancer Ther* 2012, **11**(9):2010-2020.

60. Liu C, Dong B, Lu A, Qu L, Xing X, Meng L, Wu J, Eric Shi Y, Shou C: **Synuclein gamma predicts poor clinical outcome in colon cancer with normal levels of carcinoembryonic antigen**. *BMC Cancer* 2010, **10**:359.

61. Liu C, Qu L, Dong B, Xing X, Ren T, Zeng Y, Jiang B, Meng L, Wu J, Shou C: **Combined phenotype of 4 markers improves prognostic value of patients with colon cancer**. *Am J Med Sci* 2012, **343**(4):295-302.

62. Amsterdam A, Shezen E, Raanan C, Schreiber L, Slilat Y, Fabrikant Y, Melzer E: **Differential staining of gamma synuclein in poorly differentiated compared to highly differentiated colon cancer cells**. *Oncol Rep* 2012, **27**(5):1451-1454.

63. Korkolopoulou P, Saetta AA, Levidou G, Gigelou F, Lazaris A, Thymara I, Scliri M, Bousboukea K, Michalopoulos NV, Apostolikas N *et al*: **c-FLIP expression in colorectal carcinomas: association with Fas/FasL expression and prognostic implications**. *Histopathology* 2007, **51**(2):150-156.

64. Ryu BK, Lee MG, Chi SG, Kim YW, Park JH: **Increased expression of cFLIP(L) in colonic adenocarcinoma**. *J Pathol* 2001, **194**(1):15-19.

65. Chang E, Park DI, Kim YJ, Kim BK, Park JH, Kim HJ, Cho YK, Sohn CI, Jeon WK, Kim BI *et al*: **Detection of colorectal neoplasm using promoter methylation of ITGA4, SFRP2, and p16 in stool samples: a preliminary report in Korean patients**. *Hepatogastroenterology* 2010, **57**(101):720-727.

66. Ahmed D, Danielsen SA, Aagesen TH, Bretthauer M, Thiis-Evensen E, Hoff G, Rognum TO, Nesbakken A, Lothe RA, Lind GE: **A tissue-based comparative effectiveness analysis of biomarkers for early detection of colorectal tumors**. *Clin Transl Gastroenterol* 2012, **3**:e27.

67. Ye P, Li Z, Jiang H, Liu T: **SNPs in microRNA-binding sites in the ITGB1 and ITGB3 3'-UTR increase colorectal cancer risk**. *Cell Biochem Biophys* 2014, **70**(1):601-607.

68. Hiraki LT, Qu C, Hutter CM, Baron JA, Berndt SI, Bezieau S, Brenner H, Caan BJ, Casey G, Chang-Claude J *et al*: **Genetic predictors of circulating 25-hydroxyvitamin d and risk of colorectal cancer**. *Cancer Epidemiol Biomarkers Prev* 2013, **22**(11):2037-2046.

69. Hojo S, Koizumi K, Tsuneyama K, Arita Y, Cui Z, Shinohara K, Minami T, Hashimoto I, Nakayama T, Sakurai H *et al*: **High-level expression of chemokine CXCL16 by tumor cells correlates with a good prognosis and increased tumor-infiltrating lymphocytes in colorectal cancer**. *Cancer Res* 2007, **67**(10):4725-4731.

70. Notarnicola M, Linsalata M, Caruso MG, Cavallini A, Di Leo A: **Low density lipoprotein receptors and polyamine levels in human colorectal adenocarcinoma**. *J Gastroenterol* 1995, **30**(6):705-709.

71. Lum DF, McQuaid KR, Gilbertson VL, Hughes-Fulford M: **Coordinate up-regulation of low-density lipoprotein receptor and cyclo-oxygenase-2 gene expression in human colorectal cells and in colorectal adenocarcinoma biopsies**. *Int J Cancer* 1999, **83**(2):162-166.

72. Caruso MG, Notarnicola M, Cavallini A, Di Leo A: **Low density lipoprotein receptor and mRNA expression in human colorectal cancer**. *Anticancer Res* 2001, **21**(1A):429-433.

73. Eschrich S, Yang I, Bloom G, Kwong KY, Boulware D, Cantor A, Coppola D, Kruhoffer M, Aaltonen L, Orntoft TF *et al*: **Molecular staging for survival prediction of colorectal cancer patients**. *J Clin Oncol* 2005, **23**(15):3526-3535.

74. Sun S, Zhang G, Sun Q, Wu Z, Shi W, Yang B, Li Y: **Insulin-induced gene 2 expression correlates with colorectal cancer metastasis and disease outcome**. *IUBMB Life* 2016, **68**(1):65-71.

75. Kaiser S, Park YK, Franklin JL, Halberg RB, Yu M, Jessen WJ, Freudenberg J, Chen X, Haigis K, Jegga AG *et al*: **Transcriptional recapitulation and subversion of embryonic colon development by mouse colon tumor models and human colon cancer**. *Genome Biol* 2007, **8**(7):R131.

76. Bailey AM, Zhan L, Maru D, Shureiqi I, Pickering CR, Kiriakova G, Izzo J, He N, Wei C, Baladandayuthapani V *et al*: **FXR silencing in human colon cancer by DNA methylation and KRAS signaling**. *Am J Physiol Gastrointest Liver Physiol* 2014, **306**(1):G48-58.

77. Lax S, Schauer G, Prein K, Kapitan M, Silbert D, Berghold A, Berger A, Trauner M: **Expression of the nuclear bile acid receptor/farnesoid X receptor is reduced in human colon carcinoma compared to nonneoplastic mucosa independent from site and may be associated with adverse prognosis**. *Int J Cancer* 2012, **130**(10):2232-2239.

78. Thorsen K, Schepeler T, Oster B, Rasmussen MH, Vang S, Wang K, Hansen KQ, Lamy P, Pedersen JS, Eller A *et al*: **Tumor-specific usage of alternative transcription start sites in colorectal cancer identified by genome-wide exon array analysis**. *BMC Genomics* 2011, **12**:505.

79. Aytekin T, Ozaslan M, Cengiz B: **Deletion mapping of chromosome region 12q13-24 in colorectal cancer**. *Cancer Genet Cytogenet* 2010, **201**(1):32-38.

80. Murawaki Y, Tsuchiya H, Kanbe T, Harada K, Yashima K, Nozaka K, Tanida O, Kohno M, Mukoyama T, Nishimuki E *et al*: **Aberrant expression of selenoproteins in the progression of colorectal cancer**. *Cancer Lett* 2008, **259**(2):218-230.

81. Chiu ST, Hsieh FJ, Chen SW, Chen CL, Shu HF, Li H: **Clinicopathologic correlation of up-regulated genes identified using cDNA microarray and real-time reverse transcription-PCR in human colorectal cancer**. *Cancer Epidemiol Biomarkers Prev* 2005, **14**(2):437-443.

82. Zhang R, Kang KA, Piao MJ, Kim KC, Zheng J, Yao CW, Cha JW, Maeng YH, Chang WY, Moon PG *et al*: **Epigenetic alterations are involved in the overexpression of glutathione S-transferase pi-1 in human colorectal cancers**. *Int J Oncol* 2014, **45**(3):1275-1283.

83. Tan KL, Jankova L, Chan C, Fung CL, Clarke C, Lin BP, Robertson G, Molloy M, Chapuis PH, Bokey L *et al*: **Clinicopathological correlates and prognostic significance of glutathione S-transferase Pi expression in 468 patients after potentially curative resection of node-positive colonic cancer**. *Histopathology* 2011, **59**(6):1057-1070.

84. Pancione M, Remo A, Zanella C, Sabatino L, Di Blasi A, Laudanna C, Astati L, Rocco M, Bifano D, Piacentini P *et al*: **The chromatin remodelling component SMARCB1/INI1 influences the metastatic behavior of colorectal cancer through a gene signature mapping to chromosome 22**. *J Transl Med* 2013, **11**:297.

85. Wu W, Wu Q, Hong X, Xiong G, Xiao Y, Zhou J, Wang W, Wu H, Zhou L, Song W *et al*: **Catechol-O-methyltransferase inhibits colorectal cancer cell proliferation and invasion**. *Arch Med Res* 2015, **46**(1):17-23.

86. Yeh CS, Wang JY, Chung FY, Lee SC, Huang MY, Kuo CW, Yang MJ, Lin SR: **Significance of the glycolytic pathway and glycolysis related-genes in tumorigenesis of human colorectal cancers**. *Oncol Rep* 2008, **19**(1):81-91.

87. Tsutsumi S, Fukasawa T, Yamauchi H, Kato T, Kigure W, Morita H, Asao T, Kuwano H: **Phosphoglucose isomerase enhances colorectal cancer metastasis**. *Int J Oncol* 2009, **35**(5):1117-1121.

88. Hlavata I, Mohelnikova-Duchonova B, Vaclavikova R, Liska V, Pitule P, Novak P, Bruha J, Vycital O, Holubec L, Treska V *et al*: **The role of ABC transporters in progression and clinical outcome of colorectal cancer**. *Mutagenesis* 2012, **27**(2):187-196.

89. Kress S, Stein A, Maurer P, Weber B, Reichert J, Buchmann A, Huppert P, Schwarz M: **Expression of hypoxia-inducible genes in tumor cells**. *J Cancer Res Clin Oncol* 1998, **124**(6):315-320.

90. Yang P, Li Z, Fu R, Wu H, Li Z: **Pyruvate kinase M2 facilitates colon cancer cell migration via the modulation of STAT3 signalling**. *Cell Signal* 2014, **26**(9):1853-1862.

91. Hamabe A, Konno M, Tanuma N, Shima H, Tsunekuni K, Kawamoto K, Nishida N, Koseki J, Mimori K, Gotoh N *et al*: **Role of pyruvate kinase M2 in transcriptional regulation leading to epithelial-mesenchymal transition**. *Proc Natl Acad Sci U S A* 2014, **111**(43):15526-15531.

92. Yagihashi N, Kasajima H, Sugai S, Matsumoto K, Ebina Y, Morita T, Murakami T, Yagihashi S: **Increased in situ expression of nitric oxide synthase in human colorectal cancer**. *Virchows Arch* 2000, **436**(2):109-114.

93. Yu JX, Cui L, Zhang QY, Chen H, Ji P, Wei HJ, Ma HY: **Expression of NOS and HIF-1alpha in human colorectal carcinoma and implication in tumor angiogenesis**. *World J Gastroenterol* 2006, **12**(29):4660-4664.

94. Yagublu V, Arthur JR, Babayeva SN, Nicol F, Post S, Keese M: **Expression of selenium-containing proteins in human colon carcinoma tissue**. *Anticancer Res* 2011, **31**(9):2693-2698.

95. Nalkiran I, Turan S, Arikan S, Kahraman OT, Acar L, Yaylim I, Ergen A: **Determination of gene expression and serum levels of MnSOD and GPX1 in colorectal cancer**. *Anticancer Res* 2015, **35**(1):255-259.

96. Bermano G, Pagmantidis V, Holloway N, Kadri S, Mowat NA, Shiel RS, Arthur JR, Mathers JC, Daly AK, Broom J *et al*: **Evidence that a polymorphism within the 3'UTR of glutathione peroxidase 4 is functional and is associated with susceptibility to colorectal cancer**. *Genes Nutr* 2007, **2**(2):225-232.

97. Ikuta T, Kurosumi M, Yatsuoka T, Nishimura Y: **Tissue distribution of aryl hydrocarbon receptor in the intestine: Implication of putative roles in tumor suppression**. *Exp Cell Res* 2016, **343**(2):126-134.

98. Ji L, Wei Y, Jiang T, Wang S: **Correlation of Nrf2, NQO1, MRP1, cmyc and p53 in colorectal cancer and their relationships to clinicopathologic features and survival**. *Int J Clin Exp Pathol* 2014, **7**(3):1124-1131.

99. Chiang CP, Jao SW, Lee SP, Chen PC, Chung CC, Lee SL, Nieh S, Yin SJ: **Expression pattern, ethanol-metabolizing activities, and cellular localization of alcohol and aldehyde dehydrogenases in human large bowel: association of the functional polymorphisms of ADH and ALDH genes with hemorrhoids and colorectal cancer**. *Alcohol* 2012, **46**(1):37-49.

100. Zhu DJ, Chen XW, Wang JZ, Ju YL, Ou Yang MZ, Zhang WJ: **Proteomic analysis identifies proteins associated with curcumin-enhancing efficacy of irinotecan-induced apoptosis of colorectal cancer LOVO cell**. *Int J Clin Exp Pathol* 2014, **7**(1):1-15.

101. Oduwole OO, Isomaa VV, Nokelainen PA, Stenback F, Vihko PT: **Downregulation of estrogen-metabolizing 17 beta-hydroxysteroid dehydrogenase type 2 expression correlates inversely with Ki67 proliferation marker in colon-cancer development**. *Int J Cancer* 2002, **97**(1):1-6.

102. Ebert MN, Klinder A, Peters WH, Schaferhenrich A, Sendt W, Scheele J, Pool-Zobel BL: **Expression of glutathione S-transferases (GSTs) in human colon cells and inducibility of GSTM2 by butyrate**. *Carcinogenesis* 2003, **24**(10):1637-1644.

103. Barrett CW, Ning W, Chen X, Smith JJ, Washington MK, Hill KE, Coburn LA, Peek RM, Chaturvedi R, Wilson KT *et al*: **Tumor suppressor function of the plasma glutathione peroxidase gpx3 in colitis-associated carcinoma**. *Cancer Res* 2013, **73**(3):1245-1255.

104. Meding S, Balluff B, Elsner M, Schone C, Rauser S, Nitsche U, Maak M, Schafer A, Hauck SM, Ueffing M *et al*: **Tissue-based proteomics reveals FXYD3, S100A11 and GSTM3 as novel markers for regional lymph node metastasis in colon cancer**. *J Pathol* 2012, **228**(4):459-470.

105. Holley SL, Rajagopal R, Hoban PR, Deakin M, Fawole AS, Elder JB, Elder J, Smith V, Strange RC, Fryer AA: **Polymorphisms in the glutathione S-transferase mu cluster are associated with tumour progression and patient outcome in colorectal cancer**. *Int J Oncol* 2006, **28**(1):231-236.

106. Tamer L, Ercan B, Ercan S, Ates N, Ates C, Ocal K, Dirlik M, Aydin S, Atik U: **CYP2C19 polymorphisms in patients with gastric and colorectal carcinoma**. *Int J Gastrointest Cancer* 2006, **37**(1):1-5.

107. Shureiqi I, Wojno KJ, Poore JA, Reddy RG, Moussalli MJ, Spindler SA, Greenson JK, Normolle D, Hasan AA, Lawrence TS *et al*: **Decreased 13-S-hydroxyoctadecadienoic acid levels and 15-lipoxygenase-1 expression in human colon cancers**. *Carcinogenesis* 1999, **20**(10):1985-1995.

108. Il Lee S, Zuo X, Shureiqi I: **15-Lipoxygenase-1 as a tumor suppressor gene in colon cancer: is the verdict in?** *Cancer Metastasis Rev* 2011, **30**(3-4):481-491.

109. Katoh M: **Molecular cloning, gene structure, and expression analyses of NKD1 and NKD2**. *Int J Oncol* 2001, **19**(5):963-969.

110. Stancikova J, Krausova M, Kolar M, Fafilek B, Svec J, Sedlacek R, Neroldova M, Dobes J, Horazna M, Janeckova L *et al*: **NKD1 marks intestinal and liver tumors linked to aberrant Wnt signaling**. *Cell Signal* 2015, **27**(2):245-256.

111. Guo J, Cagatay T, Zhou G, Chan CC, Blythe S, Suyama K, Zheng L, Pan K, Qian C, Hamelin R *et al*: **Mutations in the human naked cuticle homolog NKD1 found in colorectal cancer alter Wnt/Dvl/beta-catenin signaling**. *PLoS One* 2009, **4**(11):e7982.

112. Holcombe RF, Marsh JL, Waterman ML, Lin F, Milovanovic T, Truong T: **Expression of Wnt ligands and Frizzled receptors in colonic mucosa and in colon carcinoma**. *Mol Pathol* 2002, **55**(4):220-226.

113. Park JK, Song JH, He TC, Nam SW, Lee JY, Park WS: **Overexpression of Wnt-2 in colorectal cancers**. *Neoplasma* 2009, **56**(2):119-123.

114. Shi Y, He B, Kuchenbecker KM, You L, Xu Z, Mikami I, Yagui-Beltran A, Clement G, Lin YC, Okamoto J *et al*: **Inhibition of Wnt-2 and galectin-3 synergistically destabilizes beta-catenin and induces apoptosis in human colorectal cancer cells**. *Int J Cancer* 2007, **121**(6):1175-1181.

115. Farkas SA, Vymetalkova V, Vodickova L, Vodicka P, Nilsson TK: **DNA methylation changes in genes frequently mutated in sporadic colorectal cancer and in the DNA repair and Wnt/beta-catenin signaling pathway genes**. *Epigenomics* 2014, **6**(2):179-191.

116. Wang HL, Wang J, Xiao SY, Haydon R, Stoiber D, He TC, Bissonnette M, Hart J: **Elevated protein expression of cyclin D1 and Fra-1 but decreased expression of c-Myc in human colorectal adenocarcinomas overexpressing beta-catenin**. *Int J Cancer* 2002, **101**(4):301-310.

117. Zhang W, Hart J, McLeod HL, Wang HL: **Differential expression of the AP-1 transcription factor family members in human colorectal epithelial and neuroendocrine neoplasms**. *Am J Clin Pathol* 2005, **124**(1):11-19.

118. Arber N, Hibshoosh H, Moss SF, Sutter T, Zhang Y, Begg M, Wang S, Weinstein IB, Holt PR: **Increased expression of cyclin D1 is an early event in multistage colorectal carcinogenesis**. *Gastroenterology* 1996, **110**(3):669-674.

119. Davies SR, Davies ML, Sanders A, Parr C, Torkington J, Jiang WG: **Differential expression of the CCN family member WISP-1, WISP-2 and WISP-3 in human colorectal cancer and the prognostic implications**. *Int J Oncol* 2010, **36**(5):1129-1136.

120. Pennica D, Swanson TA, Welsh JW, Roy MA, Lawrence DA, Lee J, Brush J, Taneyhill LA, Deuel B, Lew M *et al*: **WISP genes are members of the connective tissue growth factor family that are up-regulated in wnt-1-transformed cells and aberrantly expressed in human colon tumors**. *Proc Natl Acad Sci U S A* 1998, **95**(25):14717-14722.

121. Wang WJ, Yao Y, Jiang LL, Hu TH, Ma JQ, Liao ZJ, Yao JT, Li DF, Wang SH, Nan KJ: **Knockdown of lymphoid enhancer factor 1 inhibits colon cancer progression in vitro and in vivo**. *PLoS One* 2013, **8**(10):e76596.

122. Wang WJ, Yao Y, Jiang LL, Hu TH, Ma JQ, Ruan ZP, Tian T, Guo H, Wang SH, Nan KJ: **Increased LEF1 expression and decreased Notch2 expression are strong predictors of poor outcomes in colorectal cancer patients**. *Dis Markers* 2013, **35**(5):395-405.

123. Mayer K, Hieronymus T, Castrop J, Clevers H, Ballhausen WG: **Ectopic activation of lymphoid high mobility group-box transcription factor TCF-1 and overexpression in colorectal cancer cells**. *Int J Cancer* 1997, **72**(4):625-630.

124. Kobayashi M, Honma T, Matsuda Y, Suzuki Y, Narisawa R, Ajioka Y, Asakura H: **Nuclear translocation of beta-catenin in colorectal cancer**. *Br J Cancer* 2000, **82**(10):1689-1693.

125. Nishioka M, Ueno K, Hazama S, Okada T, Sakai K, Suehiro Y, Okayama N, Hirata H, Oka M, Imai K *et al*: **Possible involvement of Wnt11 in colorectal cancer progression**. *Mol Carcinog* 2013, **52**(3):207-217.

126. Kim BK, Yoo HI, Kim I, Park J, Kim Yoon S: **FZD6 expression is negatively regulated by miR-199a-5p in human colorectal cancer**. *BMB Rep* 2015, **48**(6):360-366.

127. Qi J, Zhu YQ, Luo J, Tao WH: **Hypermethylation and expression regulation of secreted frizzled-related protein genes in colorectal tumor**. *World J Gastroenterol* 2006, **12**(44):7113-7117.

128. Fu J, Tang W, Du P, Wang G, Chen W, Li J, Zhu Y, Gao J, Cui L: **Identifying microRNA-mRNA regulatory network in colorectal cancer by a combination of expression profile and bioinformatics analysis**. *BMC Syst Biol* 2012, **6**:68.

129. Li C, Cai S, Wang X, Jiang Z: **Hypomethylation-associated up-regulation of TCF3 expression and recurrence in stage II and III colorectal cancer**. *PLoS One* 2014, **9**(11):e112005.

130. Paez D, Gerger A, Zhang W, Yang D, Labonte MJ, Benhanim L, Kahn M, Lenz F, Lenz C, Ning Y *et al*: **Association of common gene variants in the WNT/beta-catenin pathway with colon cancer recurrence**. *Pharmacogenomics J* 2014, **14**(2):142-150.

131. Shen Y, Zhang F, Lan H, Chen K, Zhang Q, Xie G, Teng L, Jin K: **FRZB up-regulation is correlated with hepatic metastasis and poor prognosis in colon carcinoma patients with hepatic metastasis**. *Int J Clin Exp Pathol* 2015, **8**(4):4083-4090.

132. Kirikoshi H, Sekihara H, Katoh M: **Expression of WNT14 and WNT14B mRNAs in human cancer, up-regulation of WNT14 by IFNgamma and up-regulation of WNT14B by beta-estradiol**. *Int J Oncol* 2001, **19**(6):1221-1225.
